# Grid integration feasibility and investment planning of offshore wind power under carbon-neutral transition in China

**DOI:** 10.1038/s41467-023-37536-3

**Published:** 2023-04-28

**Authors:** Xinyang Guo, Xinyu Chen, Xia Chen, Peter Sherman, Jinyu Wen, Michael McElroy

**Affiliations:** 1 State Key Laboratory of Advanced Electromagnetic Engineering and Technology, School of Electrical and Electronic Engineering, Huazhong University of Science and Technology, Wuhan, China; 2 Harvard John A. Paulson School of Engineering and Applied Sciences, Harvard University, Cambridge, MA USA; 3 Department of Earth and Planetary Sciences, Harvard University, Cambridge, MA USA

**Keywords:** Wind energy, Electrical and electronic engineering, Climate-change mitigation

## Abstract

Offshore wind power, with accelerated declining levelized costs, is emerging as a critical building-block to fully decarbonize the world’s largest CO_2_ emitter, China. However, system integration barriers as well as system balancing costs have not been quantified yet. Here we develop a bottom-up model to test the grid accommodation capabilities and design the optimal investment plans for offshore wind power considering resource distributions, hourly power system simulations, and transmission/storage/hydrogen investments. Results indicate that grid integration barriers exist currently at the provincial level. For 2030, optimized offshore wind investment levels should be doubled compared with current government plans, and provincial allocations should be significantly improved considering both resource quality and grid conditions. For 2050, offshore wind capacity in China could reach as high as 1500 GW, prompting a paradigm shift in national transmission structure, favoring long-term storage in the energy portfolio, enabling green hydrogen production in coastal demand centers, resulting in the world’s largest wind power market.

## Abstract (web summary)

Offshore wind power may play a key role in decarbonising energy supplies. Here the authors evaluates current grid integration capabilities for wind power in China and find that investment levels should be doubled for 2030, and that long-term storage and transmissions are key to accelerated developments of offshore wind in 2050.

## Introduction

The recent IPCC report concludes that if we are to limit the increase in global average surface temperature to 1.5 °C by 2100, global energy systems must be managed to realize neutrality in terms of fossil carbon emissions by mid-century^1^. China, responsible for 30% of global anthropogenic emissions in 2019^2^, committed recently to reach carbon neutrality by 2060 if not sooner^3^. Northern inland China hosts major wind and solar resources and has been the beneficiary over recent years in important related investments. Significant economic activity and population are concentrated, however, in the eastern, coastal, area of the country. Coastal and neighboring provinces, accounting for only 30% of the land mass in China, host 76% of the national population and are responsible for 72% of total national power consumption and for 70% of total CO_2_ emissions^4^. The quantity of CO_2_ released from the region surpasses that from the entire US, and is equivalent to twice that from the EU^2^. How to transition away from the fossil fuel-based energy infrastructure in this coastal area represents not only the core challenge for ambitions to achieve carbon neutrality in China, but also a signature target for plans to achieve GHG mitigation on a larger global scale.

Decarbonizing the economic center of China has faced significant difficulty reflecting the important imbalance geographically between energy consumption and the distribution of renewable resources^5^. With superior wind conditions, northern China is host to 170 GW of onshore wind investment, accounting for over 80% of national and 30% of global wind commitments^6,7^. Wind turbines installed in northern China have suffered however from significant curtailment in output particularly in winter due to low local power demand and limited grid flexibility^8^. Further expansion of wind investments inland is currently limited by a combination of technical and policy restrictions. Additionally, effectively all hydro resources in China are sited in the western region of the country^9^, and optimal conditions for solar installations are located in the northwest^10^.In the absence of local renewable energy sources, nuclear has been considered as the primary solution for decarbonizing the eastern, coastal, area of China. However, nuclear units are not only more expensive and slower to build than alternative sources of power, but also prompt concerns from the public with respect to safety. Construction of nuclear units has fallen behind the agenda promoted originally by the government, in part reflecting reactions to the events at Fukushima, compounded by concurrent unanticipated increases in cost.

**Fig. 1:**
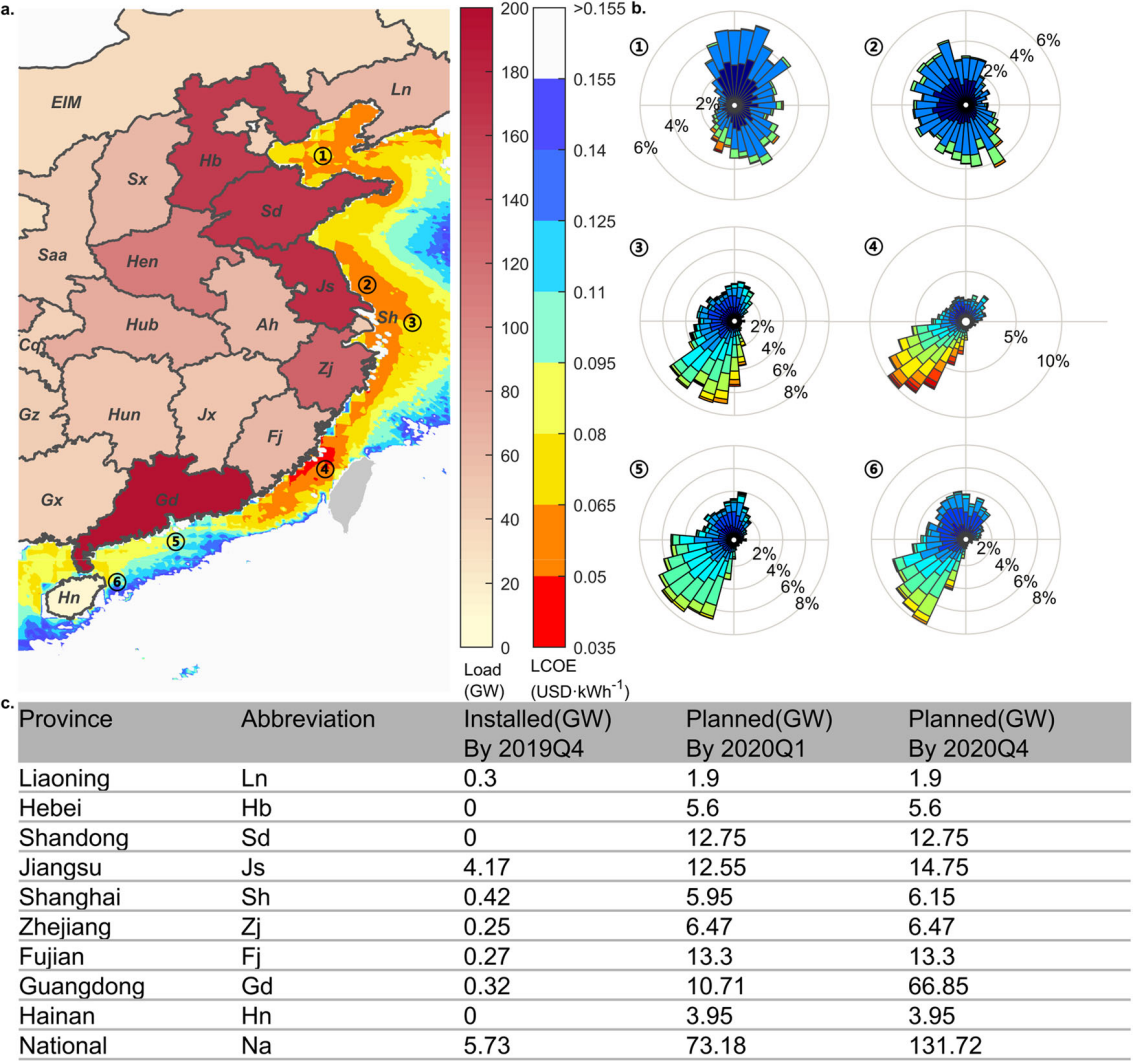
Levelized cost, wind directions and development plan of Offshore wind power in mainland China. **a** provincial peak load and offshore wind levelized costs for mainland China; **b** wind roses at typical locations for offshore wind development, with each location indicated in **a**; **c** offshore wind installation statistics and national planned capacity for all coastal provinces. 2020Q4 plan was incorporated in the analyses.

A rapid development of offshore wind power offers a promising solution to the challenge for decarbonization of coastal regions of China. The installed capacity of offshore wind power has increased by a factor of 15 in Europe over the last decade^11^. As a result, the world average levelized cost for related power has declined from 126 $/MWh in 2017 to 84 $/MWh in 2019^12^. Bid prices for offshore projects in the EU are now in the range of 40–50 $/MWh^11^, competitive for the first time with coal-fired generators^13^. Rights for development of 390 thousand acres of wind resources off the coast of Massachusetts, US, were auctioned at an historically record level of $405 million, greater than revenues raised for allocation of comparable acreage for exploration of opportunities for development of offshore oil^14^. China had less than 6 GW of offshore wind projects in total as of 2019^6^, minor compared with investments in other renewable resources. The landscape however is rapidly changing in response to the ongoing reduction in projected costs. Current plans call for development of more than 130 GW of offshore wind in China over the coming decade (see provincial allocation in Fig. 1b)^15^, equivalent to five times the total of the current global commitment^6^.

Recent literature has focused on analyses of projected levelized costs for electricity supplied from offshore wind resources. Jansen et al. ^16^ reported that auction prices for offshore wind projects in Europe have been competitive recently with prices for conventional power generation. Wiser et al. ^17^ envisages an accelerated decline in offshore wind levelized costs via expert elicitation, projecting a decrease for fixed bottom installations of 35% in 2035 rising to 49% by 2050. Sherman et al. ^18^ quantified the offshore wind physical resources and economics for China, highlighting the significance of the economic potential for the resource on the basis of anticipated levelized costs, and the importance of the contribution it could make to ongoing carbon neutrality planning^19^. Davidson et al. ^20^ conducted the first comprehensive offshore wind potential analyses in 2016. They provided detailed supply curves for both onshore and offshore wind with specific cost breakdowns. However, due to the significantly higher capital expenditures prevailing at that time, they restricted their attention to limited investments designed to minimize costs with an emphasis on commitments close to shore. System integration testing was conducted at a regional level rather than at a provincial scale using heuristic methods due to data limitations. To the best of our knowledge, few analyses have focused on opportunities for province-by-province grid integration at elevated levels of offshore investment awaiting perhaps guidance from the government on future prospects. Restrictions imposed by the government have been particularly important for onshore wind development in China^20^. Lacking systematic analyses for system simulation at an early stage of onshore wind development, major financial losses of $15 billion have been incurred due to curtailment of these resources^21–23^. Since March 2016, the Chinese government has suspended approval for new wind farms in provinces where annual wind curtailment rates have exceeded 20%. In June 2018, the curtailment threshold was tightened further from 20% to 5%, with the exception of relaxed thresholds for three wind rich provinces (15% for Xinjiang and Gansu, 8% for WIM). As a result, the growth rate of wind investment in the “three north” area has declined from an annual rate of over 25% in 2015 (15% in NE, 20% in NC and 40% in NW) to less than 3% in 2019^24^. Had a comprehensive grid integration study been carried out in advance of the earlier investments in onshore wind, it might have been possible to have avoided the major financial losses that occurred subsequently due to curtailment.

Investment planning of renewables is receiving increased attention at the national level. The national government is committed to a mid-term target of 1200 GW of non-hydro renewable installations by 2030. In response, provincial governments have released investment agendas for onshore, offshore wind and solar PV, which aggregate to a total of 1228 GW (refer to Table S23 for collected provincial plans), in line with the national commitment. However, no research has been carried out to test the effectiveness of current government plans, to search for an optimal investment portfolio for renewables in 2030 that could take account of the rapidly changing economics of offshore wind. The optimal provincial allocation of offshore investments, and the trade-offs between offshore and onshore wind have not yet been defined in terms of the long-term transition plan for 2050. Attention must be directed to defining the role offshore wind power can play in the goal for carbon neutrality, the paradigm shift in the national transmission network that will be required to adapt to the accelerated investment in these resources, and the coordinated deployment of long-term storage systems and P2G infrastructures that will be required as a complement.

Here we present a high-resolution assessment model to conduct a system integration analysis designed to develop an optimal deployment plan for offshore wind power in China. The model combines a refined analysis of offshore wind resources and economics, considering the micro siting of wind farms with optimization of delivery systems, and simulations of hourly power system demands, identifying optimal plans for provincial investments in offshore installations, transmissions and storage. First, the physical characteristics and economics of offshore wind projects are analyzed for all possible locations off the coast of mainland China, using hourly meteorological data for the past 40 years available at a resolution of 50 × 50 km^25^ in combination with bathymetric data at a resolution of 15 arc second^26^. Detailed cost models are developed for offshore wind projects considering turbines, foundations, convergence cables, offshore substations, submarine delivery cables and reactive power compensators^27–30^, allowing for cost variations reflecting differences in water depths and distances to shore. Second, hourly grid integration simulations are conducted for a full year at all possible onshore and offshore wind investment levels for all of the provinces in the coastal area and in the wind-rich “Three North” region. The simulations cover 32 provinces in China, include unit level information for over 3000 thermal units, incorporate comprehensive flexibility constraints (including ramping, minimum on-off times and minimal load bounds) and recognize all existing inter-provincial transmissions Third, the analysis further includes an optimal investment model for thermal units, non-hydro renewables, and for transmission and storage systems, intended to complement system investment planning for both 2030 and 2050. For 2030 investment planning, the effectiveness of current government strategy is tested, and an improved renewable investment portfolio is proposed, analyzing trade-offs among offshore wind, onshore counterparts and solar power. For 2050, the role of offshore wind power in the deep-decarbonized energy economy is evaluated, and associated requirements for national transmission infrastructures, long-term storage facilities and P2G developments are analyzed.

The results indicate that at least 1000 GW of offshore wind capacity could already be available at a levelized cost below that for nuclear units in China. Under current power system conditions, grid integration barriers heavily restrict the deployment of onshore wind power in wind rich northern regions, although they are more economical than offshore counterparts. For 2030, the Chinese government has committed to 1200 GW of renewable investments, and the current provincial investment agenda highlights onshore wind (41%) and solar development (43%), complemented with a relatively small contribution from offshore wind (16%, see details in SI). Our optimized plan would double the offshore wind investment in 2030 and avoid the ineffective onshore wind projects in central and southern areas. Current provincial deployment plans for offshore wind are also largely improved, shifting part of the investment from Guangdong to provinces such as Jiangsu and Zhejiang. Our optimized plan could boost the national renewable penetration from 31.5% to 40%, at a cost lower than that anticipated in the current plan. For 2050, offshore wind capacity in China could reach as high as 1500 GW, constituting a major building-block for the carbon neutrality transition in China, promoting development of the world’s largest wind power market. In the meantime, it will prompt a paradigm shift in national transmission structure, favoring long-term storage in the energy portfolio, enabling green hydrogen production in coastal demand centers.

## Results

### Offshore wind resources and economics

We first incorporate wind speed and direction data from NASA’s MERRA2 dataset^25^, a reanalysis product defining the hourly meteorological wind field with a spatial resolution of 0.5 degree latitude by 0.625 degree longitude. Hourly capacity factors for all coastal areas of mainland China over the past 40 years were evaluated at a geographical resolution of about 50 km assuming deployment of typical 8 MW offshore turbines (see SI). Statistical decomposition of wind directions for representative coastal areas are presented in Fig. 1b: offshore locations south of the Yangzi river exhibit dominant winds from the southwest, while winds in northern areas are more evenly distributed. These data were incorporated in a rigorous wind power simulation model (see methods and SI), accounting for wake effects, power transmission losses and optimal layouts for turbines.

The economics of offshore wind power depend on both the quality of wind resources and required capital expenditures. Investment in offshore wind turbine projects is modeled considering turbines, foundations, convergence cables, offshore substations, delivery cables and reactive power compensators (see methods and SI), accounting for variations in water depths and distances to shore. The bathymetric data were derived from GEBCO, with a resolution of 15 arc second^26^. The selection between AC and DC delivery schemes was optimized based on wind project locations (see SI). Resulting levelized costs of electricity (LCOE) for 2030 are presented in Fig. 1a. LCOE off the coast of Fujian can be as low as 0.04USD/kWh (0.27 RMB/kWh), half the cost of nuclear power^31^, 40% lower than the benchmark cost for a coal-fired unit^13^. Significant coastal areas off Zhejiang, Jiangsu and Shandong could also be developed with LCOE around 0.06 USD/kWh (0.4 RMB/kWh), competitive with local coal-fired units.

**Fig. 2:**
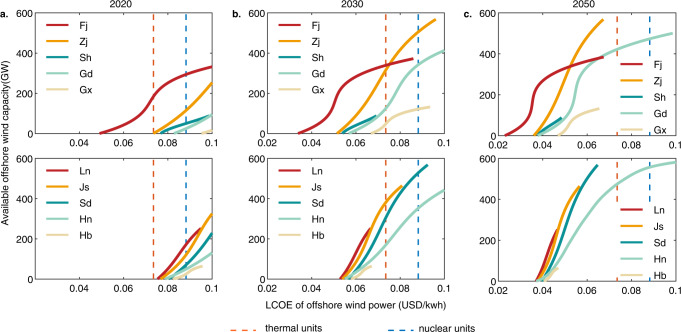
Offshore wind supply curves for all the coastal provinces at different targeting years. Provincial supply curves for 2020, 2030 and 2050 are indicated in **a**, **b**, and **c** respectively. Cumulative offshore wind capacity available at each LCOE level, with provinces differentiated by line color. Levelized cost for benchmark coal-fire and nuclear power units equal 0.07 and 0.085 USD/kWh respectively, presented in blue and orange dashed lines.

Supply curves (maximum available wind capacity at different LCOE levels) for offshore wind for all the coastal provinces are presented for 2020, 2030, and 2050 in Fig. 2. Ocean areas with unsuitable characteristics (harbor areas, shipping routes, and environmental protection zones for example, see SI) were excluded in deriving these supply curve results. In 2020, only 200 GW of offshore wind capacity in Fujian could be supplied with a LCOE below the benchmark for coal-fired units. When compared with the prices for nuclear alternatives, 1000 GW of offshore capacity could be available competitively, mainly in Fujian (300 GW), Liaoning (165 GW), Zhejiang (120 GW), Jiangsu (120 GW) and Shandong (70 GW). Offshore wind power is not yet cost-competitive with nuclear units in Guangdong due to less favorable wind conditions. With a projected 30% decline in LCOE for offshore wind in 2030, about 2000 GW of offshore wind capacity could be installed at a price below coal-fired units, with 3000 GW available at a cost competitive with benchmark nuclear plants. Given the projected decline in LCOE by 2050, nearly all of the offshore wind resources are cost-competitive with benchmark coal-fired units. Offshore wind power is more than sufficient to account for any conceivable long-term increase in power demand in coastal regions, where peak loads amounted in the aggregate to about 560 GW in 2019^32–39^.

### Feasibility for offshore and onshore wind development under the current grid and policy barriers

Given the significant economic potential for offshore wind power in China, grid integration feasibilities are analyzed for all coastal provinces (offshore) and wind rich “three north” provinces (onshore). Hourly power system simulations were conducted throughout a year for all 32 provincial regions in mainland China under all possible wind penetration levels, accounting for all operational characteristics (ramping, minimum on/off times, output range, and fuel costs) for over 3000 thermal units, actual hourly power demand variations and all existing inter-provincial transmissions (see details in methods and SI). Two aspects of grid integration limitations are considered: (1) policy constraints enforced by the NDRC to suspend renewable investments at provincial levels when curtailment rates exceed designated thresholds; (2) the economic feasibility of wind investments accounting for both local wind conditions and curtailment.

**Fig. 3:**
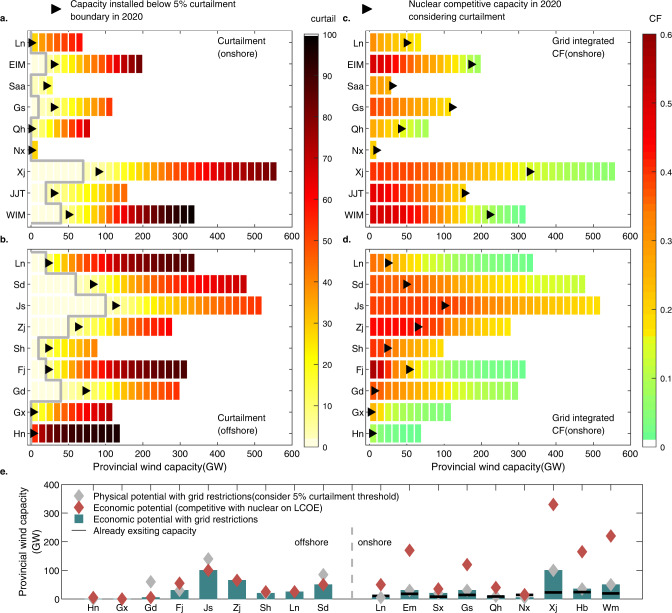
Annual curtailment rate and realized capacity factor for all the coastal provinces under various wind investment levels. **a** Annual onshore wind curtailment rate in 2020 grid structure. Limitation for the 5% curtailment rate proposed by NDRC is presented with black triangles. **b** Annual offshore wind curtailment rate in 2020 grid structure. Limitation for the 5% curtailment rate proposed by NDRC is presented with black triangles. **c** Realized capacity factor of onshore wind power considering possible curtailment losses in the integration simulations. Capacity cost-competitive with nuclear power on LCOE basis in 2020 is presented with black triangles. **d** Realized capacity factor of offshore wind power considering possible curtailment losses in the integration simulations. Capacity cost-competitive with nuclear power on LCOE basis in 2020 is presented with black triangles. **e** Provincial distribution of the economic potential competitive with nuclear power on LCOE basis in 2020 (red diamond), and the physical potential with grid restrictions considering 20% curtailment threshold in 2020 (gray diamond). Economic potential considering both LCOE and integration barriers is presented with green bars.

Curtailment rates for different provinces at all possible wind penetrations are summarized in Fig. 3a (onshore) and Fig. 3b (offshore) respectively. Onshore wind curtailments for inland northern provinces rise rapidly given the limited local power demand, whereas curtailment rates for offshore facilities in major coastal provinces (including Guangdong, Jiangsu, and Shandong) are only apparent when wind investments exceed 100 GW. When applying the policy constraints on curtailment thresholds enforced by the NDRC (15% for Xinjiang and Gansu, 8% for WIM and 5% for other provinces), wind power potential (indicated by black dots in Fig. 3a, b) decreased significantly for essentially all inland provinces. Offshore wind, in contrast, is less affected except for Fujian and Hainan.

The economic potential of wind power, indicated by grid integrated capacity factors (considering both local wind resources and curtailments) is summarized in Fig. 3c (onshore) and in Fig. 3d (offshore). The economics for onshore wind, despite facing higher curtailment rates, is still more favorable in the “three north” region. LOCE for onshore wind in EIM, WIM, JJT and Xinjiang could still reach 0.25 CNY/kWh at 50 GW of wind installation after accounting for the curtailment loss. The economic potential for offshore wind power, when accounting for both resources and grid integration, could also reach 50 GW in major coastal provinces such as Fujian, Jiangsu, Zhejiang and Shandong.

Factors limiting the development of offshore wind power in all coastal provinces and onshore wind bases in the “three North” area major inland wind bases are quantified in Fig. 3e. Abundant physical resources are conveniently available for both onshore and offshore wind power. Onshore wind is generally more favored economically, but faces more severe grid integration barriers due to low local power demand and limited inter-regional transmission capabilities. In contrast, offshore wind, despite being less economic than onshore counterparts, has becoming increasingly cost-competitive and could access to load centers more conveniently. Overall, realizable offshore wind potential exceeds 300 GW under 2020 cost levels and grid conditions, and far exceeds the current government planning level, calling for higher rates of deployment. Onshore wind potential is heavily restricted by the curtailment threshold despite better economics, calling attention to the need for relaxation of government regulations and for strengthening of transmission systems.

### Optimal deployment plan of offshore, onshore wind and solar power in 2030

The Chinese government, along with its ambitious commitment to carbon neutrality by 2060, announced a mid-term target to reach 1200 GW of non-hydro renewable investments by 2030, targeted to provide 40% of electricity demand using the contribution from renewables. In response to this national policy, provincial governments released investment agendas for onshore, offshore wind and solar PV for 2025 and 2030 (refer to Table S23 and figure S33 for details). These investment targets amount to a total of 1228 GW by Feb 2022, in line with the national objective. The provincial plans are aggregated and defined as “provincial government strategies” hereafter.

To test the effectiveness of current provincial government strategies and to search for improved renewable investment portfolios for different provinces, a system planning model is introduced, and used to identify the investment decisions of the newly-built generation capacities of non-hydro renewables (onshore, offshore wind and solar PV), thermal units and different types of storage systems for each province by 2030. Interprovincial transmission facilities account for existing and recently approved or government planned transmission corridors, following assumptions of rigid DC transmission network operation. We did not allow for additional transmission expansion in the 2030 timeframe as approval and construction of interprovincial transmission projects require longer time periods. Deployment for storage systems accounts for lithium batteries and pumped hydro. Hourly simulations of all of the generators, inter-provincial transmissions and storage systems in all provinces for a full year are embedded in the investment model to better reflect the curtailment issue. A full set of flexibility constraints for thermal units, operation properties for hydro power stations, and the practical restrictions for CHP plants are incorporated in the “system integration model” (see details in SI and methods).

**Fig. 4:**
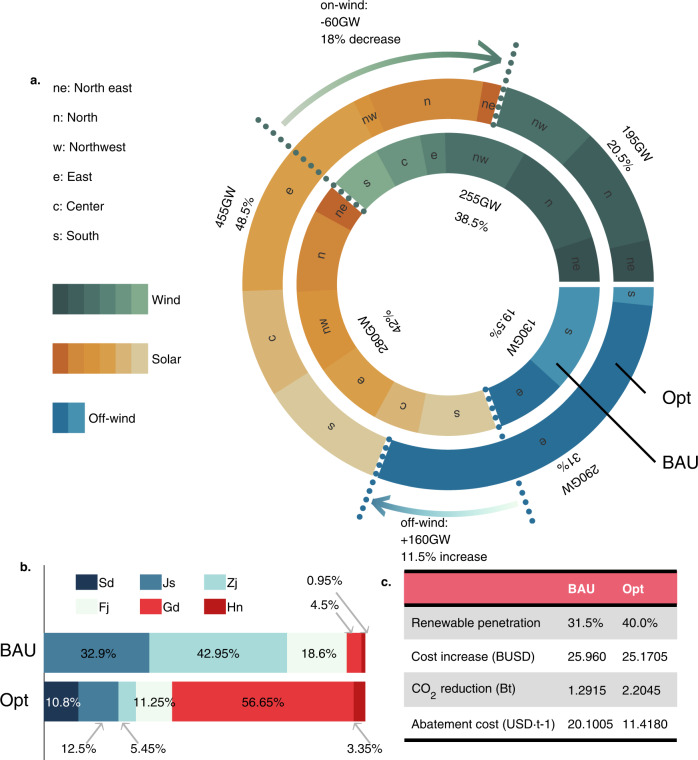
Regional distribution of the major renewable alternatives in National planning and our Optimization results. **a** Regional distribution of the onshore wind, solar and offshore wind power in the National planning (inner circle) and our optimization planning (outer circle), with each renewable alternative characterized by color types (summarized in left legend). Renewable allocation in each region is calibrated with color depth and regional abbreviation (also summarized in left legend). **b** The renewable penetration (including hydro power), cost increase and carbon avoided compared with the baseline scenario. The carbon dioxide abatement cost (expenditure to avoid 1ton of CO_2_). **c** Offshore wind provincial allocation in the national planning and our optimization results. Province in the South Grid is calibrated with red color and province in East Grid is calibrated with blue color.

Two scenarios are considered in our simulation: (1) Business As Usual (BAU) strategy fixes the capacity investment for non-hydro renewables according to the provincial government plan, while allowing for free expansion of thermal units and storage systems; (2) optimal planning strategy (Opt) optimizes the provincial investments for all of the non-hydro renewables, thermal units and storage systems, to fulfill the 40% renewable penetration target proposed by NEA. We note that the government plan in the “BAU” scenario is defined up to Feb 1st 2022 reflecting the status of 14^th^ Five Year Plan, and later changes in government plans are not reflected. The simulation results for the “BAU” and “Opt” scenarios are presented in Fig. 4a.

The BAU strategy emphasizes the investment in onshore wind (340 GW, 41.2%) and solar PV (355 GW, 43%), supplemented by offshore wind (130 GW, 15.8%). Lacking national level coordination, this bottom-up strategy leads to misplacement of a significant portion of renewable investments to regions with inferior renewable resources and/or higher costs. For the onshore wind deployment, Central and South China, with capacity factors as low as 0.17 (compared with 0.46 in Inner Mongolia), are allocated 70 GW of onshore wind expansion, accounting for 30% of the total onshore wind investment. For the offshore wind assignment, over half of the quota is allocated to Guangdong, which is generally identified with a levelized cost 50% higher than that for neighboring Fujian province (indicated in Fig. 2a).

Our optimized planning model indicates an improved renewable portfolio of 195 GW onshore wind, 290 GW offshore wind and 455 GW solar PV. Investment in offshore wind increases from 130 GW to 290 GW, accounting for 30% of the renewable portfolio, whereas the share of onshore wind shrinks from 38.5% to 20.5%. The onshore wind investments in Central and South China with inferior wind conditions are replaced with offshore wind in East China. Deployments of solar PV are transitioned from the centralized investment in the “Three North” regions to distributed development in Central and Coastal load centers. The optimized renewable investment strategy greatly elevates the realized capacity factor for onshore wind (from 22.85% to 29.35%) and for offshore wind (from 33.85% to 40.10%), while the capacity factor for solar PV remains relatively unchanged (from 20.10% to 19.65%).

The provincial allocation of offshore wind capacity is improved also from the BAU strategy. Close to half of the offshore capacity in the BAU strategy is allocated to Guangdong, where offshore resources are generally inferior. While Fujian and Zhejiang, with averaged capacity factors 40% higher than Guangdong, contribute only 15% to the national offshore quota. Our optimized results prioritize the deployment in (1) Zhejiang and Jiangsu, taking advantage of their superior offshore wind resources and adequate grid integration capabilities; and (2) in Fujian, the best offshore resources in China. Our optimized allocation elevates the average offshore wind capacity factor from 0.3385 to 0.4010, by over 20%, while significantly lowering the curtailment rate.

Overall, the BAU strategy for 1228 GW of renewable installations contributes only to a penetration of 31.5% by 2030 (including renewables generated from existing hydro power), falling short of the 40% projected target. Our optimized investment plan elevates the renewable penetration level from 31.5% to 40%, with a lower system cost. Also, the improved renewable allocation could contribute to a reduction over the lifespan of these renewable projects of an additional 20 billion tons of CO_2_ emissions compared with the current BAU strategy, equivalent to 60% of global CO_2_ emissions in 2020.

### Implications of offshore wind deployment on generation mix, inter-provincial transmission and storage portfolios as contributors to carbon-neutrality in China

To explore the role of offshore wind power for deep-decarbonized power systems on a longer time framework, the optimal deployment plans are identified for different generation technologies, inter-provincial transmission and storage systems for 32 provincial regions designed to reach 80% of renewable penetration for mainland China by 2050. The investment model incorporates further the expansion of inter-provincial transmission systems, the deployment of long-term storage systems and options for power-to-hydrogen. Over 600 AC/DC transmission options between different provinces at different voltage levels are identified as technically feasible and included in the optimization model (see details in SI). Long-term storage options, including PHS and compressed air systems, are modeled explicitly accounting for the technological, economical and geographical feasibility. Deployment of compressed air storage considers the properties of available caverns in each province, including their geographical distribution, with estimates for availability of volume and mining activity. Deployment of different P2G technologies and projected provincial hydrogen demand (see SI for details) are also incorporated in this study.

**Fig. 5:**
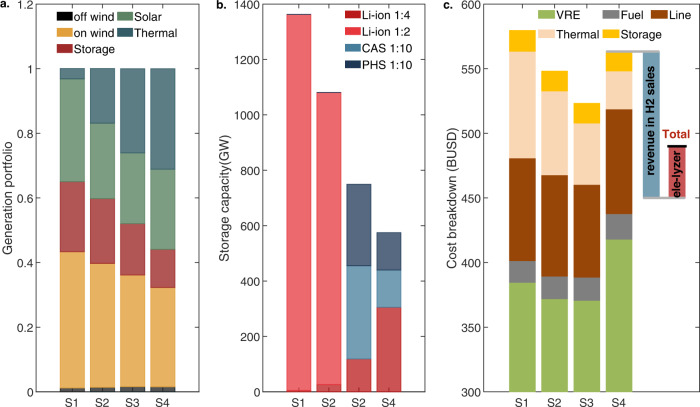
Generation portfolios, storage deployment and system costs under different offshore wind development scenarios. **a** Proportion of capacity increment for all the non-hydro renewables, storage systems and thermal units in above four scenarios. **b** Deployed storage capacity in the proposed scenarios. **c** System cost breakdown in above four scenarios. The capital expenditure of H_2_ electrolyzer and system benefit in hydrogen selling in S4 are illustrated with red and blue bars on the right, respectively.

Based on the above investment model, four scenarios (S1–S4) are considered to evaluate the critical factors influencing offshore wind deployment with the objective of reaching 80% renewable penetration by 2050: **S1** restricts the offshore wind capacity to a level below 200 GW, reflecting the results from previous 2050 pathway studies (refer to the Table S25 for details), and utilizing only lithium batteries as the storage option; **S2-S4** freely optimize the offshore wind deployment, where **S2** considers lithium battery as the only storage alternative; **S3** allows for the deployment of both lithium batteries and long-term storage such as compressed air (CAES) and pumped hydro (PHES), **S4** allows further for the expansion of P2G infrastructures to satisfy the national hydrogen demand. The optimal generation portfolios, deployment scales for different storage technologies, and the breakdown of system costs under different scenarios are illustrated in Fig. 5.

The deployment scale for offshore wind power reaches 920 GW in 2050 in scenario S2, 4.6 times the restricted deployment scale identified in S1, as indicated in Fig. 5a. This largely reflects the changing economics of offshore wind. The overall costs are reduced by $32.5 billion with the increased offshore investment, attributed mainly to the reduced costs for renewables and storage systems. The offshore wind investment scale is boosted further to 1220 GW in S3, when allowing for the deployment of long-term storage options. We noted that lithium batteries are identified as the primary storage option in optimization results for the 2030 40% RPS scenario. However, when the RPS target doubles to 80% in 2050, system balancing beyond diurnal variations is more challenging, and hence the long-term storage options, CAES and PHS, constitute a significant part of the storage portfolio, as indicated in Fig. 5b. Deployment of P2G systems impose an additional demand for power from offshore wind, elevating its installation further to 1520 GW in S4. National hydrogen demand is generally concentrated in coastal regions (accounting for over 60%), mainly due to locally developed automobile and steel industries. Increased deployment of P2G technologies and offshore wind power reduces the overall cost by $33.5 billion/year in S4 compared with the S3 scenario, assuming a benchmark retail price for hydrogen of 2USD per kg.

**Fig. 6:**
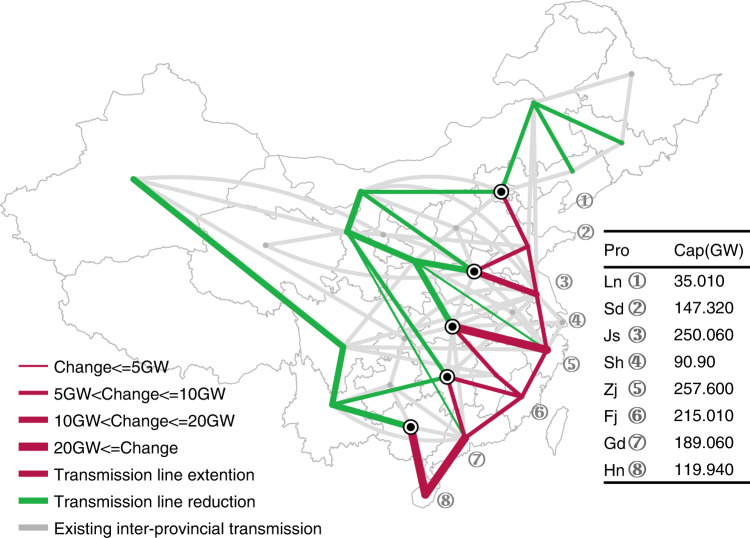
Optimal geographical distribution of UHV transmission investments and offshore wind deployments in mainland China. Capacity variation of transmission network expansion between S1 (limited level of offshore wind according to prier roadmap study), and S3 (freely optimization of offshore wind). All existing inter-provincial transmission lines are presented by gray lines. Red lines illustrate the transmission increment, while green lines illustrate transmission decrement. Provincial allocation of offshore wind investment under S3 is presented on the right table.

With the expanded investment in offshore wind power, the national transmission infrastructure will experience a paradigm shift. The transmission networks for S1 and S3 are compared and illustrated in Fig. 6. The gray and red lines illustrate the optimized interprovincial transmission structure for 2050 in S3. With the large-scale deployment of offshore wind, transmissions in the eastern, coastal regions are strengthened (indicated in red). The strengthened networks are represented mainly by two different categories: a) interconnecting coastal provinces to mitigate offshore wind variability at larger geographical scale, and (b) strengthening connections between coastal and neighboring inland provinces to facilitate the offshore wind accommodation. On the other hand, part of the long-distance, high-voltage DC transmission connections in the S1 scenario could be avoided, as indicated by the green lines in Fig. 6.

## Discussion

This paper develops an integrated offshore wind development plan for China, accounting for the potential for offshore wind resources and economics, grid integration for coastal provinces, and optimal investments in cost efficient planning. High resolution meteorological and bathymetric data as well as the latest technical-economic data for offshore wind power deployments are incorporated in the model, accounting also for data on power system operations, transmission, and consumption. We conclude that China has abundant wind resources and favorable bathymetrical conditions to develop offshore wind power. About 1000 GW of offshore capacity could be available at a levelized cost below that of nuclear power, equivalent to 2.5 times the present average coastal demand for power. However, power system integration and policy restrictions place a major limitation on harnessing renewable resources. Grid integration barriers are more severe for onshore wind power than for their offshore counterparts under current system configurations. Provincial renewable investments in onshore, offshore wind and solar power by 2030 are optimized and compared with the provincial government 14th five-year-plans. The resulting offshore wind investment level could be as high as 290 GW, more than double the current government target. The optimized renewable portfolio with an expanded contribution from offshore wind power elevates the national renewable penetration to 40% (accounting for existing hydro units), an 8.5% increase compared with the 14th provincial government five-year-plans and with lower system costs.

President Xi Jinping announced recently a commitment for China to realize carbon neutrality by 2060^3^. Offshore wind power, the contribution for which has been underrated in almost all prior national roadmap studies, may become one of the most important options for China to realize the goal enunciated by the President. Deployment of offshore wind farms in China by mid-century could not only provide the largest market for the global wind industry in the upcoming decade, but it could offer also an important building block for China to transition away from fossil fuel-based energy systems, providing renewable power and generating green hydrogen.

Given the abundance of the economically available offshore resources, the challenge is how to cost-effectively deploy the investment among provinces, recognizing significant differences in both economics and climate impacts. Currently 130 GW of nationally planned offshore wind capacity is concentrated in Guangdong province (60 GW before 2030)^15^. However, a more even distribution of offshore facilities among FJ, ZJ, JS, and GD provinces could elevate the national averaged offshore capacity factor from 33.9% to 40.1%, with lower total investments. Development of offshore wind farms in China follows a bottom-up strategy: determined by the local government with approval by the central NDRC. A coordinated, carefully studied, top-down government plan is required to facilitate the sustainable integrated development of the offshore wind industry.

Since major onshore wind, solar and hydro resources are all located in northern and western China, the national transmission structure features a west to east, long distance, high voltage paradigm. With aggressive development of offshore wind power, the national energy supply structure should be altered; further strengthening of the grid connection in the eastern provinces is required and would render western-to-eastern UHV lines unnecessary. China announced plans for investment of 5 trillion USD in new infrastructure for the post COVID-19 recovery^40^, and investment in ultra-high voltage transmission systems is one of the seven major areas of emphasis in this plan. As indicated earlier, it is important to coordinate the offshore wind investments with the national UHV upgrade plan.

A significant development of offshore wind power requires changes also in the portfolio of storage systems under elevated levels of renewable penetration. Long-term storage systems will replace lithium batteries and become the primary contributor to the storage portfolio. Long-term storage systems could not only address the long duration fluctuations, but function as a powerful back-up capacity for system reserves. However, recognition of the importance of this capacity is not reflected in pricing mechanisms incorporated in current market operations nor has it been recognized in the most recent initiatives for power market reform. China is transitioning now to open the world’s largest national electricity market—approximately $500 billion trading volume annually. The current emphasis is on designing functional wholesale markets. We note here that introducing proper capacity payment strategies reflecting the contribution of wind power and storage systems will be vital for the ultimate economically driven replacement of coal-fired units.

## Methods

### Numerical simulation and cost estimation for offshore wind farms

The detailed analytical model for estimating the cost of offshore wind projects for all possible locations in China is developed here considering mainly techno-economic characteristics of three subsystems: turbines, foundations and transmission systems. Numerical simulation for offshore wind farm potential is conducted to account for the wake effect of turbine interference and transmission loss for AC/DC systems.

To quantify turbine costs, contract prices were collected for all the offshore wind projects in China during 2019 (further presented in SI), and we adopted the average contract prices on a per megawatt basis in our analyses. For each possible coastal location, construction costs for three turbine foundations (monopile and jacket for fixed bases, as well as the semi-submersible for floating bases) were quantified based on rigorous calculations (see SI), reflecting variations in ocean depth and turbine rating^28^. The optimal foundation types and associated costs were determined then for all potential locations. For submarine power delivery, costs for three mainstream transmission options (33 kV AC, 220 kV AC and ±300 kV DC) were identified for each coastal location. Optimal transmission types and corresponding costs were specified then based on the distance to shore. The optimized foundation and transmission solutions for all coastal locations are summarized in Fig S4.

For all potential wind farm locations off the coast of mainland China (sized 0.5° × 0.625°), typical turbine layout and cable routings were optimized to minimize the capital expenditure while maximizing overall efficiency. Turbine layout was optimized by minimizing total turbine/turbine interference, with wake effect as the main consideration. To facilitate wake effect evaluation, data aggregation was employed to integrate hourly wind information covering the past 5 years and generate typical wind conditions for the wake effect calculations. Our wake effect model reveals axial wake trend with regular Jensen model^41^, and regulates the radial wake trend following the rule of Gaussian distribution. Coefficients of this distribution are further obtained through the parameter fitting of abundant real-world wind field data. The effectiveness of this model has been demonstrated in field surveys^42–45^. The routing for convergence cables is optimized by minimizing total cable length, which accounts for the horizontal turbine layout as well as the vertical cable curvature related to turbine foundation types (see details in SI). Power losses for the overall system consider wake effects, substation, and delivery impacts, as well as other miscellaneous losses (such as icing and blade soiling, abnormal temperature shutdown and lightning shutdown). For AC delivery systems, location and capacity for shunt reactors are also optimized to minimize power losses. Derived typical wake efficiencies for all the grids on the Southeast coast are employed in supplied curves and subsequent investment models. Detailed mathematical formulation of the proposed model is described further in the SI.

### Meteorological and Bathymetry Data for Offshore Wind Analyses

We integrate multiple high-resolution datasets to form a multi-layer data network incorporating (a) physical resource data–wind speed and ocean depth (b) spatial filtering data–exclusive economic zones (EEZ), shipping lanes and maritime reserves and (c) technical-economic data – latest offshore wind capital expenditure.

We derive offshore wind data from NASA’s MERRA2 dataset, a reanalysis product defining hourly wind speeds with a spatial resolution of 0.5 degree latitude by 0.625 degree longitude from 1980 to present^25^. Turbine power output is calculated on an hourly basis using the typical power curve of the V164-8MW offshore turbine (powered by MHI-Vestas). Ocean depth data are taken from GEBCO One Minute Grid, a global bathymetry grid providing information at 1-arc min resolution^26^. The MERRA2 grid is rescaled to the high-resolution GEBCO grid for full utilization of these two datasets.

To determine ocean locations suitable for offshore wind installation in China, we filtered data spatially based on a number of criteria. Locations considered here for offshore wind construction are limited to lie within China’s EEZ. The EEZ is defined as the region under which a coastal state assumes jurisdiction over marine resources, and is derived from Marine Regions^46^. Areas designated as either Special Maritime Reserves or shipping lanes are excluded. Special Maritime Reserves are calibrated by National Maritime Data^47^, while shipping lanes are estimated using SO2 emission data derived from the MERRA-2 dataset as a surrogate identification^25^: 20% of the cell area is removed for locations defined as emitting SO2 at a rate higher than 10  ^−11 ^kg m ^− 2 ^s^ − 1^.

### Power system simulation model for onshore and offshore wind integration

The economics of wind power investment is determined by both the quality of local wind resources and the discount imposed by the system accommodation capability. Power system modeling is conducted on an hourly basis throughout a year, simulating and optimizing system operation. The potential curtailment rate at every wind investment level for onshore wind in the “Three North” regions and offshore wind in the coastal region is obtained from the simulation results.

The unit commitment model is employed in the simulation to balance the variation of provincial power demand with an hourly resolution. The objective is to minimize system operation costs, including start-up costs and fuel costs for thermal units, fuel costs for nuclear plants, and maintenance costs for each generation category. All the flexibility constraints are incorporated for thermal units, including unit ramping limits, minimum online-offline times, minimal output levels and must run constraints. The inter-provincial AC corridor is freely dispatched on an hourly basis, allowing for bi-directional power flows for the connected provinces. The DC corridor is dispatched adopting a fixed strategy and allows only for a uni-directional power flow. Its utilization rate equals 90% during the daytime and 50% throughout the night. We noted that the above constraints are relatively conservative and the operational flexibility can be improved for DC transmissions. Hydro power is modeled considering natural inflows, reservoir storage and various controlling options^48,49^, participating in both power balance and system backup. Reserve constraints and security constraints are included for all the provincial regions.

For an accurate description of power system operations, high-precision generation, consumption and transmission datasets are employed in the power system simulation model. For the generation sector, detailed operational parameters for over 3000 thermal units are incorporated nationally. Associated fuel costs are determined on a provincial basis, according to costs for locally supplied fossil-fuels. Natural water inflows are employed for all major hydropower stations. Related head heights are evaluated by associating the water inflow over the last five years with corresponding power generation during the same period^49^. For the consumption sector, real-world provincial load demand is provided by the State Grid Corporation of China on an hourly basis for the entire year. The heating period for Northeast China, North China and Northwest China is also identified on a provincial basis. For the transmission sector, parameters are incorporated for all of the existing and recently planned inter-provincial transmissions, including the connected provincial regions, capacity ranges and line types.

Fast unit commitment is employed to improve the computational efficiency in recognition of the large scale of this analysis. Instead of scheduling the committed and dispatched behavior for thousands of individual generators at each time interval, thermal units in each provincial region are aggregated according to fuel type, nameplate capacity, and as to whether they involve CHP units or not. Installed capacity, online capacity and actual power output within each thermal group are optimized for different time intervals. All chronological constraints are included and formulated equivalently as compared with the regular UC model. This method is shown to be more than 20,000 times faster than the rigorous UC model, with less than 1% deviation in simulation results^50^. Mathematical formulation of the proposed model is detailed further in the SI.

### Integrated Investment Model for All of the Non-hydro Renewables, Thermal Units, Transmission and Storage

The optimal investment for all the non-hydro renewables (onshore, offshore wind and solar PV), thermal units, inter-provincial transmissions and storage systems is optimized in this investment model. This model determines in a single optimization both the investment decisions and system operations. Investment decisions account for capacity expansions and geographical allocations for all the non-hydro renewables, thermal units, inter-provincial transmissions and storage systems. Operational decisions account for the committed and dispatched status for all the generation categories over the 8760-hour operation of these systems. Capacity available during dispatch is linked directly with the investment decisions.

The objective of the investment model is to minimize the overall system cost, including (1) system operational costs: start-up costs and operation costs for thermal units, fuel costs for nuclear plants, fixed O&M expenses for each generation category; and (2) system investment costs: amortized capital expenditure for newly deployed non-hydro renewables, thermal units, transmissions and storage systems. Investment costs for offshore wind are determined on a provincial basis using the cost estimation model indicated above, also with respect to local investment levels and provincial physical potentials.

This integrated investment model considers a full set of constraints for system operations. Besides the constraints mentioned previously in the power system simulation model, investment and operation characteristics for newly installed transmissions and storage systems are also specified. Over 600 AC/DC transmission options between different provinces at different voltage levels are identified as technically feasible and included in the optimization model. Related investment costs on a per megawatt basis are obtained by the parameter fitting of real-world project data^51^, with spatial distance and engineering difficulty as the main considerations. Storage systems account for all of the mainstream technologies (short-term storage such as lithium batteries, and long-term options such as pumped hydro and compressed air). Long-term storage options are modeled explicitly accounting for their technological, economical and geographical feasibility. Deployment of compressed air energy storage considers the properties of available caverns in each province, including their geographical distribution, volume and mining activity estimations. Investment for the storage devices is divided into energy and power items, incorporating regional limitations, annual throughput and life cycle for typical storage systems. Functions in power balance and system standby are both considered for the storage systems.

The future hydrogen economy is also considered in our research. Existing hydrogen generation amounts to 33Mton in 2021, mainly sourced from fossil fuel and employed as the basis material in chemical sectors^52^. Methanol, ammonia and refining industries account for over 75% of the existing demand^53^. Hydrogen demand is projected to increase to 60Mton in 2050, mainly attributed to its applications in heavy-duty transportation and steel production^54^. Hence, future national hydrogen demand increment is divided into each province proportional to the road freight transportation and the steel production (refer to SI for detail). Coastal, near coastal and the inland provinces account for 65%, 20% and 15% of the hydrogen demand, respectively.

For the production side, P2G infrastructures are deployed for each provincial region, including different technologies as AEC, SOEC and PEM. Technical parameters such as conversion efficiency, minimum load and output pressure are incorporated for different technologies. Minimum on-off times and ramping rates are not considered given the superior flexibility of mainstream electrolyzers.

For the transportation side, this study considers: (1) gaseous hydrogen: hydrogen is pressurized and transported through underground pipeline; (2) liquid hydrogen: hydrogen is liquefied and delivered by truck; and (3) organic carriers: hydrogen is transformed into a chemical carrier, transported by trunk and de-hydrogenized at the destination. Chemical carriers include NH3 (Ammonia), DBT (Di-benzyl toluene) and TOL (toluene). For any two provincial regions, cost for the above three transportation modes is pre-calculated on a per kilogram basis. Transportation with the lowest cost is fixed, and the hydrogen transportation cost is calculated as hydrogen transportation amount multiplied by the unit transportation price in our model (refer to SI for details).

### Cost Data Adopted

Offshore wind capital expenditure is modeled as noted in the “Numerical Simulation and Cost Estimation for Offshore Wind Farms” subsection, provincially varying from 2255 to 2870 $**/**kW in 2020, adopting a 30% cost reduction for 2030, and a 49% cost reduction for 2050^55,56^, accounting for learning curves and improved efficiency. The capital expenditure for onshore wind and solar PV is evaluated on a provincial basis according to the Manual of Renewable Energy Data 2015 released by the National Energy Administration^57^. Cost for onshore wind is estimated assuming a 27% cost reduction by 2030 with a 37% cost reduction by 2050^55^. The cost for solar PV is derived from the Annual Technology Baseline available from NREL^58^.

Capital expenditure for storage systems is divided into energy and power investment items. The energy investment item determines the overall storage capacity, while the power investment determines the peak power input**-**output. The cost for each storage option in both 2030 and 2050 is obtained using the estimates for energy storage Technology Data published by the Danish Energy Agency^59^.

### Reporting summary

Further information on research design is available in the Nature Portfolio Reporting Summary linked to this article.

## Supplementary information

Supplementary Information

Reporting Summary

## Data Availability

The source of public meteorological dataset is provided in the References section. Data and methods for the model generation is basically presented in Supplementary Information. Other dataset used in the study is available from the lead contact on reasonable request.

## Code availability

Code for this study is available from the lead contact on reasonable request.
