# Supplementary material for: Grid integration feasibility and investment planning of offshore wind power under carbon-neutral transition in China
Source: Nat Commun. 2023 Apr 28;14:2447. doi: 10.1038/s41467-023-37536-3 (PMC10141809; doi:10.1038/s41467-023-37536-3)
Supplement: Supplementary file 1 — Supplementary Information [file 41467_2023_37536_MOESM1_ESM.pdf]

Supplementary Information for
Grid Integration Feasibility and Investment Planning of Offshore Wind Power under Carbon-Neutral Transition in China

Xinyang Guo[1,2,†], Xinyu Chen[1,2,†,*], Xia Chen[1], Peter Sherman[2,3], Jinyu Wen[1*], Michael McElroy[2,3,†,*]

1. State Key Laboratory of Advanced Electromagnetic Engineering and Technology, School of Electrical and Electronic Engineering, Huazhong University of Science and Technology, Wuhan 430074, China.

2. Harvard John A. Paulson School of Engineering and Applied Sciences, Harvard University, Cambridge, MA 02138, USA.

3. Department of Earth and Planetary Sciences, Harvard University, Cambridge, MA 02138, USA.

***Correspondence: mbm@seas.harvard.edu, xchen@seas.harvard.edu, jinyu.wen@hust.edu.cn**

† These authors contributed equally to the work.

Table of content

0. Summary 5

1. Offshore wind cost estimation model 6

1.1. Wind turbine cost 7

1.2. Turbine foundation cost 8

1.3. Offshore substation cost 9

1.4. Delivery cable cost 11

1.4.1. Cost for AC delivery 11

1.4.2. Cost for DC delivery 12

1.5. Convergence cable cost 13

1.6. Installation cost and maintenance cost 15

1.7. Offshore wind cost reduction 16

1.8. Cost estimation of developing offshore wind power in southeast China 17

2. Offshore wind Wake effect and Turbine layout model 23

2.1. Wake effect model 23

2.2. Turbine layout model 28

2.2.1. Turbine coding 29

2.2.2. Data aggregation 30

2.2.3. Turbine layout arrangement 31

2.2.4. Convergence cable wiring 32

2.3. Data derivation 34

3. Offshore wind Power system simulation model 37

3.1. Decision variables 38

3.2. Objective function 40

3.3. Flexibility constraints 41

3.3.1. Ramping constraints 41

3.3.2. Minimum on/off time constraints 42

3.3.3. Max-min load constraints 43

3.3.4. Must-run unit constraints 43

3.4. Hydro power model 44
3.4.1. Inflow constraints 44
3.4.2. Water level and discharge rate constraints 45
3.4.3. Hydro reserve constraints 45
3.5. Power balance and reserve constraints 45
3.5.1. Power balance constraints 45
3.5.2. Reserve constraints 46
3.6. Simulation settings 46
4. Optimal investment model for 2030 and 2050 50
4.1. Provincial load demand 50
4.2. Decision variable 51
4.3. Objective function 52
4.3.1. Investment cost 53
4.3.2. Operational cost 53
4.4. Flexibility constraints 54
4.5. Hydropower model 55
4.6. Transmission line model 56
4.7. Energy storage model 58
4.7.1. Compressed air storage system 61
4.7.2. Pumped hydro storage system 65
4.8. Power balance and reserve constraints 66
4.8.1. Power balance constraints 66
4.8.2. Reserve constraints 67
4.9. Settings for hydrogen economy 68
4.9.1. Hydrogen demand 68
4.9.2. Hydrogen generation 70
4.9.3. Hydrogen transportation 71
4.10. Simulation settings 78
4.10.1. Scenario settings in 2030 78
4.10.2. Scenario settings in 2050 81
4.10.3. Simulation results 82

Reference 84

0. Summary

The supplementary information illustrates in detail the model and data employed to develop offshore wind power in the southeast coast China. Section I developed a cost estimation model to evaluate offshore wind capital expenditure on a per kilowatt basis for all coastal locations, mainly considering the turbine, foundation, transmission system and other soft costs. As a verification of our model, the contraction prices of latest committed offshore wind projects in China (a total of 92) are also listed for comparison. Section II proposes a wind farm deployment model to determine the optimal turbine layout and convergence cable wiring, to minimize the turbine interference and installation expenses. For the calculation of overall system losses and provincial physical potential, typical wake efficiency and wind farm power density (turbine capacity per square kilometers) are also evaluated based on our model. Section Ⅲ presents a power system simulation model to quantify the system integration at all possible offshore wind penetration levels for each coastal province, formulating in detail the decision variables, objective function and all system constraints, presenting thoroughly the operation model for thermal units, hydro-station and inter-provincial transmissions. Section IV proposes an integrated investment model, to determine the provincial deployments of all the non-hydro renewables (offshore, onshore wind and solar PV) and thermal units, the expansion of inter-provincial transmission systems, the investments of storage systems and the options for power-to-hydrogen.

1. Offshore wind cost estimation model

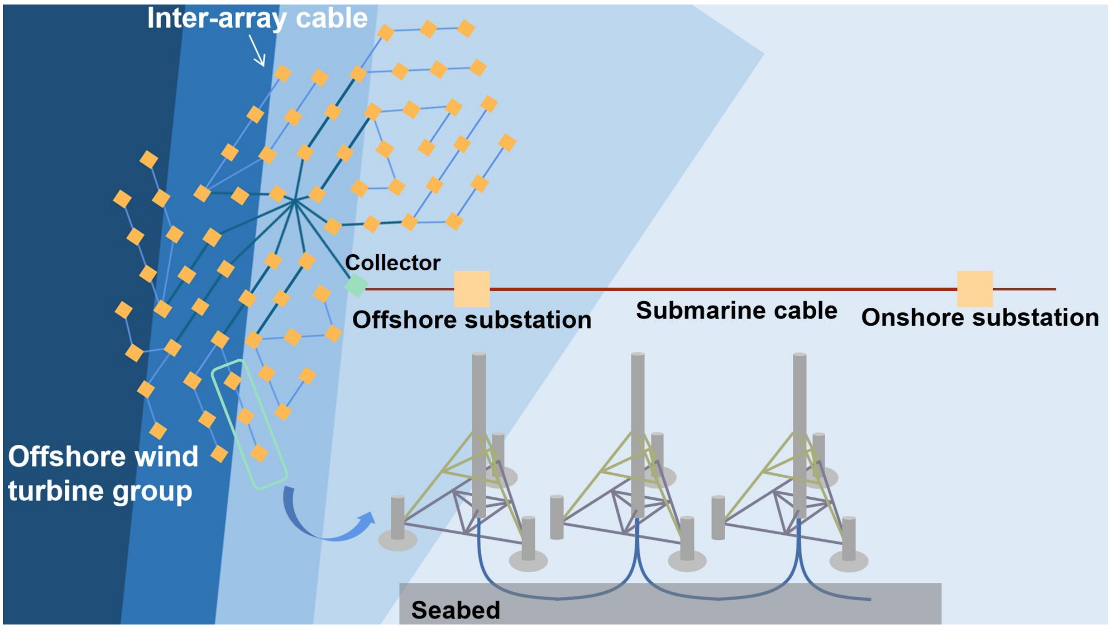

Fig S1: Overall structure of offshore wind projects. Wind power generation is converged using submarine cable strings, gathered at the collector and processed by step-up transformers. Power is delivered to shore through a submarine cable and grid committed after voltage step-down.

Offshore wind overall structure is presented in Fig S1. The investment cost of offshore wind projects is modeled considering six parts, including wind turbine, foundation, convergence cable, offshore substation, delivery cable, and the soft cost represented as installation and operation and maintenance costs. Turbine cost is derived from the contract price on a per kilowatt basis for turbines in all major offshore wind projects during 2019 (in Table S1). Foundation cost is evaluated considering mainstream options (monopile and jacket for fixed based technology, semi-submersible for floating based technology) and quantified based on parameter fitting of practical offshore wind engineering. Convergence cable routing is optimized by minimizing the total cable length, accounting for the horizontal turbine layout and vertical cable bending, as well as the influence of turbine foundation types. The offshore substation is modeled considering electrical devices and supporting base, divided into AC and DC options. Submarine delivery incorporates the 33kV AC, 220kV AC and ±300kV DC options, also considering the reactive power

compensation for AC option. All the soft costs are modeled as a fixed proportion of the capital expenditure.

1.1. Wind turbine cost

The cost for offshore wind turbines is much lower in China as compared with Europe, mainly due to lower commodity prices and labor costs. Turbine contract prices for all the offshore wind projects in China during 2019 are presented in Table S1[1]. The turbine cost used in this study is taken as 870USD per kilowatt, representing the average among the year 2019 offshore wind projects in China.

Table S1: Bid prices for all the offshore turbines contracted in China in 2019[1].

Project	Province	Capacity (MW)	Unit price ($\$\cdot kW^{-1}$)
Guangdong power Zhuhai Jinwan	Guangdong	303	934
Zheneng Jiaxing No.1	Zhejiang	136	--
State Power Investment Jinghai, Jieyang	Guangdong	150	775
State power investment Shenquan 1	Guangdong	200	775
Rudong H5#	Jiangsu	300	794
Funeng Pinghai Bay, Putian, Fujian	Fujian	210	--
State power investment Shenquan 1	Guangdong	200	662
Guohua bamboo root sand H1#	Jiangsu	200	--
Liuao, Zhangpu, Fujian1	Fujian	202	841
Liuao, Zhangpu, Fujian2	Fujian	100	804
Liuao, Zhangpu, Fujian3	Fujian	100	850
Huaneng Guanyun	Jiangsu	400	783
Huaneng Jiangsu Dafeng	Jiangsu	100	1020
Zhongguang nuclear power Shanwei Houhu	Guangdong	500	807
CGN Shanwei Jiazi 1 and 2	Guangdong	900	795
Datang Nanao lemen I	Guangdong	400	893
Huaneng Shantou lemen 2	Guangdong	406	833
Puti Island leting, Tangshan, Hebei Province	Hebei	300	--
Pinghai Bay, Putian1	Fujian	102	886
Pinghai Bay, Putian2	Fujian	210	923
Huaneng Jiaxing No.2	Zhejiang	400	740
Three Gorges Yangxi Shaba phase II1	Guangdong	200	953
Three Gorges Yangxi Shaba phase II2	Guangdong	200	976
Three Gorges Rudong H6#	Jiangsu	400	819
Three Gorges Rudong H10#	Jiangsu	400	819
Changle Waihai	Fujian	300	817

Huaneng Shantou Haimen	Guangdong	550	977
Qidong H1#	Jiangsu	250	1058
Qidong H2#	Jiangsu	250	1058
Qidong H3#	Jiangsu	300	--
China energy Yangjiang NANPENG Island	Guangdong	300	969
Guangdong Shantou Nanao Yangdong	Guangdong	300	887
Yancheng Guoneng Dafeng H5#	Jiangsu	200	947
Zheneng Shengsi 2#1	Zhejiang	200	852
Zheneng Shengsi 2#2	Zhejiang	200	924
--	--	9869	870

1.2. Turbine foundation cost

The capital costs of three mainstream turbine foundations (monopile and jacket for the fixed based technology, semi-submersible for the floating based technology) are quantified based on rigorous mass calculations, reflecting the variation of bathymetry and turbine rating. Mass for all the building blocks in a typical foundation type (presented in Table S2) is evaluated with parameter fitting of the latest committed offshore wind projects[2,3]. The total cost for each foundation type among all the potential area off the coast of mainland China is evaluated, in order to delineate the optimal foundation type and associated capital cost for various application scenarios. The boundary between fixed and floating based foundations are presented in Fig S4.

Table S2: Basic information (bathymetry range, seabed state, overall structure, cost components incorporated in our model) of various offshore wind foundation types.

Sub-Type	Monopile	Jacket	Semi-submersible
Depth	10-60m	10-60m	60-1000m
Ground	Sandy and gravelly composition	Non-rocky ground	N/A
Structure	Hollow cylinders with a diameter about 4m, and a thickness about 5cm.	Multi-chord base formed of multiple sections with pipes or structural tubing.	Multi-legged floating structure with a large deck. All the legs are connected underwater with horizontal buoys.
Cost component	(1) Monopile<br>(2) Transition parts	(1) Main lattice<br>(2) Jacket piles<br>(3) Transition parts	(1) Stiffened column (2)Truss (3)Heave plate(4)Mooring line (5)Submarine anchor

1.3. Offshore substation cost

To minimize power losses during submarine delivery, voltage step-up is conducted by the offshore substation, which consists of electrical facility and supporting base. Electrical facility incorporates AC and DC options, mainly considering main power transformers, switchgears and reactive power compensators (for AC option only). Supporting base incorporates topside structures, foundation platform and bottom piles. Cost for HVDC substation is scaled from the HVAC option, as indicated in[4].

Capital expenditure for electrical devices on AC substation is evaluated considering main power transformer, high voltage switchgear, mid voltage switchgear and reactive power compensator. Nameplate capacity for each main power transformer is designated around 300MVA, with a design margin valued 1.2 reserved as a backup[2].

$$N_{MPT} = \left\lceil \frac{P_{farm}}{300} \right\rceil \tag{1}$$

$$P_{MPT} = 1.2 \times \frac{P_{farm}}{N_{MPT}} \tag{2}$$

Where P_{farm} denotes the nameplate capacity of offshore wind farm, and "⌈⌉" denotes the round up operation. Standard capacity manufactured in the practical engineering is also consulted in the determination of P_{MPT}. The number of both high voltage and mid voltage switchgears is equivalent to that of main power transformers. Mass for all the building blocks of substation supporting base is also estimated with parameter fitting[2,3].

Shunt reactors are employed in AC substation for the reactive power compensation[5]. Three mainstream options are incorporated in our model, including the single ended, double ended and triple ended compensations[6] (presented in Fig S2-a). The deployed location and capacity for each option is optimized to minimize the total compensation cost under all possible wind farm rating and grid committed distance. Related costs for (1) shunt reactors; (2) shunt reactors and revenue losses in submarine delivery are presented in Fig S2-b and Fig S2-c, respectively. Each curve extends to the distance where related compensation option is failed to complete. Based on the overall compensation cost, application scenario of above three options is bordered around

75km and 125km. Notably, although capable for farther delivery distance, triple ended option failed to be employed in the practical engineering given the far shore locations could be addressed even better with HVDC delivery.

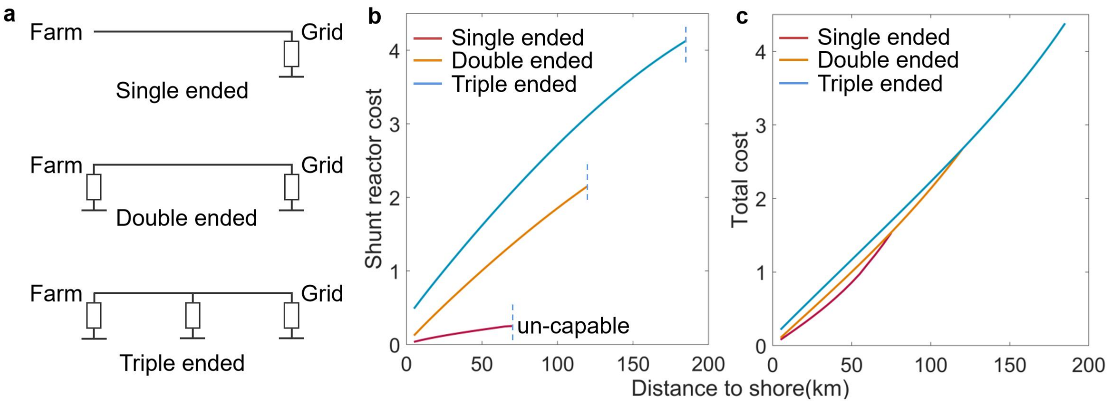

Fig S2: Reactive power compensation for AC submarine delivery. (a) overall structure for the mainstream compensation options (single ended, double ended and triple ended). Farm and Grid denote the offshore wind farm and grid-committed point, respectively. (b) capital expenditure for shunt reactors, including cost increment in offshore substation. (c) overall cost for the reactive power compensation (including not only capital expenditure, but the revenue losses during submarine delivery). Each curve extends to the distance where related compensation option is failed to complete.

Voltage deviation during the submarine delivery is usually insignificant compared with current variation. Adopting the distributed parameter model, current along whole submarine cables under each compensation option is presented in Fig S3. Above three options contribute a minor effect to near-shore locations as indicated in the Fig S3-a, while gradually widen the gap as grid committed distance grows. Compensation option is failed to complete when the maximum current along cable is higher than the cable rating (as the red line in Fig S3-c).

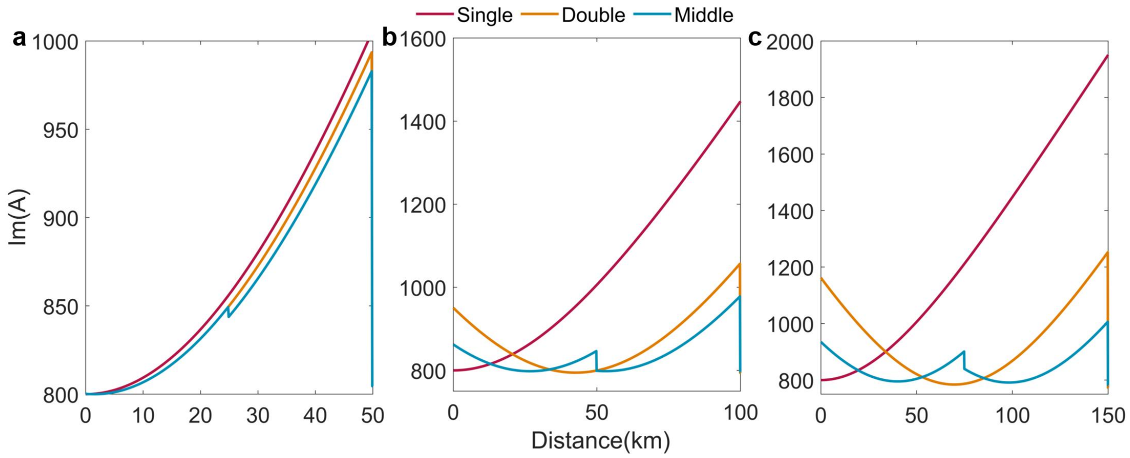

Fig S3: Current along the submarine cable. Current along the submarine cable for three possible delivery distances (a:50km, b:100km, c:150km) are presented. This cable is manufactured at 220kV voltage level and 1100A maximum current rating. Different compensation options are characterized with colored lines.

1.4. Delivery cable cost

Three kinds of submarine delivery are incorporated in our study: including the 33kV AC, 220kV AC and 300kV DC. Costs for each option are modeled as below.

1.4.1. Cost for AC delivery

Cables rated at 33kV and 220kV voltage level are considered in our AC delivery model. The nameplate capacity of each cable is designated considering both transmitted capacity and delivery congestion due to reactive power accumulation, formulated as[7]:

$$P_{rated} = \sqrt{3} \times UI \times \sqrt{1 - \left(\frac{50\pi CUl}{\sqrt{3} \times I}\right)^2} \tag{3}$$

Where C (in F/km) denotes the capacity component along unit cable length. l (in km) denotes the total delivery cable length. U and I correspond to voltage level and current rating for the delivery system. Suppose that the cable selected in submarine delivery is rated at P_{cable}. The number of the delivery cable can be simply formulated as:

$$N_{cable} = \frac{P_{rated}}{P_{cable}} \tag{4}$$

Capital expenditure for the 220kV AC submarine delivery cable is determined with parameter fitting of the projects awarded during 2018 of Oriental Cable company[8], as presented in Table S3.

$$W_{220kV} = 0.00285 \times Dis + 0.065\left(MUSD \cdot MW^{-1}\right) \quad (5)$$

Table S3: Contracted price for the 220kV submarine cables manufactured by the Oriental Cable company during 2018.

Project	Cost	Voltage level (kV)	Distance (km)	Capacity (MW)
No.6 Putuo	8.0	220	11	252
Sanxia Dafeng, Jiangsu	25.3	220	45	300
Pinghai Bay, Putian II	37.9	220	12	250
Shenhua Group	64.6	220	42	300
Huaneng Dafeng, Jiangsu	68.4	220	55	300
Nanri Putian, Fujian	15.7	220	10	400
Yangxi Shaba, Yangjiang	84.3	220	28	300

The cost for the 33kV AC delivery option is formulated as[8]:

$$W_{35kV} = 0.01 \times Dis\left(MUSD \cdot MW^{-1}\right) \quad (6)$$

1.4.2. Cost for DC delivery

Cost model of DC delivery cable is formulated as[9]:

$$N_{cable} = \frac{P_{rated}}{P_{cable}} \quad (7)$$

$$C_{cable_{DC}} = 0.1 \times P_{cable}^{0.5}\left(MUSD \cdot km^{-1}\right) \quad (8)$$

$$W_{AC} = C_{cable-DC} \times N_{cable} \times l \quad (9)$$

Where N_{cable} denotes the number of DC delivery cable, P_{cable} denotes the rated capacity of each delivery cable in MW. C_{cable_DC} denotes the cost of DC delivery cable per kilometer. W_{DC} denotes the total cost for DC delivery.

Boundaries for AC/DC delivery schemes and fixed/floating foundation types are presented in Fig S4 with green and orange lines, respectively. Maritime jurisdictions for each province are presented with colored areas extending to the open sea. 220kV AC delivery still remains the most widely used option off the coast of mainland China, given most of the offshore wind projects are located at shallow water near shore.

Monopile, Jacket and Semi-submersible foundations are employed in turn bordered around 50m and 60m. As a transition to floating based technology, jacket foundation only corresponds to a range of around 10m, which may be attributable to the fact that seafloor soil is not taken into consideration in our cost model.

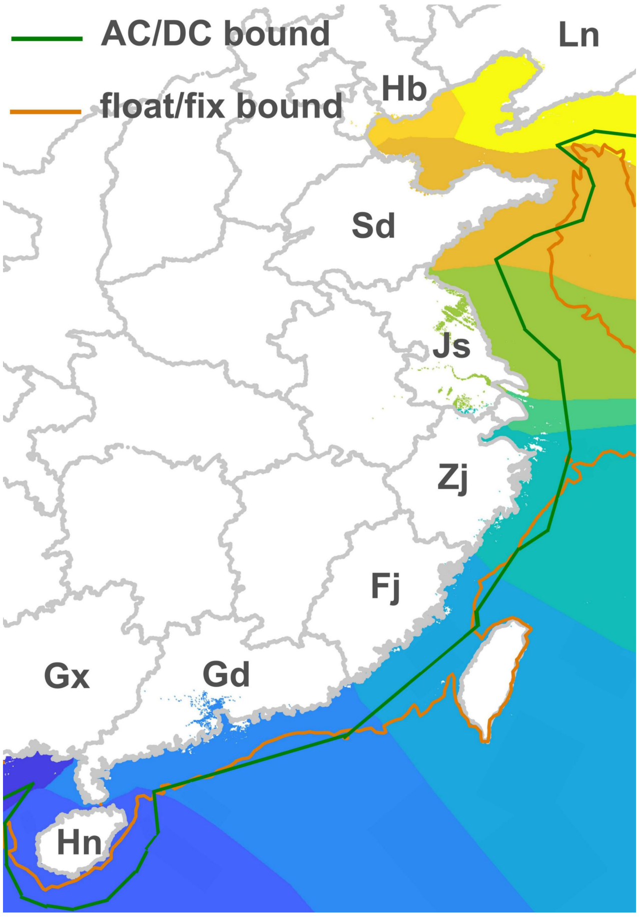

Fig S4: Boundary for each delivery option and foundation type. Boundary for AC and DC delivery options is presented with the green line, while boundary for fixed and floating based foundation types is presented with orange line. Maritime jurisdictions for each province are characterized with colored areas extending to the open sea.

1.5. Convergence cable cost

Convergence cable routing is modeled considering the cable bending, hanging and underwater burial, with horizontal turbine layout and vertical water depth as the main factors. Cable routing schemes are modeled separately for the fixed based and floating based turbine foundations.

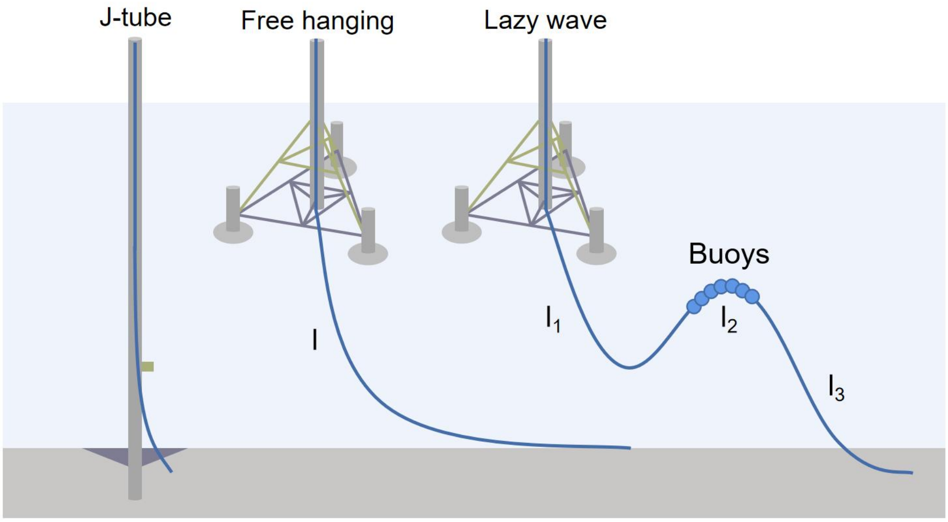

Fig S5: Convergence cable modeling for different foundation types. J-tube: convergence cable for fixed base foundation is modeled stretch into the seabed vertically. Free-hanging: free hanging option for floating base foundation presents L-shape underwater. Lazy wave: lazy wave option for floating based foundation presents S-shape underwater.

For the fixed base foundation, the convergence cable from turbine interference is modeled stretch into the seabed vertically. Cable length between adjacent turbine foundations can be simply formulated as:

$$L = 2(d + D) \tag{10}$$

where d denotes local water depth, D denotes the distance between adjacent turbine foundations.

For the floating base foundation, the convergence cable from turbine interference usually presents a certain shape underwater. Cable hanging is divided into free hanging and lazy wave options (in Fig S5 b-c), with cable breaking load and allowable bending as the main determinations[9,10]. Upper and lower bounds for total cable length is restricted by above two constraints, while the average is taken as the estimation in our model. Having considered the draft associated depth for about 20m, cable length among adjacent turbine foundations can be formulated as[9]:

$$h = d - 20 \tag{11}$$

$$l = 1.05 \times \frac{1}{2} \times \left(1.05 \times \sqrt{5}h + 0.95 \times 3h\right) \tag{12}$$

where $\boldsymbol{l}$ denotes the proposed free hanging cable length, considering a margin valued 1.05 reserved for the cable burial.

Cable length for the lazy wave option is generally divided into (1) double-armored sections at both-ends ($\boldsymbol{l}_1$ and $\boldsymbol{l}_3$ in Fig S5), (2) buoyancy-element section at middle part ($\boldsymbol{l}_2$ in Fig S5). In practical engineering, upper hog bend must stay under hanging- off point, while lower sag bend must keep a clearance for at least 0.1d with seabed. As indicated in dynamic simulation[10], total cable length is estimated about 2.8h, with a length ratio of $\boldsymbol{l}_1$: $\boldsymbol{l}_2$: $\boldsymbol{l}_3$=1: 1: 2. Supposing a horizontal extension for about 2h from the hanging-off point, cable length between adjacent turbine foundations can be formulated as[9]:

$$h = d - 20 \tag{13}$$

$$l = 2.8h \tag{14}$$

$$l_1 : l_2 : l_3 = 1:1:2 \tag{15}$$

$$L = 2l + (d - 4h) \tag{16}$$

Costs for the convergence cable includes both cable budget and burial expenses. Overall costs for the convergence cable engineering are proposed 2M USD/km as a combination of the above two[3].

1.6. Installation cost and maintenance cost

Installation cost includes the assembly and installation cost, port and staging cost, and the logistics and transportation cost. This cost is estimated using fitting formula mentioned in[3].

Operation and maintenance costs for offshore wind farm are largely project dependent, with logistic distance and ocean conditions as the major determinants. Logistic distance denotes the airline distance among the project site and logistic port. Ocean condition is artificially differentiated, with wave height and wind speed as the major criteria. The farther logistic distance and the more severe ocean conditions, the more advanced operation vessel we select and the higher maintenance expenses we afford. Operation and maintenance expenses are modeled as a fixed proportion

multiplied to capital overhead in out model. Previous literatures are surveyed for the offshore wind life-span cost breakdown, with associated O&M proportion presented in Table S4. The O&M factor is estimated as 25% in 2020 as the average results, which falls further to 20% in 2030 considering the faster descent rate for O&M expenses compared with the capital overhead (detailed in Fig S6).

Table S4: Proportions of offshore wind O&M expenses in previous study.

Proportion	Researchers	Year
20.5%	Vega Luis[12]	2018
21%	Tasnim Ibn Faiz[13]	2014
25%	M. Asgarpour et. al.[14]	2014
26%	Shafiee Mahmood et. al.[15]	2016
28%	Sun BoYang et. al.[16]	2016
30%	Athanasios Kolios et. al.[17]	2018
31% or 34%	Tyler Stehly et. al.[18]	2019

1.7. Offshore wind cost reduction

The offshore wind cost reduction projections are derived from the models proposed by NREL in 2019[19]. Reduction for capital expenditure – the cost for offshore wind development per kilowatt basis, is presented with the red line in Fig S6. Offshore wind levelized cost of electricity declines even faster, mainly given improvements in turbine capacity factor and overall system efficiency. Improvements in maintenance technologies also lead to a 40% decline in O&M expenses in the next decade. For the 2030 scenario in our model, we adopt a 30% reduction for capital expenditure and levelized cost, and 40% reduction for the system operation and maintenance cost.

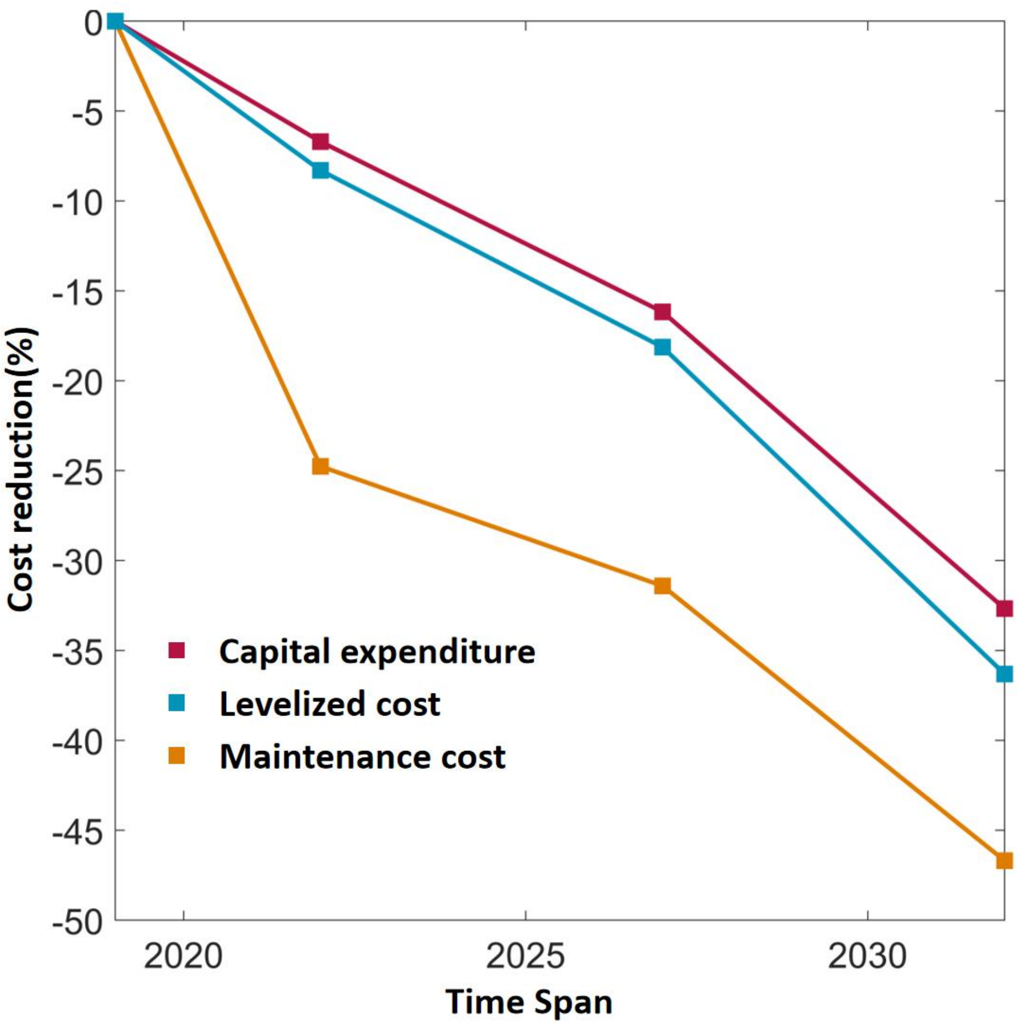

Fig S6: offshore wind cost reduction. With 2019 as the reference year, the percent change in costs for capital expenditure, levelized costs and maintenance costs are extrapolated to 2032[19].

1.8. Cost estimation of developing offshore wind power in southeast China

To estimate the capital expenses for offshore wind development in each provincial region, a cost estimation model is employed for all potential sea area within China's Exclusive Economic Zone. Sea area is divided into fixed grid cells (about 20x20km per cell for 2GW offshore wind potential), with associated capital expenditure and levelized costs calculated as the selection criteria. For a typical provincial region, physical resources are explored for cells with elevated levelized cost. The corresponding procedure is presented in the Fig S7.

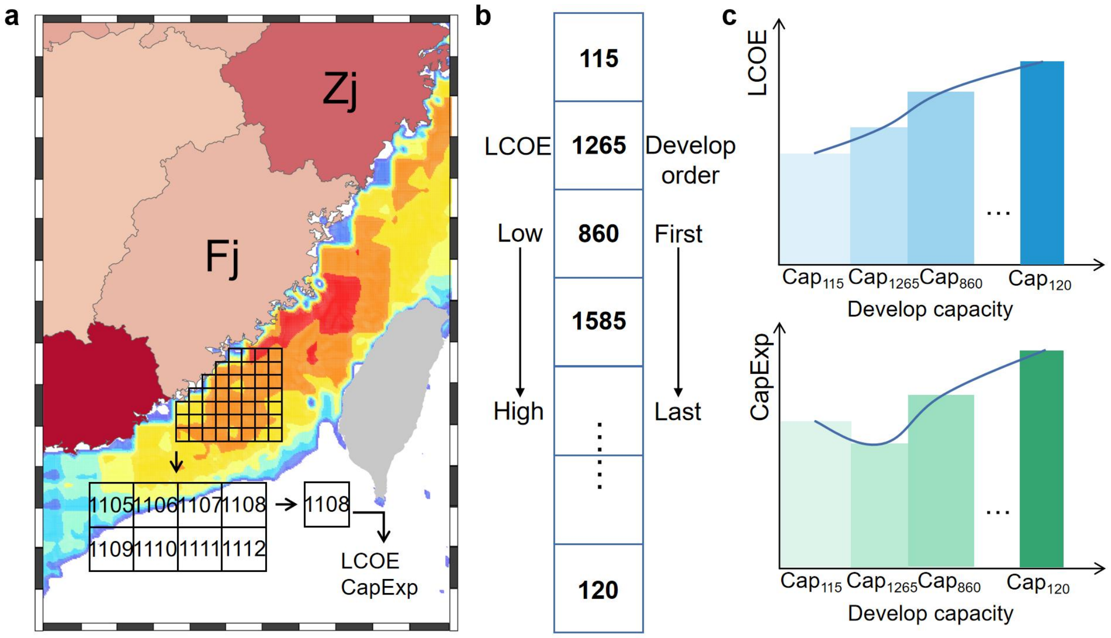

Fig S7: Evaluation of supplied curve and capital expenditure for offshore wind development. (a) sea area off the coast of mainland China is divided into fixed grid cells, with basic information of each cell attached (capacity potential, capital expenditure and levelized cost). (b) offshore wind resource is developed with cell as the minimum unit, according to levelized cost from low to high. (c) For each offshore wind investment level, final levelized cost is evaluated as capacity weighted average of all the selected cells. Capital expenditure is also evaluated on per kilowatt basis of all the selected areas.

All the offshore wind projects contracted during 2018 are collected (see Table S5 for detail) and presented in Fig S8. Provincial costs evaluated by our model are also tagged with green marks (for the first 100GW offshore wind development in each province). The average price obtained by our cost model is basically in the middle of the real-word projects bid prices. The deviation is mainly due to the variation of water depth in different developing sea area.

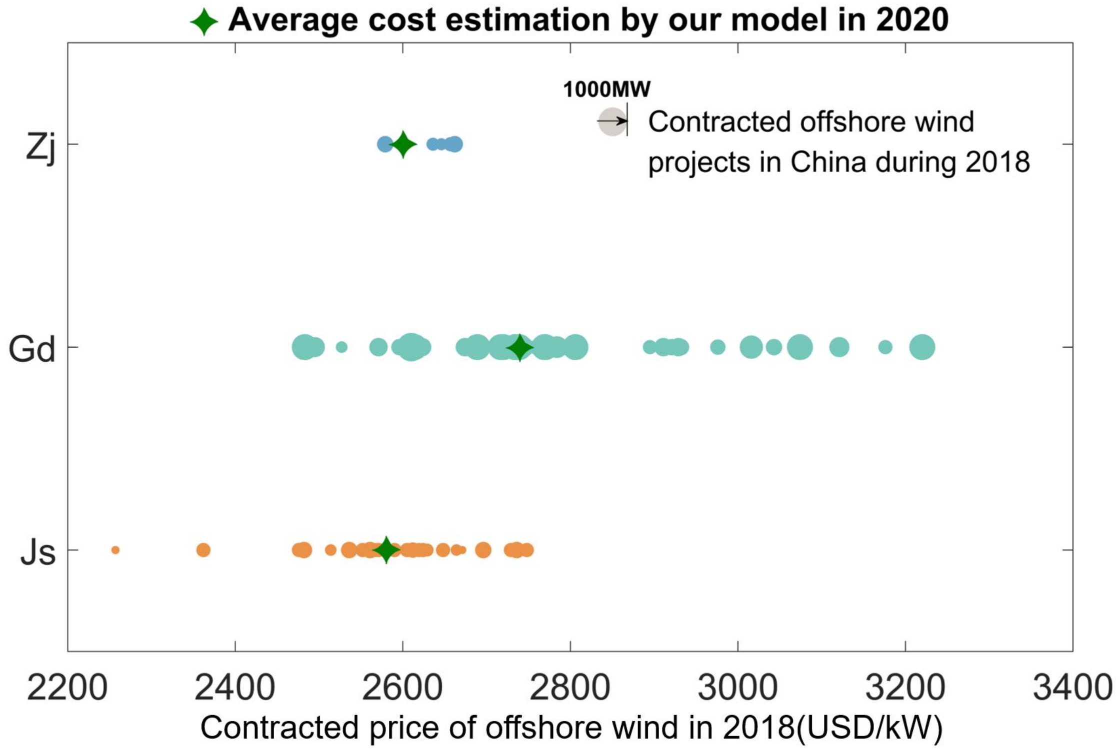

Fig S8: Comparison of offshore wind projects contracted during 2018 and the cost estimation by our model. Each circle denotes the real-world contracted cost of offshore wind projects during 2018 in China.

For the development of the first 100GW offshore wind in Jiangsu and Guangdong, 50 cells are selected in each province according to elevated levelized costs. The two provinces have comparable grid committed distance, while the capital expenditure on a per kilowatt basis is much higher in Guangdong given the greater water depth. Provincial cost breakdowns for Jiangsu and Guangdong are presented in Fig S9.

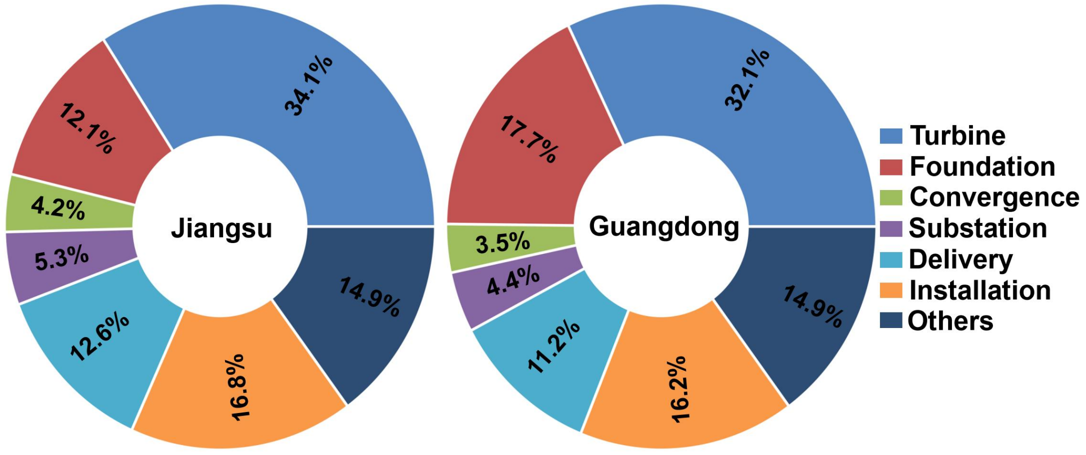

Fig S9: Provincial cost breakdown for offshore wind development in Jiangsu (left) and Guangdong (right). Each cost item is characterized with colored sectors, with associated labels tagged on the right.

Table S5: major offshore wind projects contracted during 2018[20].

	Project Name	Capacity (MW)	Cost($·kW^{-1})
1	Changle offshore wind farm area A	300	3357
2	Putian Pinghaiwan offshore wind farm phase III	312	2839
3	Changle offshore wind farm area C	498	3210
4	Putian Shicheng offshore wind farm project	200	2636
5	Zhangpu Liuao offshore wind farm area D	402	3291
6	Fuqing Haitan Strait offshore wind power project	300	3176
7	Guohua Zhugensha H1 offshore wind farm	200	2571
8	Huaneng Guanyun offshore wind farm	300	2552
9	Rudong H3 offshore wind farm	300	2571
10	Jiangsu Jiangjiasha H2 300MW offshore wind farm	300	2571
11	Sheyang South area H2 300MW offshore wind farm	300	2581
12	GCL Rudong H15 offshore wind farm project	200	2514
13	Jiangsu Zhugensha H2 300MW offshore wind farm	300	2476
14	Jiangsu Binhai South H3 offshore wind project	300	2362
15	Sheyang South H1 300MW offshore wind project	300	2605
16	Sheyang South H2-1 100 MW offshore wind project	100	2671
17	Sheyang South H3 300 MW offshore wind project	300	2671
18	Sheyang South H4 300 MW offshore wind project	300	2738
19	Sheyang South H5 400 MW offshore wind project	400	2748
20	Huaneng Jiangsu Dafeng 100MW wind project	100	2696
21	Longyuan Jiangsu Dafeng H4 300MW wind project	300	2514
22	Longyuan Jiangsu Dafeng H6 300MW wind project	300	2648
23	Yancheng Guoneng Dafeng H5 offshore wind farm	200	2567
24	Jiangsu Dafeng H8-2 offshore wind farm	300	2664
25	Rudong H2 offshore wind power project	350	2590
26	Rudong H3-2 offshore wind power project	100	2612
27	Rudong H4 offshore wind power project	400	2257
28	Rudong H5 offshore wind power project	300	2482
29	Rudong H6 400MW offshore wind power project	400	2624
30	Rudong H7 offshore wind power project	400	2536
31	Rudong H8 300MW offshore wind power project	300	2736
32	Rudong H10 400MW offshore wind power project	400	2729
33	Rudong H13 offshore wind power project	150	2561
34	Rudong H14 offshore wind power project	200	2590

35	Qidong H1 offshore wind farm project	250	2514
36	Qidong H2 offshore wind farm project	250	2629
37	Qidong H3 offshore wind farm project	300	2629
38	CGN Jieyang Huilai 1 offshore wind farm	800	2619
39	CGN Jieyang Huilai 4 offshore wind farm	1000	3016
40	Mingyang Jieyang Huilai Sanhai wind farm project	500	3074
41	Jieyang Huilai 2 offshore wind farm project	500	2674
42	Jieyang Qianzhan I offshore wind farm project	1200	2683
43	Mingyang Jieyang Qianzhan Sanhai project	500	2610
44	CGN Jieyang Huilai V offshore wind farm project	1000	2623
45	Jieyang Shenquan I 350MW offshore wind farm	350	3220
46	Jieyang Jinghai 150MW offshore wind farm	150	2976
47	Jieyang Shenquan I offshore wind farm project	400	3229
48	Yangxi Shaba phase II offshore wind farm	400	2932
49	Huaneng Shantou lemen II offshore wind farm	402	2721
50	Datang Nanao lemen I offshore wind power project	399	2761
51	Nanao Yangdong offshore wind project	300	2596
52	CGN Huizhou Port 1 offshore wind farm	400	2895
53	CGN Huizhou Port 2 offshore wind farm	300	2921
54	Zhuhai Jinwan offshore wind farm project	300	3176
55	Shanwei Houhu 500MW offshore wind project	500	2695
56	Jiaxing No.1 offshore wind farm	300	2911
57	Jiaxing No.2 offshore wind farm	402	2657
58	Yuhuan No.1 offshore wind farm	400	2662
59	Zhejiang Shengsi No.2 offshore wind farm project	400	1807
60	Daishan No.4 offshore wind farm project phase I	216	2579
61	CGN Shengsi V offshore wind farm	132	2646
62	CGN Shengsi VI offshore wind farm	150	2641
63	Guodian Xiangshan I offshore wind farm	252	2638
64	Sanxia Yangjiang Qingzhou VI offshore wind farm	1000	2636
65	Sanxia Yangjiang Qingzhou V offshore wind farm	1000	2806
66	Guangdong Yangjiang Qingzhou II offshore wind	600	2614
67	Sanxia Yangjiang Qingzhou VII offshore wind farm	1000	3121
68	Guangdong Yangjiang Qingzhou I offshore wind	400	2483
69	CGN Yangjiang Fanshi II offshore wind farm	1000	3043
70	CGN Yangjiang Fanshi I offshore wind farm	1000	2720
71	Huadian Yangjiang Qingzhou III offshore wind farm	500	2689

72	Yangjiang Yangxi Shaba phase V offshore wind farm	300	2571
73	Mingyang Yangjiang Qingzhou IV offshore wind farm	500	2714
74	Yangjiang Yangxi Shaba phase IV offshore wind farm	300	2571
75	Yangjiang Yangxi Shaba phase III offshore wind farm	400	2695
76	Mingyang Yangjiang Shaba 300MW project	300	2761
77	Shantou Zhongpeng III offshore wind farm	1000	2790
78	Shantou Zhongpeng II offshore wind farm	1000	2769
79	Shantou Zhongpeng I offshore wind farm	1000	2739
80	Shantou Nanpeng III offshore wind farm	1000	2739
81	Shantou Nanpeng II offshore wind farm	1000	2770
82	Shantou Nanpeng I offshore wind farm	1000	2734
83	Shantou Qinpeng IV offshore wind farm	500	2734
84	Shantou Qinpeng III offshore wind farm	1000	2689
85	Shantou Qinpeng II offshore wind farm	1000	2717
86	Shantou Qinpeng I offshore wind farm	1000	2717
87	Huaneng Shantou Haimen wind farm	550	2717
88	Guangdong Shantou Haimen offshore wind farm	700	2745
89	Guangdong Zhanjiang Xinliao offshore wind project	203.5	2784
90	Guangdong Zhanjiang Wailuo offshore wind project II	203.5	2597
91	Zhanjiang Xuwen offshore wind project	600	2527
92	CGN Shanwei Jiazi-I offshore wind farm project	500	2495

2. Offshore wind Wake effect and Turbine layout model

To improve wind farm efficiency in the targeted area selected for offshore wind development, turbine layout is optimized to minimize turbine interference, with wake effect as the main considerations. Adopting a 2-dimension wake model, turbine layout is arranged with a generic algorithm. A multi-layer wind farm topology is also proposed for the practical demand of developing wide-area, large-scale offshore turbine groups.

2.1. Wake effect model

Wake effect refers to that wind speed decreases after passing through the front turbine, thus causing interference downstream. This interference is reflected mainly in the reduction of power generation in our model. Our wake effect model reveals axial wake trend with regular Jensen model, and regulates radial wake trend following the rule of Gaussian distribution (presented in Fig. S10). Coefficients of this distribution are obtained through the parameter fitting of abundant real-world wind field data. The effectiveness of this model has been demonstrated in field surveys[21].

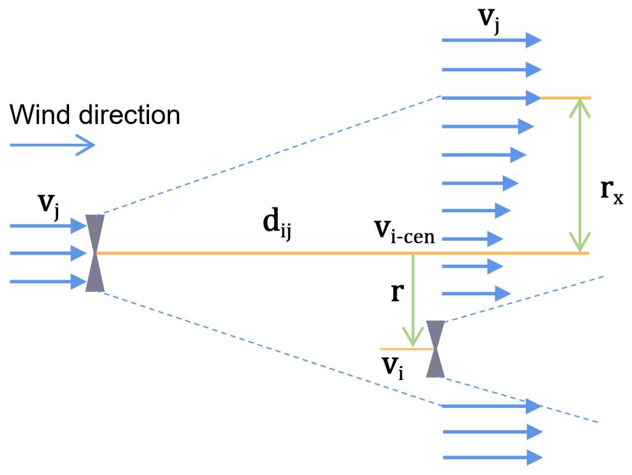

Fig S10: 2-dimention wake effect model. The axial wake trend (along the orange line) is regulated with conventional Jenson model, while the radial wake trend (along the green arrow) is regulated with Gaussian distribution. Maximum radial influence range is presented with r_x on the figure, where wind speed restores to the original wind v_j.

Adopting the 2-dimention wake effect model, local wind speed of the downstream turbine could be formulated as:

$$v_{i_{cen}} = v_j - v_j \times \lambda_{axial} \tag{17}$$

$$v_i = v_j - \left(v_j - v_{i_{cen}}\right) \times \lambda_{radial} \tag{18}$$

Among it:

$$\lambda_{radial} = \frac{5.16}{\sqrt{2\pi}} \times e^{\frac{-kr^2}{2\times\left(\frac{r_x}{2.58}\right)^2}} \tag{19}$$

$$\lambda_{axial} = \frac{1+\sqrt{1-C_t^j}}{\left(1+k\times\frac{d_{ij}}{R}\right)^2} \tag{20}$$

$$r_x = \alpha \times d_{ij} + R \tag{21}$$

$C_t$denotes the thrust coefficient, related to the local wind speed and turbine type. k denotes the surface roughness coefficient, ranging from 0.04 to 0.08 from offshore farm to the onshore one. V_{i_cen} denotes the wind speed at downstream central wake area (orange line in Fig S10). V_i denotes the wind speed at downstream turbine site, spacing a radial offset with the central wake area. r_x corresponds to the maximum radial influence range, which equals to the blade radius at upstream turbine, and scaled up with downstream distance (d_{ij}). Determination of each parameter refers to the study conducted by Beatriz Pérez et al[22]. To deal with the overlapped wake area generated by all the upstream wind turbines, D_{ij} is introduced to represent the interference from turbine j to turbine i. Thus, the overlapped wake effect, along with the local wind speed of turbine i could be formulated as formula (22)-(24).

$$D_{ij} = 1 - \frac{v_i}{v_j} = \lambda_{radial} \times \lambda_{axial} \tag{22}$$

$$D_i = \left(\sum_{\forall j} D_{ij}^2\right)^{0.5} \tag{23}$$

$$v_i = v_{in} \times (1 - D_i) \tag{24}$$

Where v_{in} denotes the wind farm incoming wind speed. Power generation is then derived from the NREL general 8MW offshore wind turbine model[19]. This model is based on the typical features seen in all the variable-speed pitch-control offshore wind

turbines, representing mainstream choice in latest committed offshore wind projects. Turbines start for the power generation at the “cut-in” wind speed, and gradually power up approaching the cubic relationship with wind speed until rated power level. With further increase of wind speed, turbines feather the blades to maintain constant power output, and shut down after reaching the “cut-out” wind speed. Notably, an empirical smooth correction is employed to the shoulder region near rated power point, thus representing the actual behavior of turbine power output and the clustering features of offshore wind farm.

Table S6: basic information of NREL 8MW offshore wind turbine model.

Coefficient	Value	Index
Rated power level	8MW	Nameplate power output
Cut-in wind speed	4m/s	Minimum operating wind speed
Rated wind speed	12m/s	Reach rated power level
Cut-out wind speed	25m/s	Maximum operating wind speed
Turbine Hub Height	112m	-
Turbine Rotor Diameter	180m	-
Turbine Specific Power	314W/m^2	Power generated per unit rotational area

To partly compensate for the turbulence generated on the downstream wind field, a correction is also introduced to revise the roughness coefficient[21].

$$\theta_0 = 0.1 \quad (25)$$

$$\theta_d = 0.8 \times \left(\frac{2R}{d_{ij}}\right)^{0.5} \quad (26)$$

$$\theta_{tur} = \left(0.4\theta_d + \sqrt{\theta_0}\right)^2 \quad (27)$$

$$\epsilon_{tur} = \frac{\theta_{tur}}{\theta_0} \quad (28)$$

$$k' = \epsilon_{tur} \times k \quad (29)$$

Suppose that the incoming wind condition is (v, θ). The wind farm coordinate is then rotated for θ, oriented according to the incoming wind. By doing this, the axial and radial distance in wake effect evaluation is simply equivalent to ΔY and ΔX among front and downstream wind turbines[22].

$$(x', y') = (\begin{bmatrix} \cos\theta & \sin\theta \\ \sin\theta & \cos\theta \end{bmatrix} \times (x, y)^T)^T \quad (30)$$

Thus, the local wind speed of turbine i can be formulated as:

$$\frac{v_i}{v_{in}} = 1 - (\sum\nolimits_{\forall j(Y_j' < Y_i')} (\frac{5.16}{\sqrt{2\pi}} \times exp(\frac{-k(X_i' - X_j')^2}{2 \times (0.385 \times \alpha \times (Y_i' - Y_j') + R)}) \times \frac{1 + \sqrt{1 - C_t^j}}{\left(1 + k' \times \frac{Y_i' - Y_j'}{R}\right)^2})^2)^{0.5} \quad (30)$$

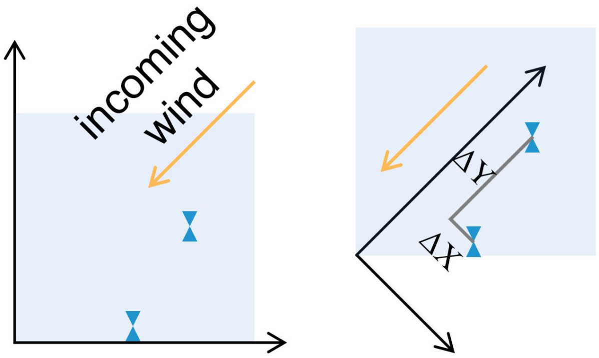

Fig S11: Rotating process of offshore wind farm coordinates. Axial and radial distance in wake effect evaluation is determined as ΔY and ΔX among front and downstream wind turbines.

Employing above 2-dimension wake model, wind conditions for the Anholt WF are presented in Fig S12 (under 12m/s upstream wind speed and 60° incoming wind direction). This wind farm is located at 16 km north of Denmark, combined with 111 Siemens SWT offshore turbines rated at 3.6MW[23].

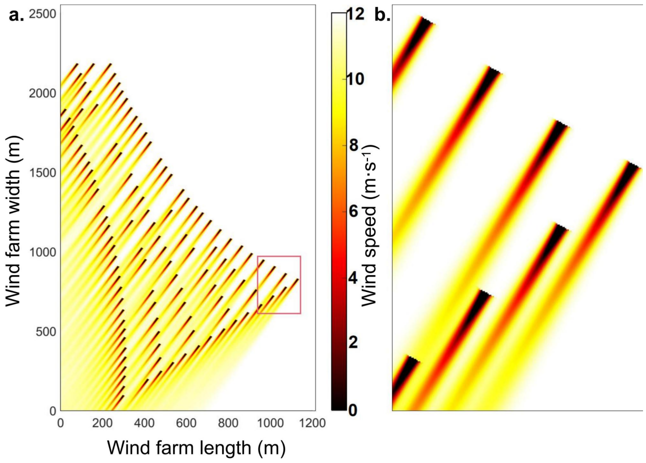

Fig S12: Wake effect of Anholt WF offshore wind farms. (a) Wake effect under 12m/s upstream wind speed and 60° incoming wind direction. Different wind speed is characterized with colored area, with associated color-bar calibrated on the right. (b) Local magnification of the wake area surrounded by a rectangle in Fig S12-b.

2.2. Turbine layout model

Wind farm turbine layout is optimized to minimize the turbine interference (mainly the wake effect) in a pre-selected sea area targeted for offshore wind development. To facilitate wake effect evaluation, data aggregation was employed to integrate hourly wind information covering the past 5 years and generate typical wind conditions for the wake effect calculations. Under these conditions, the wake effect is simulated and minimized by optimizing the turbine layout[24]. Generic algorithm is employed here for the layout arrangement. In this algorithm, dozens of layout schemes are randomly initialized, tested and updated through thousands of iterations. In each iteration, layout schemes with superior wake efficiency are typically more likely to be selected and generate even superior layout schemes in fixed procedures (including replication, crossing and variation). A penalty module is embedded to guarantee the minimum interval required between adjacent turbines. This module imposes extra punishment to the unqualified turbine pairs in each iteration. The flow diagram for this process is presented in Fig. S13.

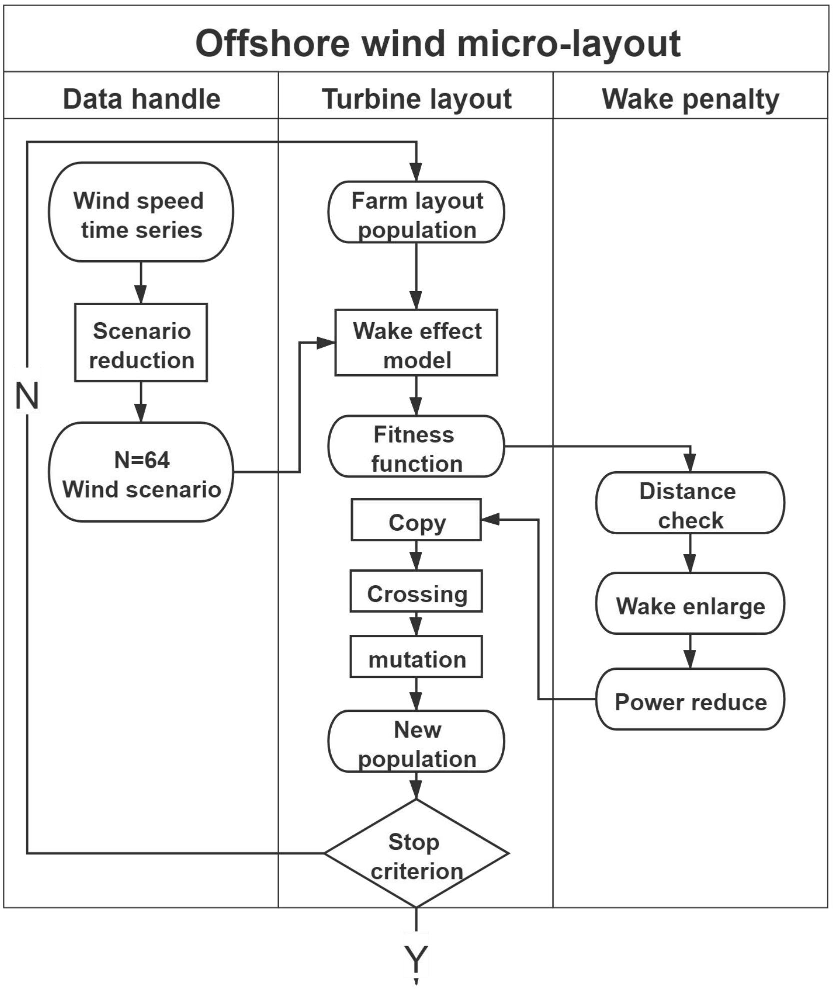

Fig S13: Overall diagram of offshore wind turbine layout algorithm.

2.2.1. Turbine coding

Targeted area for offshore wind development is divided into fixed grid cells. Turbines could only be located at the grid cross points. The location of each turbine is converted into a binary code. Each layout scheme is coded into a binary string, and all the layout schemes are coded into a binary matrix. Related coding processes, with associated components in generic algorithm are presented in Fig S14.

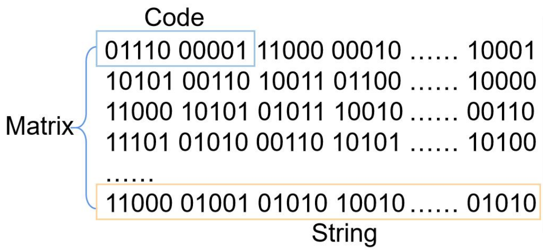

For wind farm	For Coding	For GA
Location of each turbine	Binary code	gene
Location of all turbines for an alternative farm layout	Binary string	individual
All the farm layouts	Binary matrix	population

Fig S14: Coding process of offshore turbine coordinates in generic algorithm. Binary matrix for all the turbine coordinates in each layout scheme is presented on the left, while the corresponding relationship in wind farm, binary coding and generic algorithm are presented on the right.

2.2.2. Data aggregation

To facilitate wake effect evaluation, data aggregation was employed to integrate the hourly wind field data in last 5 years and generate typical wind conditions for the wake effect calculations. Hourly wind field data could be regarded as equal-weighting scenarios. Scenario aggregation loops through all the scenarios, integrates the most similar pair and aggregates their weightings. Above procedure is repeated to integrate original data (amounts to 43800) until the targeted quantity (64 in our model). Data aggregation method accelerates the calculation more than 200 times with negligible deviation in results relative to the rigorous method. Three procedures for the data aggregation are listed below:

2.2.2.1. Scenario mapping

Given the wind power density is proportional to the cubic of wind speed, wind field data (v, θ) is mapped to the targeted space (v^3, θ). In this space, distance between adjacent points could be employed as the criterion to judge their similarity. Weight of each point, along with the distance between all the pairs, are presented in the matrix W and D, respectively.

$$\boldsymbol{W} = \begin{bmatrix} \omega_1 \\ \omega_2 \\ \omega_3 \\ \cdots \\ \omega_n \end{bmatrix} \tag{32}$$

$$\boldsymbol{D} = \begin{bmatrix} 0 & d_{12} & \cdots & d_{1n} \\ d_{21} & 0 & \cdots & d_{2n} \\ \cdots & \cdots & \cdots & \cdots \\ d_{n1} & d_{n2} & \cdots & 0 \end{bmatrix} \tag{33}$$

The weighted distance G is formulated as below, the symbol *ones (1, n)* defines a row vector filled with 1xn ones.

$$G = \boldsymbol{W} \times ones(1, n) \cdot \boldsymbol{D} \tag{34}$$

2.2.2.2. Scenario reduction

The minimum element among matrix G is selected (suppose G_{ij}), then the point i is aggregated to point j in the form of probability.

$$\begin{cases} \omega_i = \Phi \;\; \omega_j = \omega_i + \omega_j \\ D(i,:) = \Phi \;\; D(:,i) = \Phi \end{cases} \tag{35}$$

2.2.2.3. Scenario check

The above process loops until wind scenario are aggregated to designated quantity.

2.2.3. Turbine layout arrangement

Local wind speeds for each turbine are evaluated with wake model aforementioned in last subsection. For each turbine layout scheme (individual in the generic algorithm), average wake efficiency η among all the wind scenarios is calculated and employed as the fitness function.

$$\eta = \sum_{s=1}^{N_{scenario}} \frac{\sum_{i=1}^{N_{turbine}} f(v_i(s))}{N_{turbine \times f(v(s))}} \times \rho_{scenario} \tag{36}$$

f denotes the power generation curve for the NREL 8MW offshore turbine model. $N_{turbine}$ x f (v(s)) denotes the ideal power generation without considering wake effect. $\sum_{i=1}^{N_{turbine}} f(v_i(s))$ denotes the practical power generation derived from the local wind speed of each turbine. In the generic algorithm, wake efficiency η for each layout scheme is calculated and employed as the fitness function. Farm layouts with superior wake efficiency are more likely to remain and generate even upgraded layout schemes through fixed procedure. The overall procedure is presented in Fig. S15.

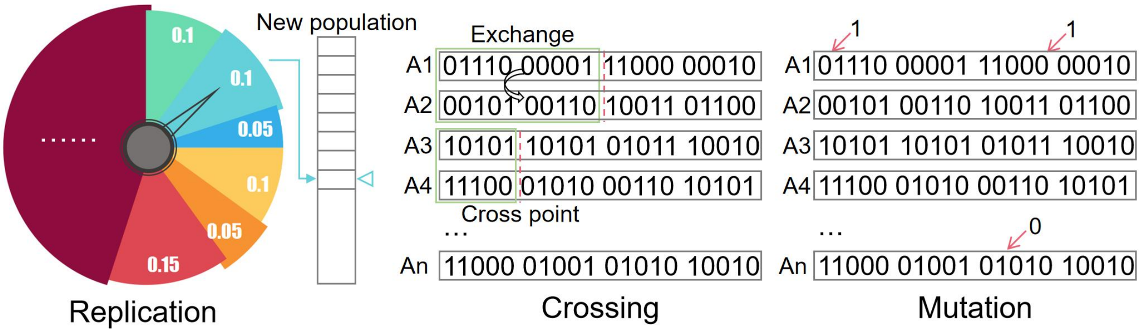

Fig S15: three major procedures in generic algorithm.

Aforementioned fixed procedure could be summarized as: (a) Replication: each population in generic algorithm is presented on the roulette as a sector, with an angle related to its fitness function. Individuals are randomly selected and fully copied with the probability proportional to their sector angles, until forming a new population with the same individual quantity as the original one. (b) Crossing: for adjacent individuals to be mated, a crossover point is randomly chosen and an offspring is generated by exchanging genes on corresponding position until reaching the crossover point. (c) Mutation: all the genes in each individual have a certain probability to be flipped, to maintain diversity within population and prevent premature convergence. After the above three procedures, an obtained binary matrix will participate in next iteration. For each iteration, the fitness function of the best individual is recorded, and a deviation below 0.1% among the latest 100 iterations is regarded as the stop criterion.

A wake penalty is also employed to ensure the distance constraints, in which 8 times of turbine radius is required as the minimum interval to separate adjacent turbines. In the wake penalty process, distance among adjacent turbines is additionally checked in each iteration, power generation drops and wake region expands for all the unqualified turbines.

2.2.4. Convergence cable wiring

Based on the turbine layout obtained in previous subsection, wind farm cable wiring is optimized to minimize total convergence cable length. Radial convergence topology is employed in our model, with turbine power generation gathered at the collector through multiple cable strings. Related routing arrangement is recorded in

the matrix F. Each row denotes a single string, index from front to back denotes connection order from tail turbine to the collector. An annealing algorithm is then conducted to optimize the cable wiring scheme.

Controlling coefficient T is initialized at 10^6 and gradually descends as the iteration progresses. In each iteration, two different indexes in assignment matrix are randomly selected for exchange. This exchange is finally conducted with a possibility related to the total cable length before and after this procedure.

$$P=\begin{cases}1 & L^{'}<L\\ \exp\left(-\frac{L^{'}-L}{KT}\right) & L^{'}>L\end{cases} \tag{37}$$

L′ and L denote the total convergence cable length before and after the exchange. K denotes the Boltzmann constant. T remains unchanged during dozens of exchanges as a guarantee of process stability (called Markov Chain), and then multiplied with a cooling coefficient to activate the next iteration. The iteration stops when T reaches 10^{-3}, and the index recorded in matrix F is employed as the final routing scheme.

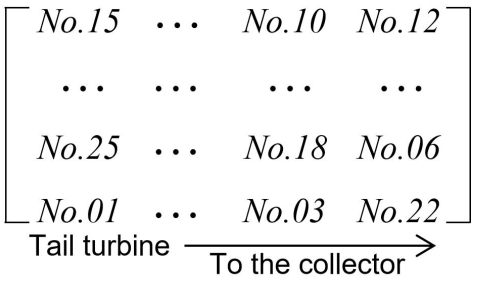

Coefficient	value
Initial Controlling coefficient	10^6
Final Controlling coefficient	10^{-3}
Markov chain	50
Cooling coefficient	0.995

Fig S16: Distribution matrix F and related coefficient in annealing algorithm. The turbine routing scheme in the matrix F is presented on the left. Each row denotes a single convergence cable string from tail turbine to the collector. Assignment of major coefficients in the annealing algorithm is presented on the right.

Given the practical demand of developing large-scale offshore turbine groups in the southeast coast China, a wide-area wind farm topology is established, to trade off the (a) wake effect efficiency (b) spatial utilization rate (c) potential for future expansion. Shipping lanes, maintenance channel, convergence route, as well as the space reserved for all the system facilities are incorporated in our consideration. The wide-area wind farm topology is presented in Fig S17. Targeted placements for offshore wind development are divided into irregular clusters, to facilitate vessel

traffic, farm maintenance and future potential expansion. For a single cluster, power generation is converged, delivered and grid committed as a whole. Each cluster consists of several wind farms, spacing a fixed interval to weaken the interference among adjacent ones. Passageway is also reserved in each wind farm to facilitate turbine installation, operation and maintenance, while further weakens the turbine interference.

Under this topology offshore turbines are placed 5MW/km^2 to guarantee a 95% wake efficiency, and 4MW/km^2 in dense shipping areas[25] where the SO_2 emission rate is higher than 10^{-11}kg·m^{-2}·s^{-1}. This estimation is similar to the 7D×7D wind turbine interference proposed by Musial et al[26], corresponding to a deployment density for 8MW wind turbines of one per 1.6km^2.

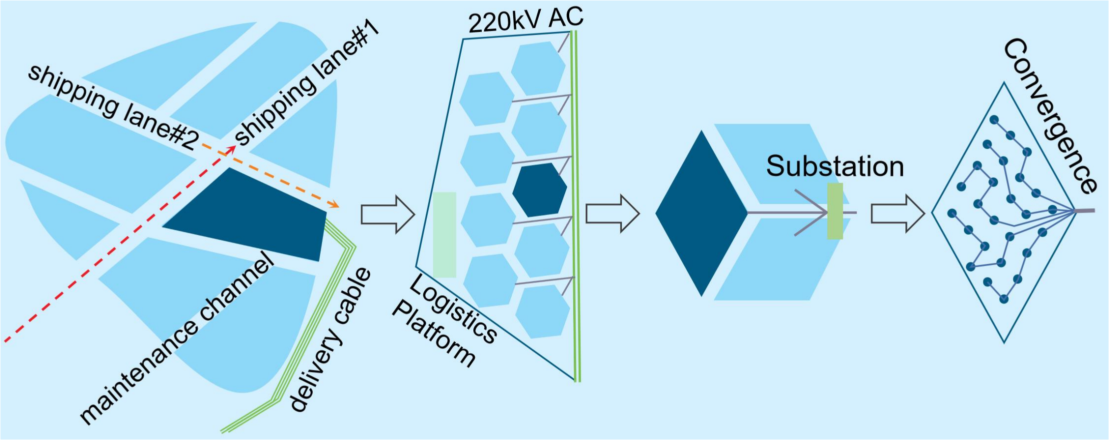

Fig S17: Multi-layer wind farm topology proposed for the large-scale offshore wind turbine in the southeast coastal China.

2.3. Data derivation

Offshore wind field data are derived from NASA's MERRA2 dataset, a reanalysis product that defines the hourly wind speed with a spatial resolution of 0.5 degree latitude by 0.625 degree longitude from 1980 to the present[27]. Real-world wind speed at 10m and 50m altitude are extrapolated to 100m using power law. Offshore bathymetry data are derived from GEBCO One Minute Grid, a dataset providing water depth data at 1-arc min resolution[28]. MERRA-2 grid is then rescaled to the high-resolution GEBCO grid to combine and fully take advantage of above two. The

cell considered for physical resource assessment is sized 5×5km, capable for 125MW offshore wind development. Provincial jurisdiction on the ocean regions is delineated by the nearest distance. Each targeted area belongs to the province whose coastline has the nearest airline distance. Local water depth, annual average wind speed, along with the SO_2 emission status are presented in the Fig S18.

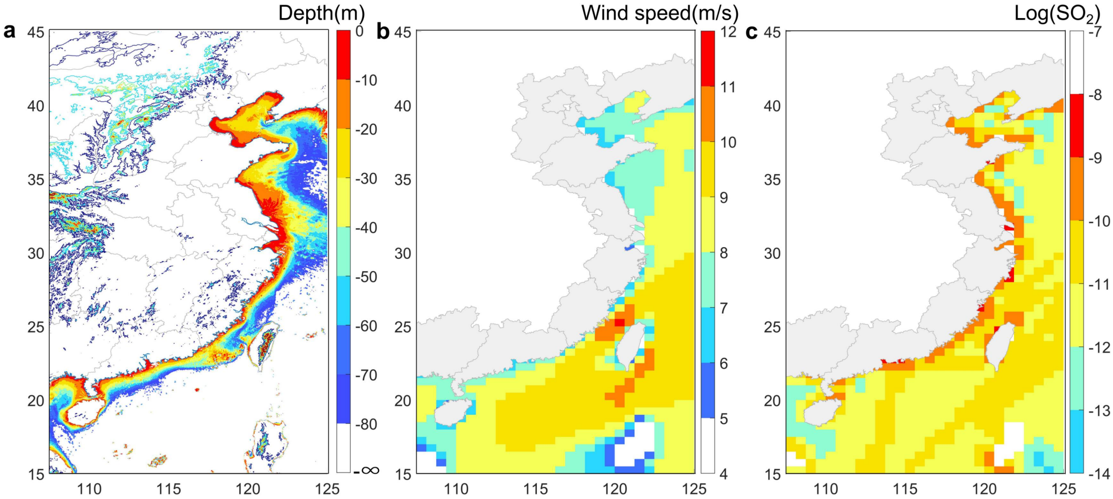

Fig S18: Basic information of southeast coastal China. (a) Local water depth from 0-80m, locations with water depth higher than 80m are colored white. (b) Annual average wind speed in the resolution of 50 × 50km. (c) Annual average emission rate of sulfur dioxide, presented in the form of logarithm. Location with emission rate higher than 10^{-10}($kg \cdot m^2 \cdot s^{-1}$) is defined as the dense shipping area, with 20% of the potential capacity removed as a reserve for shipping lanes.

Notably, shallow water regions extend relatively farther at southeast coast China. If offshore wind power were to become a substantial part in the generation mix, fixed base technology is sufficient to provide needed capacity and will likely be the dominant option in the future offshore wind projects. The physical potential along the east coast is regarded as greater than south given the shallow water region extends farther. Quality of wind resources is greatest near the junction of the eastern and southern sea areas.

Economics of offshore wind depend on the balance between its levelized cost and local benchmark price for coal-fire or nuclear units (proposed as 0.07 and 0.085 USD per kWh). The overall conditions for potential area targeted for offshore wind development in the southeast coast are presented in Fig S19. Each point denotes sea

area capable for 8GW offshore wind installation, with local capacity factor expressed by circle size and provincial regions characterized by circle color.

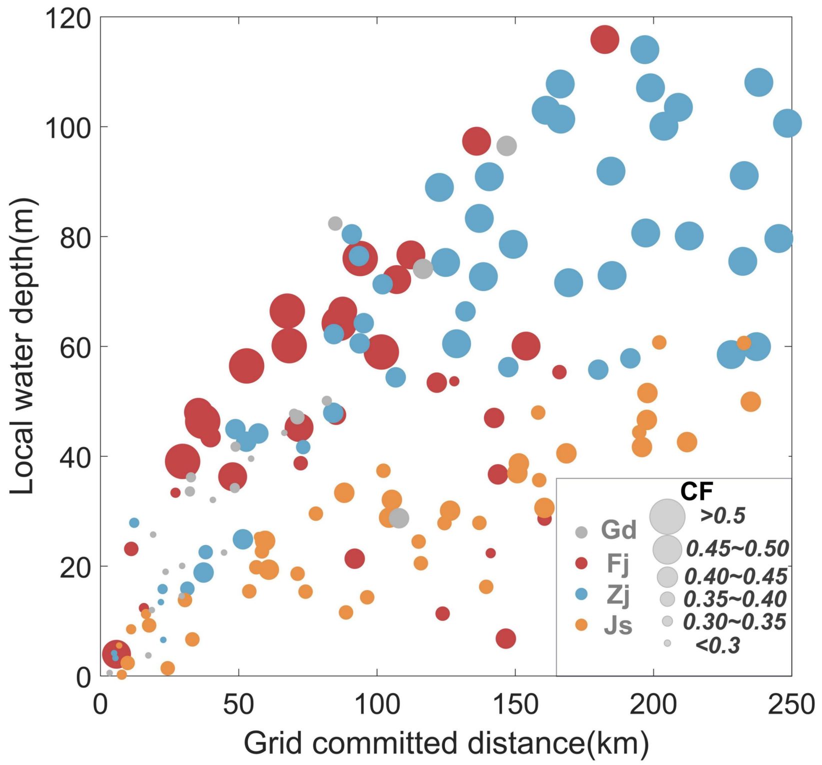

Fig S19: Overview of offshore wind resources in major provinces for offshore wind installation. Each circle denotes a sea area capable for 8GW offshore wind development. Capacity factor is expressed by the circle size and provincial jurisdiction is characterized by circle color, with relate legend tagged on the bottom right.

3. Offshore wind Power system simulation model

Power system simulation is conducted on an hourly basis throughout an entire year, simulating and optimizing the system operations while estimating the potential curtailment rates. The curtailment rates are evaluated at every wind power investment level for onshore wind in the "Three North" regions and offshore wind in the coastal regions. A block diagram for power system simulation model is presented in Fig S20.

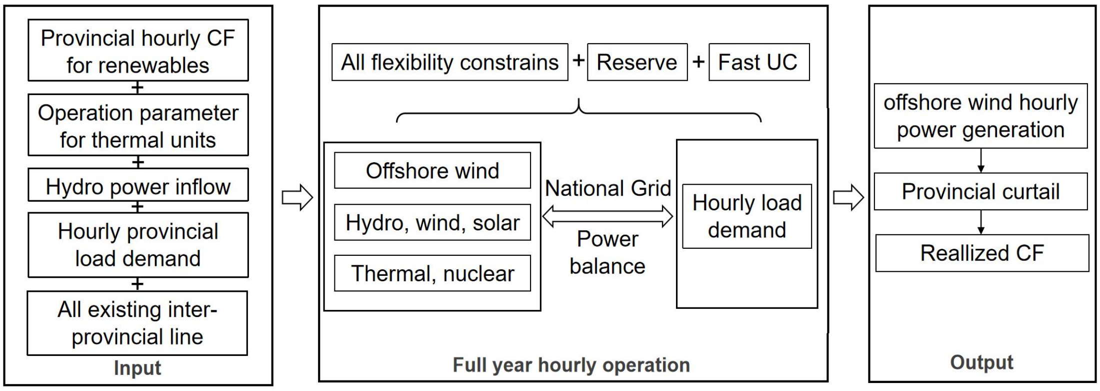

Fig S20: Block diagram of the power system simulation model.

Unit commitment is employed to simulate and optimize the system operations in 2020. All the flexibility constraints for the thermal units are considered, including unit ramping limits, minimum online-offline times, minimal output levels and must-run constraints. Inter-provincial AC transmissions are freely dispatched at an hourly basis, allowing for the bi-directional power flows between connected provinces. While DC transmissions are dispatched adopting fixed strategy and allows only for the uni-directional power flow. The hourly utilization rates for all the DC transmissions remain 90% during the day, while fall to 50% throughout the night. Hourly power balance and reserve constraints are attached to each province. The curtailment rates and realized capacity factors are then evaluated by the derived onshore and offshore wind power output time-series.

High-precision generation and consumption datasets are employed in our power system simulation model. For the generation sector, detailed operation parameters for over 3000 thermal units within the national jurisdiction are incorporated. Fuel cost for

each thermal unit is also evaluated based on both the fuel consumption characteristics and the provincial supplied cost of fossil-fuel. Hourly capacity factor for onshore, offshore wind and solar PV are incorporated on the provincial basis. Natural inflow, typical hydro height and reservoir capacity are also employed for all major hydro power stations. For the consumption sector, hourly provincial load demand, heating period are also incorporated in our model.

3.1. Decision variables

Decision variables for the system simulation model only include the committed status and dispatched capacity for each generation category, hourly schedule for the inter-provincial transmissions. Fast unit commitment (FUC) is employed in our model to improve the computational efficiency for system at such a large scale[29]. Instead of scheduling the committed and dispatched behaviors for thousands of individuals at each time interval, thermal units in each provincial region are aggregated according to the fuel type, nameplate capacity, whether CHP units or not. The FUC significantly reduces the computational complexity, allowing for the simulation at the hourly basis over an entire year, facilitating the incorporation of full set of flexibility constraints. The modeling error decreases with the system size. At such national level simulation, our fast unit commitment model's modeling error is negligible and estimated less than 3%.

To verify the effectiveness of FUC method, we simulate weekly scheduling for the Northwest regional power grid based on both the FUC model, and the conventional UC model. Hourly operation results of the regional power grid are shown in Figure S21. According to the simulation results of above two unit commitment models, the hourly dispatched capacity for all the units in different categories are nearly the same. The total operational cost for above two modeling methods deviates by less than 3%. Compared with the conventional model, the proposed FUC model could accelerate the computational process by 20000 times, making the operation simulation of large-scale power system computationally possible.

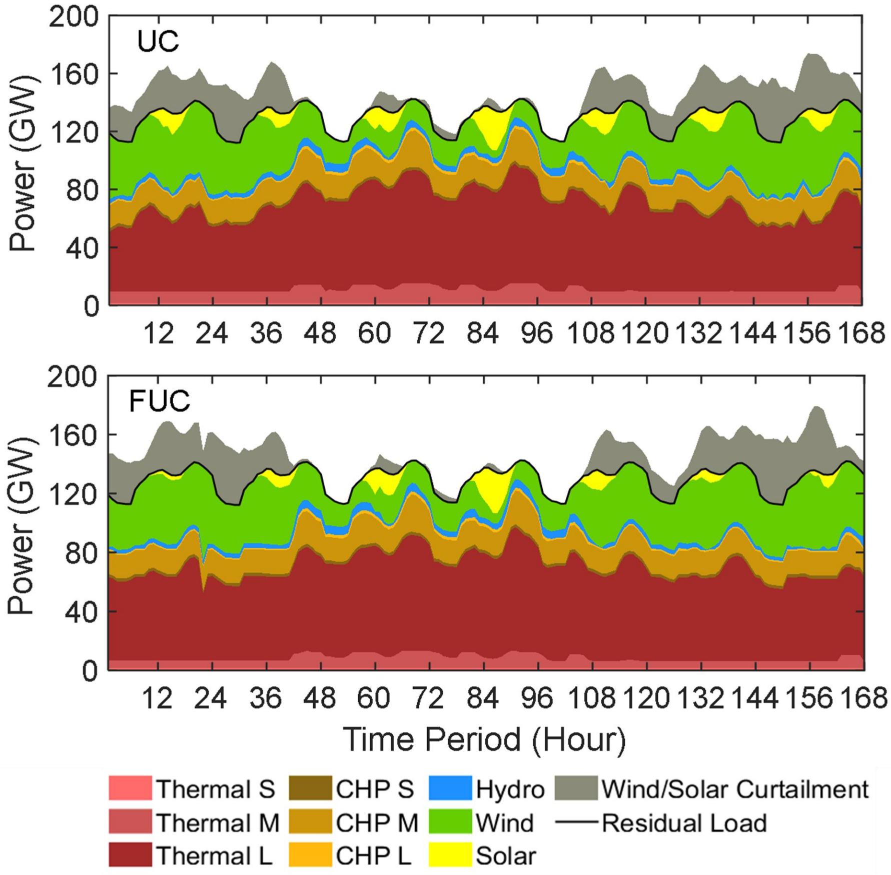

Fig. S21 Comparison of operation simulation results solved by the conventional UC (top) and proposed FUC (bottom) models. We take the hourly power balance in Northwest China for the sample week as an example. The technical parameters for almost all thermal units (over 500) in NW China are surveyed to build the UC model, and then the three different sizes of groups (small, medium, and large) for coal, gas, and CHP units can be created by the FUC techniques.

Based on the proposed FUC methods, four continuous variables are employed for the thermal status description. For the thermal group j at province k.$X_1^{j,k}(t)$ and $X_0^{j,k}(t)$ denote the total committed and real-time dispatched capacity at time t, respectively. $X_2^{j,k}(t)$ and $X_3^{j,k}(t)$ denote the capacity scheduled to be on-line or off-line at time t, simulating the startup and shutdown operations for the aggregated thermal clusters. $X_1^{j,k}(t)$ and $X_0^{j,k}(t)$ are constrained within the total installation for this thermal group $X^{j,k}$ and could be formulated as:

$$\begin{cases} 0 \le X_1^{j,k}(t) \le X_0^{j,k}(t) \le X^{j,k} \\ X_1^{j,k}(t) - X_1^{j,k}(t-1) = X_2^{j,k}(t) - X_3^{j,k}(t) \end{cases} \tag{38}$$

Power generation for all the renewables is restricted jointly by local installation

Cap^{k}_{renew} and real-time capacity factor $CF^{k}_{renew}(t)$, formulated as (39). Multiplication of above two is defined as the renewables output potential, functions as the upper bound for the renewables power generation. The deviation between output potential and practical power generation is defined as the renewable curtailment.

$$0 \leq P^{k}_{renew}(t) \leq CF^{k}_{renew}(t) \times Cap^{k}_{renew} \tag{39}$$

For the inter-provincial transmissions, capacity available for scheduling is bounded within the transmitted capacity.

$$\begin{cases} -P^{k,l}_{AC-ini} \leq P^{k,l}_{AC}(t) \leq P^{k,l}_{AC-ini} \\ 0 \leq P^{k,l}_{DC} \leq P^{k,l}_{DC-ini} \end{cases} \tag{40}$$

$P^{k,l}_{AC}$ and $P^{k,l}_{DC}$ denote the capacity rating for AC and DC transmissions between province k and province l. DC line is uni-directional transmitted while AC line is capable for flow reversion. Negative bound for AC transmission limits the counter flows from province l to province k.

3.2. Objective function

Power system simulation is optimized by minimizing the overall operational cost, including start-up cost and fuel cost for thermal units, operation cost for nuclear plants, and maintenance cost for each generation category. Objective function of the system simulation model could be formulated as:

$$Oper = \sum_{t=1}^{T} \sum_{k=1}^{N_{reg}} \sum_{j=1}^{N^{k}_{th}} \left(C^{j,k}_{th}(t) + U^{j,k}_{th}(t) + D^{j,k}_{th}(t) \right) + \sum_{k=1}^{N_{reg}} OM^{k} \tag{41}$$

N^{k}_{ther} denotes the number of thermal clusters in province k, taken as 12 for each province. N^{k}_{reg} denotes the number of provinces considered in our power system simulation. This model is based on the overall 34 provincial regions in China. Having considered regional conditions and grid connected situations, we integrate Hong Kong and Macao to Guangdong, regard JinJingJi as a whole, remove Tibet and Taiwan, also divide Inner Mongolia to the eastern part and western part. T denotes the number of time intervals considered in the unit commitment simulation, taken as 8760 for the hourly resolution throughout a year. For the jth thermal cluster in the province k,

$C_{th}^{j,k}(t)$, $U_{th}^{j,k}$ and $D_{th}^{j,k}$ denote the fuel cost for normal generation, start up and shut down operations. A linear coal consumption relationship is adopted here, employing continuous variables to specify the fuel cost for each thermal cluster[30].

$$\begin{cases} C_{th}^{j,k}(t) = A_{th}^{j,k} \times X_0^{j,k}(t) \\ U_{th}^{j,k} = K_{th-up}^{j,k} \times X_2^{j,k}(t) \\ D_{th}^{j,k} = K_{th-down}^{j,k} \times X_3^{j,k}(t) \end{cases} \tag{42}$$

For the jth group at province k, $A_{th}^{j,k}$ denotes the fuel cost at per kWh basis. The fuel consumption relationship of coal, gas, CHP coal, and CHP gas refers to the researches proposed by Xinyu Chen et al[31] and Haiwang Zhong et al[32]. $K_{th-up}^{j,k}$ and $K_{th-down}^{j,k}$ define the fuel costs for the startup and shut down capacity at per kWh basis[35]. Above parameters are determined as capacity-weighted average value of all the clustered thermal units.

OM denotes the operation and maintenance costs for all the generation categories. This cost is taken as 15% of the amortized investment cost for onshore wind power[36], 20% for offshore wind power, 5% for solar PV[37], 20% to 30% for thermal units.

3.3. Flexibility constraints

Operational characteristics for thermal group are described by flexibility constraints, including ramping limits, minimum on-off time requirements, minimum load levels, must run units[28]. We note that these constraints are of vital importance with elevated renewable penetration, given system flexibility provided by thermal units is largely demanded for renewables integration. Ignorance of any constraints mentioned above may lead to significant under-estimation of renewable curtailments.

3.3.1. Ramping constraints

Ramping constraints based on the continuous decisions are formulated as:

$$\begin{aligned} X_0^{j,k}(t) - X_0^{j,k}(t-1) &\leq \underline{A}_j \times X_2^{j,k}(t) - \underline{A}_j \times X_3^{j,k}(t) + R_U^{j,k}(X_1^{j,k}(t) - X_2^{j,k}(t) - X_3^{j,k}(t+1)) \\ X_0^{j,k}(t) - X_0^{j,k}(t-1) &\geq \underline{A}_j \times X_2^{j,k}(t) - \underline{A}_j \times X_3^{j,k}(t) - R_D^{j,k}(X_1^{j,k}(t) - X_2^{j,k}(t) - X_3^{j,k}(t-1)) \end{aligned} \tag{43}$$

$R_U^{j,k}$ and $R_D^{j,k}$ denote the ratios of hourly upward and downward ramping potential for jth thermal group at province k. $\underline{A}_j$ denotes the power generation lower bound for jth thermal group at province k, required as 0.5 for coal-fired units and 0.25 for gas-fired units[30]. Capacity committed online is restricted by the potential startup ramping from 0 at time t-1 to $\underline{A}_j \times X_2^{j,k}(t)$ at time t. Capacity committed offline is restricted by the potential shutdown ramping from $\underline{A}_j \times X_2^{j,k}(t)$ at time t-1 to 0 at time t. $X_1^{j,k}(t) - X_2^{j,k}(t) - X_3^{j,k}(t+1)$ denotes capacity continuously operates from time (t-1) to time (t+1), providing normal upward ramping potential. $X_1^{j,k}(t) - X_2^{j,k}(t) - X_3^{j,k}(t+1)$ denotes capacity continuously operating from time (t-2) to time t, providing normal downward ramping potential[29]. A further constraint for the maximum power generation boundary for unit group j at time t is formulated as:

$$X_0^{j,k}(t) \leq \overline{A}_j \times (X_1^{j,k}(t) - X_2^{j,k}(t) - X_3^{j,k}(t+1)) + \underline{A}_j(t) \times X_2^{j,k}(t) + \underline{A}_j \times X_3^{j,k}(t+1) \quad (44)$$

$\overline{A}_j$ denotes the upper bound of power output ratio for jth thermal group, taken 1 for all the thermal units in our simulation.

3.3.2. Minimum on/off time constraints

Online thermal units cannot be shut down until they have been operating for designated time. T_j^U denotes the minimum online time required by thermal group j, the minimum online time constraints can be formulated as:

$$0 \leq X_3^{j,k}(t+1) \leq X_1^{j,k}(t) - \sum_{\tau=0}^{t-1} X_2^{j,k}(t-\tau) \quad 1 \leq t \leq T_j^U - 1$$

$$0 \leq X_3^{j,k}(1) \leq X_1^{j,k}(0) \quad (45)$$

$$0 \leq X_3^{j,k}(t+1) \leq X_1^{j,k}(t) - \sum_{\tau=0}^{T_j^U-2} X_2^{j,k}(t-\tau) \quad T_j^U \leq t \leq T-1$$

For the thermal group j at province k, $X_1^{j,k}(0)$ denotes the online capacity at initial time interval. When $t \in [1, T_j^U - 1]$, all the capacity previously committed online cannot

be shutdown. When $t \in [T_j^U, T\text{-}1]$, all the capacity committed online after time $(T\text{-}T_j^U+1)$ cannot be shutdown.

Similarly, offline units cannot be opened up until it has been shut down for designated time. T_j^D denotes the minimum offline time required by thermal group j, the minimum offline time constraints can be formulated as:

$$0 \le X_2^{j,k}(t+1) \le X^{j,k} - X_1^{j,k}(t) - \sum_{\tau=0}^{t-1} X_3^{j,k}(t-\tau) \quad 1 \le t \le T_j^D - 1$$

$$0 \le X_2^{j,k}(1) \le X^{j,k} - X_1^{j,k}(0) \tag{46}$$

$$0 \le X_3^{j,k}(t+1) \le X^{j,k} - X_1^{j,k}(t) - \sum_{\tau=0}^{T_j^D-2} X_3^{j,k}(t-\tau) \quad T_j^D \le t \le T-1$$

For the thermal group j at province k, $X^{j,k} - X_1^{j,k}(0)$ denotes the offline capacity at initial time interval. When $t \in [1, T_j^D\text{-}1]$, all the capacity previously committed offline cannot be opened. When $t \in [T_j^D, T\text{-}1]$, all the capacity committed offline after time $(T\text{-}T_j^U+1)$ cannot be opened. Coefficients T_j^U and T_j^D are also determined as the capacity weighted average of all the thermal units to be clustered.

3.3.3. Max-min load constraints

$\overline{A}_j(t)$ and $\underline{A}_j(t)$ denotes the upper and lower power generation levels. Dispatched capacity $X_0^j(t)$ is bounded within the specified range, formulated as:

$$\underline{A}_j(t) \times X_1^j(t) \le X_0^j(t) \le \overline{A}_j(t) \times X_1^j(t) \tag{47}$$

3.3.4. Must-run unit constraints

Must-run constraints are additionally attached to CHP units throughout the heating season in Northern regions[38]. Flexibility constraints as ramping limits are also required at a stringent standard during this period. The above constraints can be formulated as:

$$\begin{cases} X_1^{jc,k}(t) = X^{jc,k}(t \in t_h) \\ \left|X_0^{jc,k}(t) - X_0^{jc,k}(t-1)\right| \le R^{jc,k}(t \in t_h) \end{cases} \tag{48}$$

where j_c denotes the index for CHP units, and t_h denotes the heating seasons in Northern regions. Upward and downward ramping capacity during this period are determined both the $R^{jc,k}$.

3.4. Hydro power model

Hydro power in the simulation is modeled considering natural inflow, hydro height, reservoir capacity. Natural inflow for all major hydro power stations within national jurisdiction is determined based on the dataset CFSR. Reserve water in the reservoir could participate in both power generation and spillage process. The constraints for hydro power stations are presented as below:

3.4.1. Inflow constraints

$$\begin{cases} V^j(t) = V^j(t-1) + \frac{P_{hydro}^{j-1}(t-\tau)}{\eta^{j-1}\times H^{j-1}} + S^{j-1}(t-\tau) - \frac{G^j(t)}{\eta^j \times H^j} - S^j(t) \\ V^1(t) = V^1(t-1) + I(t) - \frac{P_{hydro}^1(t)}{H^1} - S^1(t) \end{cases} \quad (49)$$

Where $V^j(t)$ denotes the capacity of jth reservoir cascade in the selected waterway. $P_{hydro}^1(t)$ denotes the hydro power generation, which could be converted to the discharged water volume through head height H. Head height is simply regarded as constant in our model and evaluated by dividing the decadal water inflow with power generation during the same period. This processing method should not lead to any significant deviation from reality, given the fact that (1) water level usually remains bounded within a specified range, as a guarantee for both generation efficiency and dam security; (2) reservoir water level is negligible compared with the geographical elevation in a few hydro stations. η denotes the overall efficiency for the conversion from hydro power to electricity. $S^j(t)$ denotes the hydro spillage for jth hydro station, corresponding to the water directly discharged downstream without participation in power generation. $I(t)$ denotes the natural inflow at the first reservoir in the cascade waterway. τ_{j-1} refers to the required time interval for inflow to travel from reservoir (j-1) to reservoir j. Inflow participates in both power generation and water spillage at station (j-1) will gather directly at station j after this period.

3.4.2. Water level and discharge rate constraints

Maximum discharge rate and reservoir water level are restricted by hydro turbine capacity and reservoir volume. Initial and final water level is set to be 80% of the reservoir capacity in our model.

$$\begin{cases} 0 \le R^j \le R^j_{max} \\ 0 \le G^j \le G^j_{max} \\ R^j(t_{ini\&final}) = 0.8 \times R^j_{max} \end{cases} \tag{50}$$

3.4.3. Hydro reserve constraints

Hydro power station can also participate in system rotating backup. This backup capacity is constrained by not only the spared capacity for electric machinery, but the reservoir water residual at last time interval. Having considered the possible spillage control, hydro power reserve constraints can be formulated as below. $Res^j_{hy}(t)$ denotes the backup capacity available at jth hydro station.

$$\begin{cases} Res^j_{hy}(t) \le P^j_{hy-max} - P^j_{hy}(t) \\ V^j(t) - \frac{Res^j_{hy}(t)+P^j_{hy-max}}{\eta^j \times H^j} - S^j(t) \ge 0 \end{cases} \tag{51}$$

3.5. Power balance and reserve constraints

3.5.1. Power balance constraints

For each time interval and all the provincial regions, total power generation from each generation category should equal the load demand and power export.

$$\sum_{j=1}^{N^k_{ther}} X_0^{j,k}(t) + P^k_w(t) + P^k_{off}(t) + P^k_s(t) +$$

$$P^k_{nu}(t) + P^k_{hy}(t) + P^k_{EX-AC}(t) + P^k_{EX-DC}(t) = D^k(t) \tag{52}$$

Among it:

$$\begin{cases} P^k_{EX-AC}(t) = \sum_{l=1(l\neq k)}^{N_{reg}} P^{k,l}_{AC}(t) \\ P^k_{EX-DC}(t) = \sum_{l=1(l\neq k)}^{N_{reg}} P^{l,k}_{DC}(t) - \sum_{l=1(l\neq k)}^{N_{reg}} P^{k,l}_{DC}(t) \end{cases} \tag{53}$$

$D^k(t)$ refers to the power demand of province k at time t. $P_{EX-AC}^{k}(t)$ and $P_{EX-DC}^{k}(t)$ denote the AC and DC inter-provincial power exchange between region k and other provinces. Power inflow is defined as positive, otherwise is negative. Notably, $P_{DC}^{l,k}(t)$ and $P_{DC}^{k,l}(t)$ corresponds to different inter-provincial transmissions given the DC line considered in our model is uni-directional transmitted and unable to be reversed. $P_w^k(t)$ $P_{off}^k(t)$ and $P_s^k(t)$ denote the power output for onshore wind, offshore wind and solar PV in region k at time t, respectively.

3.5.2. Reserve constraints

$$\sum_{j=1}^{N_{the}^{k}} \left(\overline{A}^{j,k} \times X_1^{j,k}(t) - X_0^{j,k}(t)\right) + C_w^{cre} \times \left(CF_w^k(t) \times Cap_w^k(t) - P_w^k(t)\right) + C_{off}^{cre} \times \left(CF_{off}^k(t) \times Cap_{off}^k(t) - P_{off}^k(t)\right) + C_s^{cre} \times \left(CF_s^k(t) \times Cap_s^k(t) - P_s^k(t)\right) + \sum_{j=1}^{N_{hy}^{k}} Res_{hy}^{j,k} \leq C_{load}^{Res} \times D^k(t) + C_{renew}^{Res} \times \left(P_w(t) + P_s(t) + P_{off}(t)\right) \quad (54)$$

Where $\overline{A}^{j,k}$ denotes the maximum power generation ratio of jth thermal group. $\overline{A}^{j,k} \times X_1^{j,k}(t) - X_0^{j,k}(t)$ is defined as the rotating backup of thermal group j, referring to the capacity committed online but in no-load state. Reserve capacity for renewables is evaluated by subtracting the potential energy with real-time power output and multiplied by a confidence factor as an offset for its un-reliability in output pattern. Res_{hy} denotes the backup capacity provided by hydro stations as mentioned above. $C_{load}^{Res} \times D^k(t)$ refers to expected load deviation, while C_{renew}^{Res} denotes the forecasting error for all the renewables.

3.6. Simulation settings

In this simulation the curtailment rate is evaluated for offshore wind in the coastal regions and onshore wind in the "Three North" regions. For these targeted provinces, potential curtailment rate is evaluated from the 0GW wind expansion to the fully utilization of the provincial wind potential. For the offshore wind provincial potential,

all the maritime area suitable for offshore wind development within provincial EEZ is selected. Locations considered here for offshore wind construction are limited within China's EEZ. Areas designated as either special maritime reserves or shipping lanes are excluded. Shipping lanes are estimated using SO2 emission data derived from the MERRA-2 dataset as a surrogate identification: 20% of the cell area is removed for locations defined as emitting SO2 at a rate higher than 10^{-11} kg $m^{-2}s^{-1}$. The utilization rate of 5MW per km^2 is adopted here. For onshore wind provincial potential, all the area suitable for onshore wind development within provincial border is aggregated. Areas covered by water or permanent ice, or identified as forest or urban, are excluded based on the geographical information. Landscape information is derived from the Goddard Earth Observing System Data Assimilation System, with a geographical resolution of 6 km for mid-latitude regions. Areas with slopes higher than 10% are also excluded here.

During the integration simulation, curtailment rates for offshore wind in the coastal regions and onshore wind in "Three North" regions are evaluated from 0% to 100% of the provincial potential, with 1% as the minimum step. Power system simulation is conducted 100 times to evaluate the potential curtailment at every wind investment level. In the 2020 system simulation, other generators as thermal units, nuclear plants, hydropower stations and solar PV are fixed as existing provincial installations in 2020. Interprovincial transmissions are also fixed as the existing projects.

This calculation is carried out on a 64 core 3.2GHz Alicloud server employing parallel computing strategy. Hourly unit commitment results of two major provinces for offshore wind development (Fujian and Jiangsu) during a representative week are presented in Fig S21. For the selected 168h time span, power output of all the generation categories at various offshore wind penetration levels is indicated with colored areas.

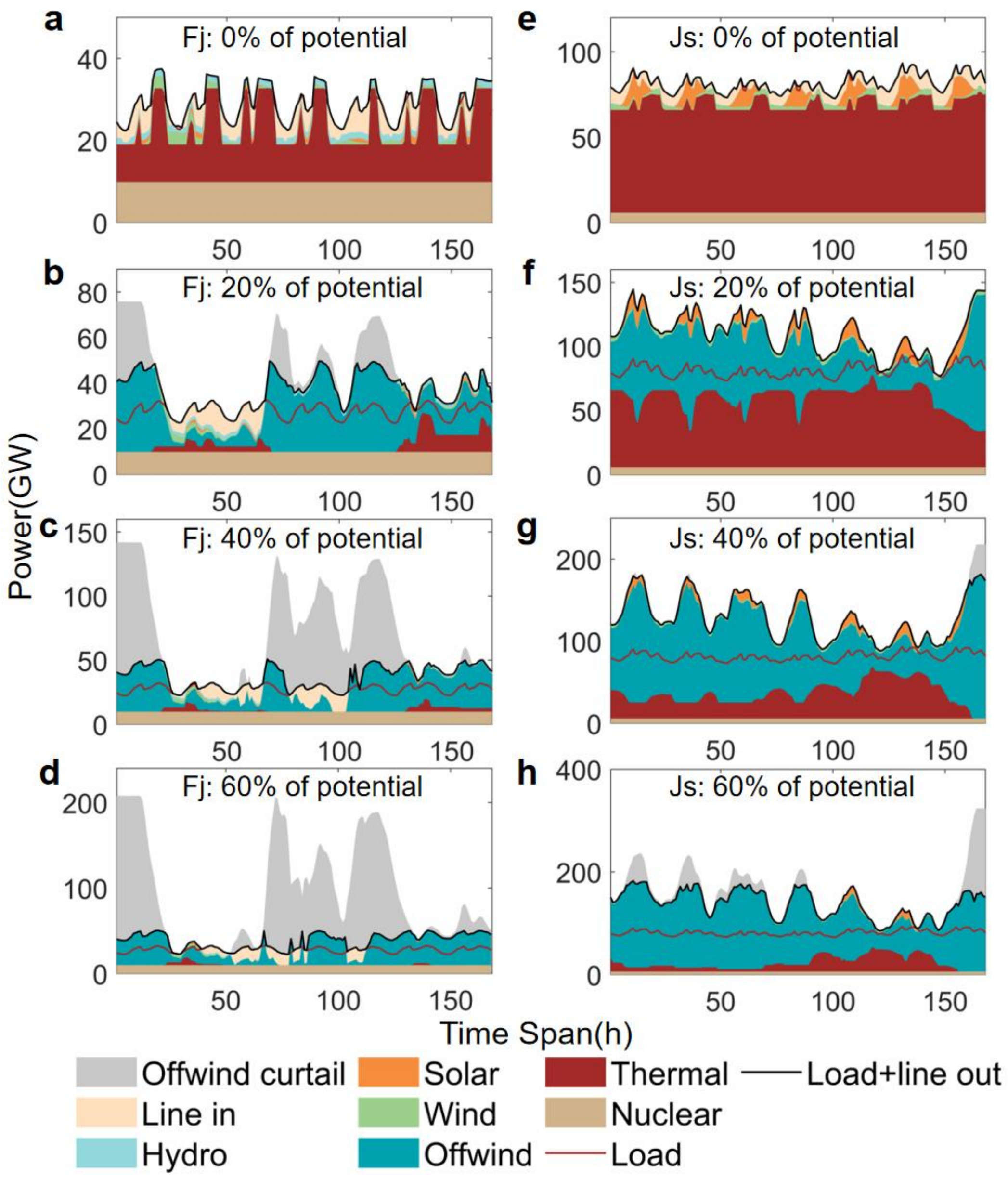

Fig S22: unit commitment results of offshore wind system integration simulation. Hourly power output of each generation category in Fujian (a-d) and Jiangsu (e-h), for various offshore wind penetration level at 0% (a,e), 20% (b,f), 40% (c,g) and 60% (d,h), respectively. Power output through inter-provincial transmissions is aggregated to the local load demand, illustrated with the black lines tagged "Load+ Line out". Offshore wind curtailment is presented with gray shadow areas above the load curve.

Supplied curves (maximum available wind capacity at different LCOE levels) of onshore wind in the "Three North" regions and offshore wind in the coastal region for 2020, 2030 and 2050 are presented in Fig S23.

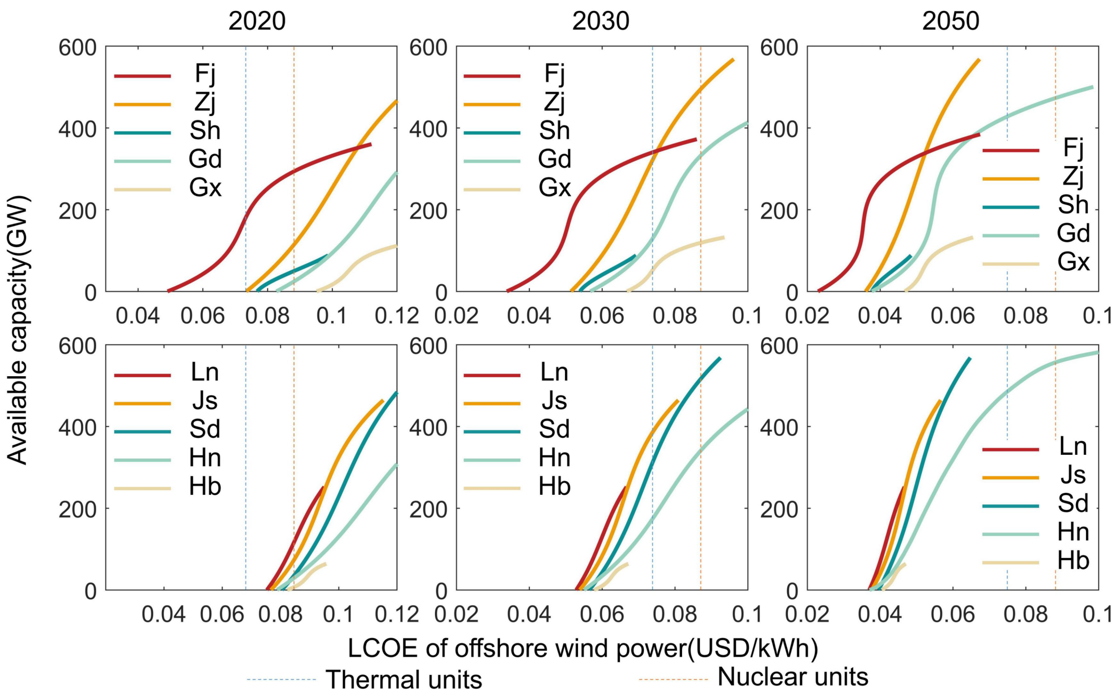

Fig. S23. Supply curves for onshore wind in "Three North" regions and offshore wind in coastal regions in 2020 (left), 2030 (mid) and 2050 (right). Levelized cost for benchmark coal-fire and nuclear power units equal 0.07 and 0.085 USD per kWh respectively, presented in blue and orange dashed lines.

4. Optimal investment model for 2030 and 2050

This investment model optimizes the allocation of all the non-hydro renewables (onshore, offshore wind and solar PV), thermal units, transmissions, storages and P2G facilities. A diagram of the optimal investment model is presented in the Fig S24.

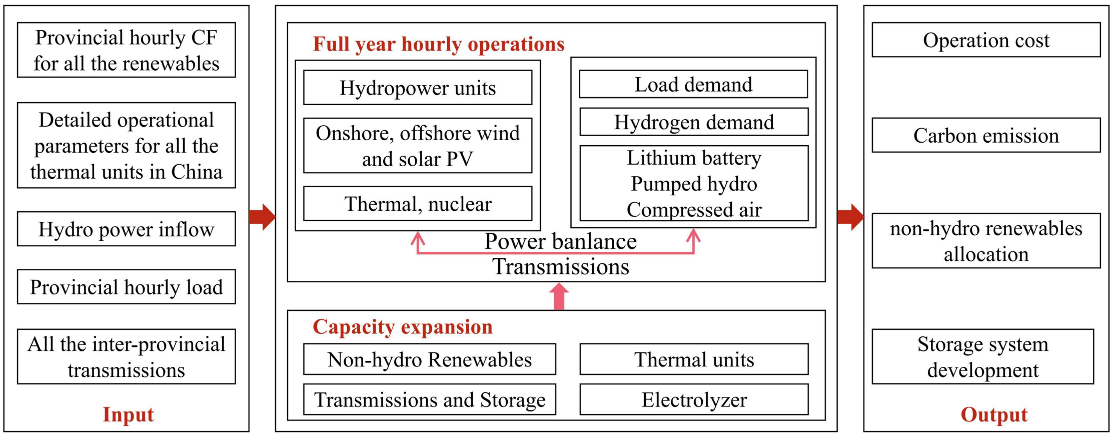

Fig S24: overall diagram of the optimal investment model.

4.1. Provincial load demand

The provincial load demand at hourly basis for 2030 and 2050 is scaled-up from the 2020 load curves with the algorithm presented in (53). r1-r3 denote the annual growth rates at different periods. This growth rate is provided by the State Grid Corporation of China (SGCC)[39], considering the general rules of population growth and economy development. $r1_k$ denotes the annual load growth rate for the province k from 2020 to 2025, ranging from 4.10% (in Shanghai) to 11.60% (in Xinjiang). For the years from 2025 to 2030, a unified annual load growth rate (r2) of 3.5% is applied to all the provinces. For the years from 2030 to 2050, the unified annual load growth rate (r3) falls to 1% for all the provincial regions.

$$D_{2030}^{k}(t) = D_{2020}^{k}(t) \times (1 + r1_k)^5 \times (1 + r2)^5 \tag{55}$$

$$D_{2050}^{k}(t) = D_{2020}^{k}(t) \times (1 + r1_k)^5 \times (1 + r2)^5 \times (1 + r3)^{20} \tag{56}$$

$D_{2020}^{k}(t)$, $D_{2030}^{k}(t)$ and $D_{2050}^{k}(t)$ denote the hourly load demand time series for the province k in 2020, 2030 and 2050 respectively.

4.2. Decision variable

This optimal investment model determines both the investment decisions and the operational decisions in a single optimization without iteration. Investment decisions for 2030 include the newly built capacities for all the non-hydro renewables (onshore, offshore wind and solar PV), thermal units and storage systems. While the investment decisions for 2050 include the newly deployed capacities for all the non-hydro renewables (onshore, offshore wind and solar PV), thermal units, inter-provincial transmissions, storage systems and P2G facilities. Investment variables for 2030 and 2050 optimal are presented as below.

Table S7: investment decisions for 2030

Decisions	Meaning
$\mathrm{Exp}_{\mathrm{on}}^{k}$	Onshore wind investment in province k
$\mathrm{Exp}_{\mathrm{so}}^{k}$	Solar PV investment in province k
$\mathrm{Exp}_{\mathrm{off}}^{k}$	Offshore wind investment in province k
$\mathrm{Exp}_{\mathrm{thermal}}^{j,k}$	Investment of thermal cluster j in province k
$\mathrm{Exp}_{\mathrm{store-P}}^{j,k}$	Power facility expansion of jth storage system in province k
$\mathrm{Exp}_{\mathrm{store-E}}^{j,k}$	Energy facility expansion of jth storage system in province k

Table S8: investment decisions for 2050

Decisions	Meaning
$\mathrm{Exp}_{\mathrm{on}}^{k}$	Onshore wind investment in province k
$\mathrm{Exp}_{\mathrm{so}}^{k}$	Solar PV investment in province k
$\mathrm{Exp}_{\mathrm{off}}^{k}$	Offshore wind investment in province k
$\mathrm{Exp}_{\mathrm{thermal}}^{j,k}$	Investment of thermal cluster j in province k
$\mathrm{Exp}_{\mathrm{store-P}}^{j,k}$	Power facility expansion of jth storage system in province k
$\mathrm{Exp}_{\mathrm{store-E}}^{j,k}$	Energy facility expansion of jth storage system in province k
$\mathrm{Exp}_{\mathrm{h2}}^{j,k}$	Capacity deployment of jth hydrogen electrolyzer in province k
$\mathrm{Exp}_{\mathrm{AC}}^{k,l}$	Capacity expansion of AC transmissions between province k and province l
$\mathrm{Exp}_{\mathrm{DC}}^{k,l}$	Capacity expansion of DC transmissions from province k to province l

Notably, inter-provincial AC corridor is freely dispatched at hourly basis, allowing for the bi-directional power flows between connected provinces. While DC corridor is dispatched adopting fixed strategy, only allowing for uni-directional power flow. Its utilization rate remains 90% during the day and falls to 50% throughout the night.

Therefore the $Exp_{DC}^{k,l}$ and $Exp_{DC}^{l,k}$ denote different investment decisions.

Operational decisions account for the committed and dispatched status for all the generation categories, the operation details for inter-provincial transmissions, storage systems and P2G facilities. Capacity available during the dispatch phases is bounded below the summary of initial capacity and newly invested capacity.

4.3. Objective function

The objective of the optimal investment model is to minimize the overall system costs, including (1) system investment costs: amortized capital expenditure of all the newly-deployed system facilities; and (2) system operational costs: start-up costs and normal operational costs for thermal units, fuel costs for nuclear plants, and the O&M expenses for each generation category.

Offshore wind cost estimation model (mentioned in Section 1) is employed here to calculate offshore wind unit investment cost for all the coastal provinces. Unit capital expenditure for all possible locations suitable for offshore wind development within provincial EEZ is calculated. Investment cost for onshore wind is derived from the provincial averaged project costs released by NDRC. Investment cost for the solar PV is derived from the IRENA Renewable Cost Database.

4.3.1. Investment cost

The system investment cost is formulated as below:

$$CapExp = \sum_{k=1}^{Nreg} (Exp_{on}^{k} \times W_{on}^{k} + Exp_{off}^{k} \times W_{off}^{k} + Exp_{solar}^{k} \times W_{solar}^{k}) + \sum_{k=1}^{N_{reg}} \sum_{l=1}^{N_{reg(l \neq k)}} Exp_{DC}^{k,l} \times W_{DC}^{k,l} + \sum_{k=1}^{N_{reg}} \sum_{l=k+1}^{N_{reg}} Exp_{AC}^{k,l} \times W_{AC}^{k,l} + \sum_{k=1}^{Nreg} \sum_{j=1}^{N_{storage}^{k}} (Exp_{store-P}^{j,k} \times W_{store-P}^{j,k} + Exp_{store-E}^{j,k} \times W_{store-E}^{j,k}) + \sum_{k=1}^{Nreg} \sum_{j=1}^{N_{thermal}^{k}} (Exp_{thermal}^{j,k} \times W_{thermal}^{j,k}) + \sum_{k=1}^{Nreg} \sum_{j=1}^{N_{H2}^{k}} (Exp_{H2}^{j,k} \times W_{H2}^{j,k}) \quad (57)$$

Where W_{on}^{k}, W_{off}^{k} and W_{solar}^{k} denotes the unit investment cost (at per kilowatt basis) for onshore, offshore wind and solar PV in province k. $W_{AC}^{k,l}$ denotes the unit investment cost for AC transmission corridor between province k and province l. $W_{DC}^{k,l}$ denotes the unit investment cost for DC transmission line from province k to province l. $W_{thermal}^{j,k}$ denotes unit investment cost for the jth thermal power cluster in province k. $W_{H2}^{j,k}$ denotes unit investment cost for the jth hydrogen electrolyzer in province k. N_{store}^{k} denotes the number of storage categories considered in province k. For the jth storage category in province k, $W_{store-P}^{j,k}$ and $W_{store-E}^{j,k}$ denotes the costs for power and energy specific facilities at per kilowatt basis.

4.3.2. Operational cost

The operational cost is formulated as below:

$$Oper = \sum_{t=1}^{T} \sum_{k=1}^{N_{reg}} \sum_{j=1}^{N_{th}^{k}} \left(C_{th}^{j,k}(t) + U_{th}^{j,k}(t) + D_{th}^{j,k}(t)\right) + \sum_{k=1}^{N_{reg}} OM^{k} \quad (58)$$

N_{th}^{k} denotes the number of thermal clusters in province k, taken as 12 for provinces with heating demand and 6 for the other. N_{reg} denotes the number of provinces considered in the simulation, taken as 29. T denotes the number of time intervals considered in the unit commitment simulation, taken as 8760 for the hourly simulation throughout a year. For the jth thermal cluster in province k, $C_{th}^{j,k}(t)$, $U_{th}^{j,k}$

and $D_{th}^{j,k}$ denote the fuel cost for normal generation, start up and shut down operations, respectively. A linear coal consumption relationship is adopted here, employing continuous variables to specify the fuel cost for each thermal cluster[30].

$$\begin{cases} C_{th}^{j,k}(t) = A_{th}^{j,k} \times X_0^{j,k}(t) \\ U_{th}^{j,k} = K_{th-up}^{j,k} \times X_2^{j,k}(t) \\ D_{th}^{j,k} = K_{th-down}^{j,k} \times X_3^{j,k}(t) \end{cases} \quad (59)$$

For the jth thermal cluster at province k, $A_{th}^{j,k}$ denotes the fuel cost at per kWh power generation basis. The fuel consumption relationship of coal, gas, CHP coal, and CHP gas refers to the research proposed by Xinyu Chen et al[31] and Haiwang Zhong et al[32]. Fuel costs for coal and natural gas are also determined at a provincial level referring to the statistical data proposed by IMCEC[33] and NDRC[34]. $K_{th-up}^{j,k}$ and $K_{th-down}^{j,k}$ define the fuel costs for startup and shut down capacity at per kWh basis[35]. For each thermal cluster, above parameters are determined as the capacity-weighted average value of all the clustered thermal units.

OM denotes the operation and maintenance costs for all the generation categories. This cost is taken as 15% of the amortized investment cost for onshore wind power[36], 20% for offshore wind power, 5% for solar PV[37], 20% to 30% for thermal units.

4.4. Flexibility constraints

Operational characteristics for the thermal group are described by the flexibility constraints, including (a) ramping limits: power output of thermal units cannot change suddenly, which is formulated in (60); (b) minimum online-offline time requirements: thermal unit cannot be shut down until it has been opened up for designated time, and cannot be opened up until it has been shut down for designated time, as (61)-(62); (c) minimum load levels: minimum power output for the opened up thermal units, which is formulated as (63); (d) must run units: certain units must be online during certain time periods, which is formulated as (64).

$$X_0^{j,k}(t) - X_0^{j,k}(t-1) \le \underline{A}_j X_2^{j,k}(t) - \underline{A}_j X_3^{j,k}(t) + R_U^{j,k}(X_1^{j,k}(t) - X_2^{j,k}(t) - X_3^{j,k}(t+1))$$

$$X_0^{j,k}(t) - X_0^{j,k}(t-1) \ge \underline{A}_j X_2^{j,k}(t) - \underline{A}_j X_3^{j,k}(t) - R_U^{j,k}(X_1^{j,k}(t) - X_2^{j,k}(t) - X_3^{j,k}(t-1)) \quad (60)$$

$$X_0^{j,k}(t) \le \overline{A}_j(X_1^{j,k}(t) - X_2^{j,k}(t) - X_3^{j,k}(t+1)) + \underline{A}_j(t) X_2^{j,k}(t) + \underline{A}_j X_3^{j,k}(t+1)$$

$$0 \le X_3^{j,k}(t+1) \le X_1^{j,k}(t) - \sum_{\tau=0}^{t-1} X_2^{j,k}(t-\tau) \; 1 \le t \le T_j^U - 1$$

$$0 \le X_3^{j,k}(1) \le X_1^{j,k}(0) \quad (61)$$

$$0 \le X_3^{j,k}(t+1) \le X_1^{j,k}(t) - \sum_{\tau=0}^{T_j^U-2} X_2^{j,k}(t-\tau) \; T_j^U \le t \le T-1$$

$$0 \le X_2^{j,k}(t+1) \le X^{j,k} - X_1^{j,k}(t) - \sum_{\tau=0}^{t-1} X_3^{j,k}(t-\tau) \; 1 \le t \le T_j^D - 1$$

$$0 \le X_2^{j,k}(1) \le X^{j,k} - X_1^{j,k}(0) \quad (62)$$

$$0 \le X_3^{j,k}(t+1) \le X^{j,k} - X_1^{j,k}(t) - \sum_{\tau=0}^{T_j^D-2} X_3^{j,k}(t-\tau) \; T_j^D \le t \le T-1$$

$$\underline{A}_j(t) \times X_1^j(t) \le X_0^j(t) \le \overline{A}_j(t) \times X_1^j(t) \quad (63)$$

$$X_1^{jc,k}(t) = X^{jc,k}(t \in t_h)$$
$$\left|X_0^{jc,k}(t) - X_0^{jc,k}(t-1)\right| \le R^{jc,k}(t \in t_h) \quad (64)$$

4.5. Hydropower model

The hydropower model is formulated the same way as the system operation model, including the balancing constraint for hydropower operations (65), the restriction of reservoir stage at initial and terminal timepoints (66), the limitation of hydropower output (66), and the definition of hydropower reserves (67).

$$\begin{cases} V^j(t) = V^j(t-1) + \frac{P_{hydro}^{j-1}(t-\tau)}{\eta^{j-1} \times H^{j-1}} + \\ S^{j-1}(t-\tau) - \frac{G^j(t)}{\eta^j \times H^j} - S^j(t) \\ V^1(t) = V^1(t-1) + I(t) - \frac{P_{hydro}^1(t)}{H^1} - S^1(t) \end{cases} \quad (65)$$

$$\begin{cases} 0 \le R^j \le R^j_{max} \\ 0 \le G^j \le G^j_{max} \\ R^j(t_{ini\&final}) = 0.8 \times R^j_{max} \end{cases} \tag{66}$$

$$\begin{cases} Res^j_{hy}(t) \le P^j_{hy-max} - P^j_{hy}(t) \\ V^j(t) - \frac{Res^j_{hy}(t)+P^j_{hy-max}}{\eta^j \times H^j} - S^j(t) \ge 0 \end{cases} \tag{67}$$

4.6. Transmission line model

The optimal deployment of transmission lines is different for 2030 and 2050.

For the 2030 investment, interprovincial transmissions account for all existing and recently approved or government planned transmission projects. Recently approved or government planned projects are presented in Table S9 and Table S10. Additional expansion for the inter-provincial transmission projects is not considered here, given the approval and construction process for transmission projects require long time period.

For the 2050 investment, all the transmission projects in 2030 are considered, and the optimal investment of inter-provincial transmissions is also allowed. Over 600 AC and DC transmission options between different provinces at different voltage levels are identified as technically feasible and included in our investment. The transmission options included in the investment model are presented as the gray lines in Fig S25. Transmission available for scheduling during the dispatch phases is bounded within the initial and newly installed capacity, indicated as:

$$\begin{cases} -\left(P^{k,l}_{AC-ini} + P^{k,l}_{AC-inv}\right) \le P^{k,l}_{AC}(t) \le P^{k,l}_{AC-ini} + P^{k,l}_{AC-inv} \\ 0 \le P^{k,l}_{DC}(t) \le P^{k,l}_{DC-ini} + P^{k,l}_{DC-inv} \end{cases} \tag{68}$$

$P^{k,l}_{AC-ini}$ and $P^{k,l}_{AC-inv}$ denote the initial and newly installed inter-provincial AC transmissions between province k and province l. $P^{k,l}_{DC-ini}$ and $P^{k,l}_{DC-inv}$ denote the initial and newly installed DC transmission lines from province k to province l. Negative bound for AC transmissions limits the counter flow from province l to province k.

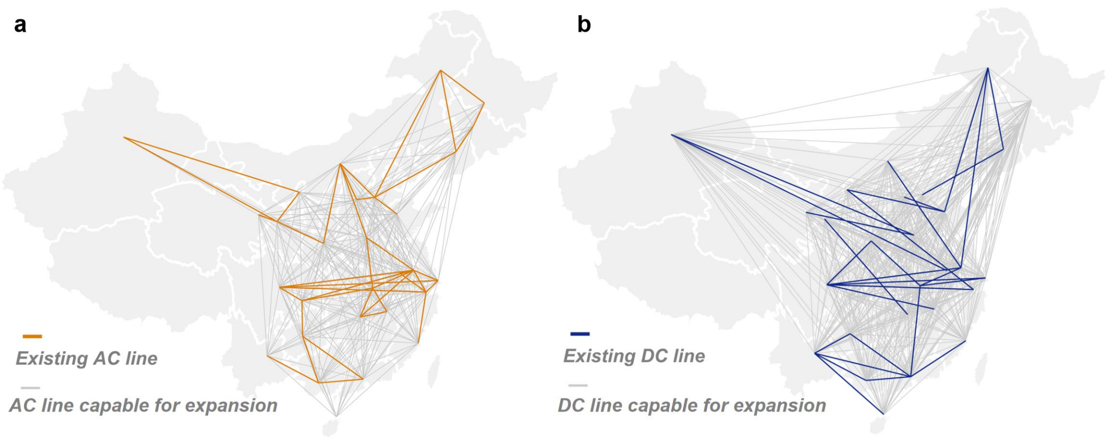

Fig S25: Existing inter-provincial transmissions, and transmission options capable for expansion. (a) Existing inter-provincial AC transmissions (indicated by orange lines), and alternative AC transmissions capable for the expansion (indicated by gray lines). **(b)** Existing inter-provincial DC transmissions (indicated by blue lines), and alternative DC transmissions capable for the expansion (indicated by the gray lines). Notably, the DC option corresponds to broader applicable scenarios, given the farther transmission distance.

Table S9. Recently approved or government planned DC transmissions by 2020Q4. Reference: [43-52]

DC projects	Passing provinces	Cap	Remarks
Sichuan Yazhong — Jiangxi ± 800kV	(1) Sichuan (2) Yunnan (3) Guizhou (4) Hunan (5) Jiangxi	8000	Southwest hydropower delivery
Sichuan Baihetan — Jiangsu ± 800kV	(1) Sichuan (2) Chongqing (3) Hubei (4) Anhui (5) Jiangsu	8000	Southwest hydropower delivery
Sichuan Baihetan — Zhejiang ± 800kV	(1) Sichuan (2) Chongqing (3) Hubei (4) Anhui (5) Zhejiang	8000	Southwest hydropower delivery
Sichuan Jinshang — Hubei ± 800kV	(1) Sichuan (2) Chongqing (3) Guizhou (4) Hunan (5) Hubei	8000	Southwest hydropower delivery
Yulin Shaanxi — Wuhan Hubei ± 800kV	(1) Shaanxi (2) Shanxi (3) Henan (4) Hubei	8000	Three north onshore wind delivery
Gansu — Shandong ± 800kV	(1) Gansu (2) Shaanxi (3) Shanxi (4) Henan (5) Shandong	8000	Three north onshore wind delivery
Xinjiang — Chongqing ± 800kV	(1) Xinjiang (2) Gansu (3) Sichuan (4) Chongqing	8000	Northern PV delivery

Table S10. Recently approved or government planned AC transmissions by 2020Q4. Reference: [43-52]

Transmission line	Cap	Route provinces	Remark
Zhumadian Wuhan	16000	Henan Hubei	East China Loop
Wuhan Nanchang	6000	Hubei Jiangxi	East China Loop
Nanchang Changsha	6000	Jiangxi Hunan	East China Loop
Nanyang Jingmen Changsha	16000	Henan Hubei Hunan	East China Loop
Zhumadian Nanyang	10000	Inner-Provincial	East China Loop
Wuhan Jingmen	6000	Inner-Provincial	East China Loop
Jinan Zaozhuang Xuzhou Nanjing	9800	Shandong Jiangsu	Sanhua Networking
Linyi Lianyungang Taizhou	8000	Shandong Jiangsu	Sanhua Networking
Zhumadian Huainan	8000	Henan Anhui	Sanhua Networking
Wuhan Southern Anhui	8000	Hubei Anhui	Sanhua Networking
Nanchang southern Zhejiang	8000	Jiangxi Zhejiang	Sanhua Networking
Ganzi Tianfu South	16000	Inner-Provincial	Chuan-Yu Networking
Aba Chengdu East	10000	Inner-Provincial	Chuan-Yu Networking
Tianfu South Tongliang	8000	Chongqing Sichuan	Chuan-Yu Networking
Zhangbei Shengli	8000	Mengdong Shijiazhuang	Three north on-wind

4.7. Energy storage model

Storage system in our model incorporates lithium battery for the short-term storage

options, pumped hydro (PHS) and compressed air (CAES) for the long-term storage options. Investment cost for the storage system is divided into the energy and power specific items. Energy specific investment determines the utmost energy storage capacity, corresponding to the electric cells for lithium battery, hydro reservoirs for PHS, and underground caverns for CAES. While power specific investment determines the peak power in-output, corresponding to the power electronic converters for lithium battery, electric machinery for PHS and compressors for CAES. A linear simulation model for the energy storage system is presented here, incorporating multiple operation details as the charge-discharge losses, self-discharge losses, annual throughput limitation and cycle periods. Functions for both power balance and backup supply are considered in our model.

Having considered existing pumped hydro within national jurisdiction, capacity available during dispatch is bounded within initial and newly deployed installations.

$$\begin{cases} Inv_{store-E}^{j,k} \geq 0 \;\; Inv_{store-P}^{j,k} \geq 0 \\ P_{cha}^{j,k}(t) \leq \left(Cap_{store-P}^{j,k} + Inv_{store-p}^{j,k}\right) \\ P_{dis}^{j,k}(t) \leq \left(Cap_{store-P}^{j,k} + Inv_{store-p}^{j,k}\right) \end{cases} \tag{69}$$

For the jth storage category in province k, $Cap_{store-E}^{j,k}$ and $Cap_{store-P}^{j,k}$ denote the initial capacity for energy and power specific facilities. $Inv_{store-E}^{j,k}$ and $Inv_{store-E}^{j,k}$ denote the newly deployed capacity specific to energy and power facilities. $P_{cha}^{j,k}(t)$ and $P_{dis}^{j,k}(t)$ denote the charge and discharge power at time t. Having considered charge-discharge losses and self-discharge losses, power balance for energy storage facility could be formulated as:

$$\begin{cases} E_{store}^{j,k}(t+1) = E_{store}^{j,k}(t) + R_{cha}^{j,k} \times P_{sto-cha}^{j,k}(t) \\ \quad - R_{dis}^{j,k} \times P_{sto-dis}^{j,k}(t) - R_{self}^{j,k} \times E_{store}^{j,k}(t) \\ E_{store}^{j,k}(1) = \left(Cap_{store-E}^{j,k} + Inv_{store}^{j,k}\right) \times R_{ini-E} \end{cases} \tag{70}$$

$E_{store}^{j,k}(t)$ denotes the energy level of jth storage category in province k at time t. R_{Ini_E} denotes the initial energy level and taken as 80% in our simulation. $R_{cha}^{j,k}$, $R_{dis}^{j,k}$ and $R_{self}^{j,k}$ denote the charge, discharge efficiencies and self-discharge rate for jth storage

device in province k. Energy level is also restricted by the min/max change rate:

$$\left(Cap_{store-E}^{j,k} + Inv_{store}^{j,k}\right) \times \underline{\mu}^{j,k} \leq E_{store}^{j,k}(t) \leq \left(Cap_{store-E}^{j,k} + Inv_{store}^{j,k}\right) \times \overline{\mu}^{j,k} \quad (71)$$

Where $\underline{\mu_s^{j,k}}$ and $\overline{\mu_s^{j,k}}$ denote the min-max charging rate of the jth storage system in province k, respectively. The maximum charge rate is evaluated 100% in our model. For certain types of storage systems, deep discharge will significantly reduce the expected lifetimes, and a minimum level is required.

Storage systems could potentially provide spinning reserve. Capabilities for provision of spinning reserve are constrained both by the total power rating and the energy remaining in the storage system at time t:

$$\begin{cases} Res_{store}^{j,k} + P_{sto-dis}^{j,k}(t) \leq Cap_{store-P}^{j,k} + Inv_{store-P}^{j,k} \\ 0 \leq E_{store}^{j,k}(t) - R_{dis}^{j,k} \times \left(Res_{store}^{j,k} + P_{sto-dis}^{j,k}(t)\right) - R_{self}^{j,k} \times E_{store}^{j,k}(t) \end{cases} \quad (72)$$

For the lithium battery, maximum watt-hours (Wh) that could pass through during the entire lifetime are provided by the battery manufacturers. On an annual basis, this could be expressed as:

$$\sum_{t=1}^{N} \left(P_{sto-cha}^{j,k}(t) + P_{sto-dis}^{j,k}(t)\right) \leq R_{Wh-Thr}^{j,k} \times \left(Cap_{store-E}^{j,k} + Inv_{store}^{j,k}\right) \quad (73)$$

where $R_{Wh\_Thr}^{j,k}$ is the amortized wh-throughput for the jth category of energy storage.

4.7.1. Compressed air storage system

Deployment of the compressed air storage system (CAES system hereafter) depends heavily on the local geographical conditions. Current technology usually employs salt cavern or coal mine for the underground air storage. Two existing commercial CAES plants, the Huntorf plant and the McIntosh plant, both employ underground salt caverns for the air storage. Main parameters for all major salt caverns in China are collected in this study, incorporating depth range, annual mining amount and the potential reserve. Available caverns for CAES projects are filtered by the depth range. The potential storage volume is evaluated based on the annual mining process, and further bounded by the potential reserve. Detailed data of major salt caverns in China is presented in Figure S26 and Table S11.

Table S11. Technical parameter of compressed air energy storage (CAES). The CAES system will transform to the adiabatic CAES technology 10-15 years from now. The adiabatic CAES system has a much higher efficiency (over 70%) but higher costs. Therefore, a stepwise increase in efficiency and capital expenditure will exist in 2040.

Item	2020	2030	2040	2050
M€2015 per MWh	0.65	0.65	1.0	0.8
-Energy (M€/MWh)	0.002	0.002	0.002	0.002
-Capacity (M€/MW)	0.6	0.6	0.9	0.9
-Other costs (M€/MWh)	0.085	0.085	0.085	0.085
Fixed O&M (€2016/MW/year)	2460	2460	2460	2460
Variable O&M (€2016/MWh)	2.46	2.46	2.46	2.46
Round trip efficiency (%)	55	60	70	72
- Charge efficiency (%)	80	80	84	85
- Discharge efficiency (%)	69	80	84	85
Energy losses(%/period)	0	0	0	0
Forced outage (%)	5	5	4	4
Planned outage (weeks/y)	5	5	4	3
Technical lifetime (years)	40	40	40	40
Construction time (years)	<3	<3	<3	<3

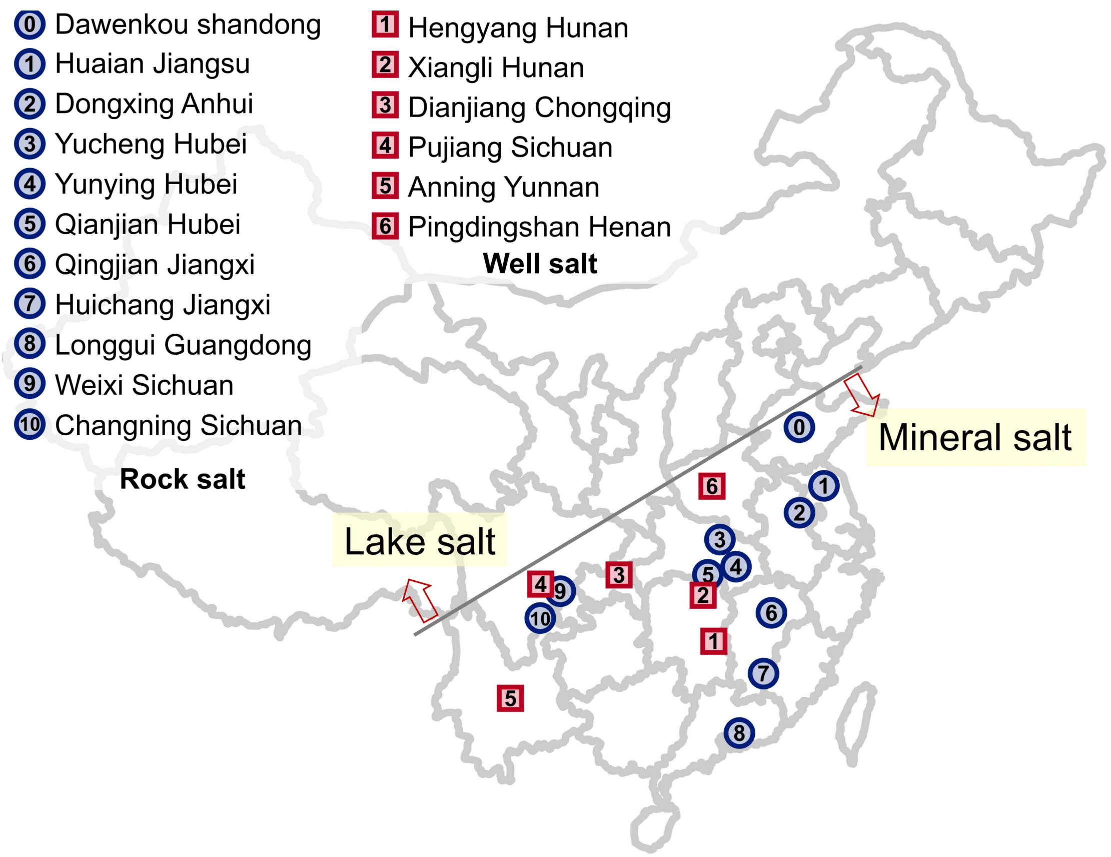

Fig S26. Spatial distributions of all major salt caverns in mainland China, including caverns for the rock salt and well salt. Salt caverns mainly distribute in East China, Center China and South China.

Table S12. Detailed information of all major salt caverns in China.

Index	Name	Storage	Annual mining	Depth
		10^8 ton	10^4 ton	m
(0)	Dawenkou	75	50	800-1000
(1)	Huaian	2500	75	630-1825
(2)	Dingyuan	7	10	220-595
(3)	Yucheng	-	-	-
(4)	Yunmeng	280	130	300-850
(5)	Qianjiang	50	-	700-2145
(6)	Qingjiang	95	100	595-1170
(7)	Huichang	-	-	800-1200
(8)	Longgui	0.6	55	480-640
(9)	Weixi	90	2295	800-1800
(10)	Changning	4.3	60	1885-2935
[1]	Hengyang	17	45	210-395
[2]	Lixian	1	30	220-500
[3]	Dianjiang	82.22	-	3400

[4]	Pujiang	-	-	>3300
[5]	Anning	135	-	125-900
[6]	Pingdingshan	-	200	-

Salt caverns listed above are filtered by the buried depth and mining body thickness. The filtering criteria are listed in Table S13. Salt caverns in eastern, northern and center China are basically suitable for the deployment of CAES. But most of the salt caverns in Northwest China are limited by the buried depth.

Table S13. Filtering criteria of the available salt cavern.

Filtering criteria	Requirement
Buried depth	<1500m
Thickness of mining body	>100m

Available energy density for CAES projects is evaluated according to the practical project data in the European Union (listed in Table S14, including projects in operation and projects under construction). Energy density denotes the energy stored in per cubic meter of compressed air, and mainly determined by the pressure range. Energy density for all the CAES projects in EU are calculated and listed in the last column of Table S14.

Table S14. Main parameters of all the CAES projects in European Union. These data incorporate both the projects in operation and the projects under construction. P_min and P_max denote the minimum and maximum operating pressure during charge and discharge process. E denotes the total energy storage in the CAES project.

	Site name	Status	Volume	P_min	P_max	Energy	Energy
			$10^6 m^3$	$10^5 pa$	$10^5 pa$	GWh	$kWh{\cdot}m^{-3}$
1	Horsea	operate	1.98	120	270	42.855	21.645
2	Aldbrough I	operate	2.43	120	270	52.38	21.555
3	Whitehill	construct	2.5	100	345	91.665	36.665
4	Holford H165	operate	0.175	70	85	-	-
5	Hilltop Farm	operate	6.25	29	45	9.525	1.525
6	Holford	operate	2.9	40	105	23.81	8.21
7	Stublach	operate	6.6	30	101	52.38	7.935
8	King street	construct	5.5	33	66	19.05	3.465
9	Keuper	construct	5.97	43.8	123	58.335	9.77
10	Gateway	construct	20	36	120	200	10
11	Preesall	construct	6.8	33	92	45.24	6.655

12	Islandmagee	construct	3.36	120	250	64.285	19.13
13	Portland	construct	2	2	240	64.285	32.145

Five different pressure ranges (first line in Table S15) are selected to evaluate the available CAES potential in 2050. Pressure range of 120-250 Bar is selected in our model.

Table S15. Available CAES potential in 2050 for each province.

Province	Pressure range (Bar)				
	40–105	36–120	120–250	120–270	100–345
Shandong (Gwh)	60	75	145	160	275
Jiangsu (Gwh)	710	865	1660	1870	3180
Anhui (Gwh)	10	15	30	30	55
Jiangxi (Gwh)	125	150	285	325	550
Guangdong (Gwh)	70	85	160	180	300
Sichuan (Gwh)	2825	3445	6590	7420	12620
Hunan (Gwh)	90	115	215	240	410
Hubei (Gwh)	160	195	375	420	715
Henan (Gwh)	245	300	575	645	1100

4.7.2. Pumped hydro storage system

Deployment of pumped hydro storage system is also restricted by the geographical conditions. Selection of projects is based on the potential hydro head, geographical location, topography and geology, reservoir inundation conditions, environmental impact, engineering technology and economic competitiveness. The potential pumped hydro storage sites detected for each province is illustrated in Table 17. Investment of pumped hydro storage system is bounded below the provincial potential in our model.

Table S16. Provincial pumped hydro storage potential detected for each province. (Detailed project-level information is available from the lead contact on reasonable request)

Province	Potential (10^4kW)	Province	Potential (10^4kW)
National	67500	Hubei	3640
Hebei	1880	Hunan	3280
Shanxi	1500	Guangdong	3580
Inner Mongolia	1170	Guangxi	2280
Liaoning	1860	Hainan	820
Jilin	3230	Chongqing	840
Heilongjiang	4130	Sichuan	1460
Jiangsu	360	Guizhou	4080
Zhejiang	3800	Tibet	7195
Anhui	2180	Shaanxi	3555
Jiangxi	1300	Gansu	3350
Shandong	1360	Qinghai	4030
Henan	2370	Ningxia	780
		Xinjiang	3660

4.8. Power balance and reserve constraints

4.8.1. Power balance constraints

For each province at every time point, total power generation of each generation category should equal the summary of load demand and hydrogen generation demand, which could be formulated as:

$$\sum_{j=1}^{N_{ther}^{k}} X_0^{j,k}(t) + P_w^k(t) + P_{off}^k(t) + P_s^k(t) + P_{nu}^k(t) + P_{hy}^k(t) + P_{EX-AC}^k(t) + P_{EX-DC}^k(t) = D^k(t) + P_{H2}^{j,k}(t) \quad (74)$$

Among it:

$$\begin{cases} P_{EX-AC}^k(t) = \sum_{l=1(l\neq k)}^{N_{reg}} P_{AC}^{k,l}(t) \\ P_{EX-DC}^k(t) = \sum_{l=1(l\neq k)}^{N_{reg}} P_{DC}^{l,k}(t) - \sum_{l=1(l\neq k)}^{N_{reg}} P_{DC}^{k,l}(t) \end{cases} \quad (75)$$

Where $P_{EX-AC}^k(t)$ and $P_{EX-DC}^k(t)$ refer to the AC and DC power exchange between province k and other provinces. Power inflow is defined as positive, and outflow is defined as negative. $D^k(t)$ denotes the power demand of province k at time t, while

$P_{H2}^{j,k}(t)$ denotes the electricity consumption of jth electrolyzer in province k at time t. $P_{H2}^{j,k}(t)$ is set to zero in 2030 investment simulation.

4.8.2. Reserve constraints

$$\begin{cases} \sum_{j=1}^{N_{the}^{k}} \left(\overline{A}^{j,k} \times X_1^{j,k}(t) - X_0^{j,k}(t)\right) + C_w^{cre}\left(CF_w^k(t) \times Cap_w^k(t) - P_w^k(t)\right) + \\ C_{off}^{cre}\left(CF_{off}^k(t) \times Cap_{off}^k(t) - P_{off}^k(t)\right) + C_s^{cre}\left(CF_s^k(t) \times Cap_s^k(t) - P_s^k(t)\right) + \\ \sum_{j=1}^{N_{hy}^{k}} Res_{hy}^{j,k}(t) \le C_{load}^{Res} \times \left(D^k(t) + P_{H2}^{j,k}(t)\right) + C_{renew}^{Res} \times \left(P_w(t) + P_s(t) + P_{off}(t)\right) \end{cases} \quad (76)$$

where $\overline{A}^{j,k}$ denotes the maximum power generation ratio of jth thermal group in province k. $\overline{A}^{j,k} \times X_1^{j,k}(t) - X_0^{j,k}(t)$ is defined as the rotating backup (or hot-standby) of thermal group j. This value refers to the thermal capacity committed online but in the no-load state. Reserve capacity of renewables is defined as the deviation between the potential power generation and the actual grid-tied power output. Potential generation is determined by the real-time capacity factor and the renewable installation. Grid-tied output denotes the electricity injected to the power system. Reserve of renewables is further multiplied by a confidence factor to offset its unreliable output pattern. $Res_{hy}^{j,k}(t)$ denotes the backup provided by the j hydro power station in province k and is defined in equation (64). $D^k(t)$ refers to the power demand of province k at time t, and $P_{H2}^{k,n}(t)$ refers to the power input of type n hydrogen electrolyzer in the province k.

4.9. Settings for hydrogen economy

4.9.1. Hydrogen demand

National hydrogen demand is estimated to be 60 Mton in 2050[53], accounting for 10% of the terminal energy. Industry is the primary sector for hydrogen consumption, mainly employed as the basic raw material in chemical and refining sectors, and the reducing agent in steel sector. Remaining hydrogen demand is largely concentrated in the transportation sector, employed as the substitute fuel for heavy-duty trucks. In 2050, hydrogen demand as basis material remains unchanged with that in 2020. Remaining hydrogen demand is divided into each provincial region according to fixed proportion[53]. The annual steel production in 2020 and total amount of the road freight in 2020 is listed in Table S17. Obtained provincial hydrogen demand is illustrated in Fig S27. “Coastal” denotes the provincial regions directly adjacent to the maritime space. “Near Coastal” denotes provinces contiguous to the coastal provinces. “Inland” denotes other provinces. Coastal, near coastal and inland provinces account for 65%, 20% and 15% of the national hydrogen demand, respectively. Notably, national hydrogen demand mainly concentrates at the coastal provinces, indicating an extra demand for offshore wind capacity in future hydrogen economy.

Table S17. crude iron, crude steel, steel production and freight transportation on road of the 31 provincial regions in mainland China. Item steel production (4th column in Table S17) and freight transportation (5th column in Table S17) are employed here for the evaluation of provincial hydrogen demand[42].

Province	Crude Iron	Crude Steel	Steel Products	Freight Transport
	10^4 ton	10^4 ton	10^4 ton	10^4 ton
Beijing	0	0	184.40	21790
Tianjin	2198.85	2171.80	5724.05	32260
Hebei	22903.75	24976.95	31320.10	211940
Shanxi	6089.10	6637.80	6181.45	98205
IM	2380.85	3119.85	2883.90	109000
Liaoning	7235.20	7609.40	7578.40	138570
Jilin	1407.75	1525.60	1661.60	38275
Heilongjiang	863.15	986.55	878.95	35520
Shanghai	1411.35	1575.60	1879.60	46050
Jiangsu	10022.90	12108.20	15004.85	174625
Zhejiang	852.85	1457.05	3806.70	189580
Anhui	2537.30	3696.70	3607.45	243530
Fujian	1106.20	2466.50	3861.65	91135
Jiangxi	2332.05	2682.05	3093.90	141900
Shandong	7668.40	7993.50	11269.30	267230
Henan	2769.50	3530.15	4233.35	193630
Hubei	2727.45	3557.25	3649.10	114345
Hunan	2105.45	2612.90	2729.75	176440
Guangdong	2158.75	3382.35	4866.20	231170
Guangxi	1457.15	3452.25	4731.15	145325
Hainan	0	0	0	6855
Chongqing	637.85	899.95	1309.95	99680
Sichuan	2136.80	2792.65	3437.20	157600
Guizhou	368.65	461.95	741.10	79410
Yunnan	1873.25	2233.00	2640.70	115620
Tibet	0	0	0	4040
Shaanxi	1232.20	1521.55	2020.00	116055
Gansu	782.30	1059.15	1102.65	61270
Qinghai	160.35	193.25	189.10	10835
Ningxia	320.00	466.60	482.00	34215
Xinjiang	1158.30	1306.15	1420.50	40305

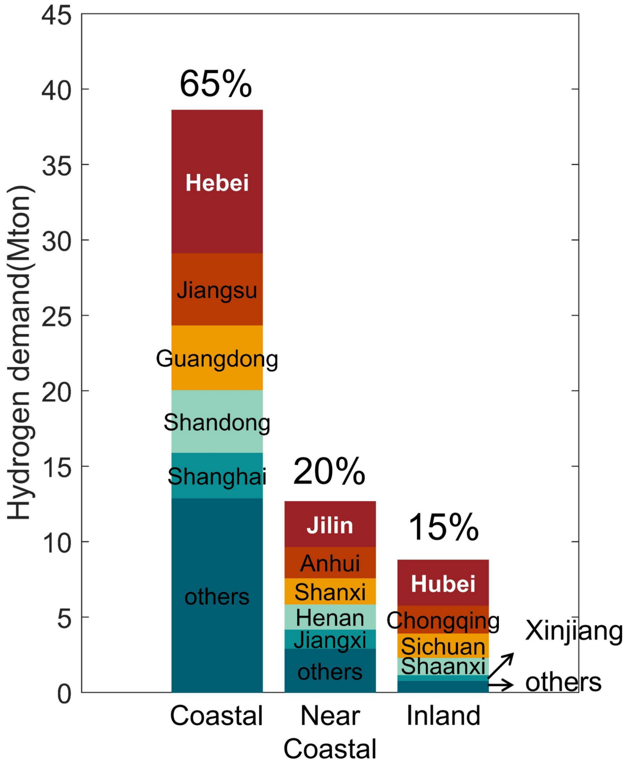

Fig S27: Hydrogen demand in 2050 for the coastal, near coastal and inland provinces. Coastal provinces denote the provincial regions directly adjacent to maritime space, as the Beijing, Tianjin, Hebei, Liaoning, Shandong, Jiangsu, Zhejiang, Shanghai, Fujian, Hainan, Guangdong, Guangxi. Near coastal provinces denote the provincial regions contiguous to coastal provinces, as the Shanxi, Inner Mongolia, Jilin, Anhui, Jiangxi, Henan, Hunan, Guizhou, Yunnan. Inland provinces denote other provincial regions, including the Hubei, Chongqing, Sichuan, Shanxi, Gansu, Qinghai, Ningxia, Xinjiang and Heilongjiang. For above three provincial classifications, the top 5 provinces for hydrogen demand are ranked in order, while the remaining provinces are aggregated to "others". Coastal, near coastal and inland provinces account for 65%, 20% and 15% of the national hydrogen demand, respectively.

4.9.2. Hydrogen generation

P2G infrastructure is deployed for each provincial region, incorporating different technologies as Alkaline electrolyzer (AEC), Solid oxide electrolyzer (SOEC) and the Proton exchange membrane electrolyzer (PEM). Technical parameters for each type of electrolyzer are indicated in the Table S18. Hydrogen generation per MWh_e input is evaluated as 22.5kg, 21.2kg and 25.1kg for the AEC, SOEC and PEM technology in 2050, assuming the electrolytic efficiency of 75%, 70.5% and 83.5% respectively[54].

Output pressure for electrolyzers is also considered, for its further impact on the

compression process. Output pressure usually remains 30 Bar for AEC technology, but could be as high as 50 Bar for the PEM equipment[54]. Notably, minimum online-offline times and ramping rates are not considered in this study— the superior flexibility of mainstream electrolyzers will not lead to a significant influence on the system operations[55]. Power input for electrolyzer participates in hourly power system balancing. Available power input during operation is bounded within the capacity investment.

Table S18. Technical parameters of each kind of electrolyzer.

	Item	2020	2030	2040	2050
Alkaline electrolyzer (AEC)	Efficiency	0.665	0.68	0.715	0.75
	ΔE from HHV to LHV	0.121	0.124	0.13	0.137
	Heat losses	0.214	0.196	0.155	0.113
	-recoverable heat loss	0.184	0.166	0.125	0.083
	€/kW of input	650	450	300	250
	Fixed O&M/year	0.02	0.02	0.02	0.02
	Variables O&M	-	-	-	-
	Lifetime	25y	30y	32y	35y
Solid oxide electrolyzer (SOEC)	Efficiency	0.58	0.655	0.68	0.705
	ΔE from HHV to LHV	0.106	0.119	0.124	0.128
	Heat losses	0.314	0.226	0.196	0.167
	-recoverable heat loss	0.284	0.196	0.166	0.137
	€/kW of input	925	650	450	400
	Fixed O&M	4	4	4	4
	Variables O&M	-	-	-	-
	Lifetime	20y	25y	28y	30y
Proton exchange membrane electrolyzer (PEM)	Efficiency	0.775	0.805	0.82	0.835
	ΔE from HHV to LHV	0.141	0.147	0.149	0.152
	Heat losses	0.084	0.048	0.031	0.013
	-recoverable heat loss	-	-	-	-
	€/kW of input	4490	1900	1340	785
	Fixed O&M	0.12	0.12	0.12	0.12
	Variables O&M	-	-	-	-
	Lifetime	10y	20y	20y	20y

*Due to the technical uncertainties, utilization of the recoverable heat loss is not considered in this study.

4.9.3. Hydrogen transportation

Inter-provincial hydrogen transportation considers (1) gaseous hydrogen: hydrogen is pressurized, pumped into the steel pipeline and transmitted underground; (2) liquid

hydrogen: hydrogen is liquefied, filled into the steel cylinder and delivered by trunk; (3) hydrogen carrier: hydrogen is transformed into hydrogen carrier, transported by trunk and dehydrogenized at the destination. Hydrogen carrier in this study includes NH3 (Ammonia), DBT (Di-benzyl toluene) and TOL (toluene)[56]. Basic information of above three transmission technologies are listed in Table S19[57]. Techno-economics of each organic hydrogen carrier is listed in Table S20[58].

Table S19: inter-provincial hydrogen transportation techniques considered in this study. Hydrogen transportation in solid state (as metal hydride) is not considered, for the uncertainty in future cost reduction. Organism carrier here includes the NH3 (Ammonia), DBT (Di-benzyl toluene) and TOL (Toluene).

Hydrogen state	Transportation	Pressure MP	Loading kg/truck	V-Density kg/m3	M-Density wt%
Gaseous state	Pipeline	-	-	-	-
Gaseous state	Tube trailer	20	300-400	14.5	1.1
Liquid state	Tank trailer	0.6	7000	64	14
Organism	Tank trailer	0.1	2000	40-50	4

Table S20. Techno-economic data for inter-provincial H2 transportation.

Pipeline [a]	Units	Value
Lifetime	year	40
Installed Capacity	$ktH2\cdot y^{-1}$	340
Gas density	$kg\cdot m^{-3}$	7.9
Gas Velocity	$m\cdot s^{-1}$	15
Cap_Exp	USD million$\cdot km^{-1}$	1.21
Annual OPEX	% of CapExp$\cdot y^{-1}$	-
**Liquefaction [b]**	**Units**	**Value**
Lifetime	year	30
Scale_Co	-	0.66
Installed capacity	$ktH2\cdot y^{-1}$	260
Electricity_Use	$kWh\cdot kgH2^{-1}$	6.1
Cap_Exp	USD million	1400
Annual OPEX	% of CapExp$\cdot y^{-1}$	0.04
**Tol-hy [c]**	**Units**	**Value**
Lifetime	year	30
Scale_Co	-	0.66
Installed capacity	ktTol per year	4 200
Electricity_Use	kWh per kgH2	1.5
Toluene_Cost	USD per tTol	400
Toluene_Use	ktTol per y	100
Cap_Exp	USD million	230
Annual OPEX	% of Cap_Exp$\cdot y^{-1}$	0.04
**Tol-De-hy [d]**	**Units**	**Value**
Lifetime	year	30
Scale_Co	-	0.66
Installed capacity	ktTol per year	4 200
Electricity_Use	kWh per kgH2	0.4
Heat required	kWh per kgH2	13.6
H2 purification [e]	kWh per kgH2	1.1
H2 recovery	%	90%×98%
Cap_Exp	USD million	670
Annual OPEX [f]	% of Cap_Exp	0.04
**NH3 [g]**	**Units**	**Value**
Electricity	USD per ton NH3	15
Cap_Exp	USD per ton NH3	60
**De-NH3 [h]**	**Units**	**Value**
Lifetime	year	30
Scale_Co	-	0.66

Installed cap	ktTol per year	1 500
Ele_Use	kWh per kgH2	-
Heat required	kWh per kgH2	9.7
H2 purification [i]	kWh per kgH2	1.5
H2 recovery	%	99%×85%
Cap_Exp	USD million	460
Annual OPEX	% of Cap_Exp	0.04

Notes:

[a] Pipeline denotes steel pipeline employed for the hydrogen underground transportation. Capital expenditure for the pipeline is assumed proportional to transmission distance.

[b] Liquefaction denotes the process where gaseous hydrogen is transformed to liquid hydrogen using liquefier. Scale_Co (Scale Coefficient) is a parameter to describe the economics of scale — increased investment scale usually leads to a decreased average cost. This phenomenon suits well for the integrated equipment (equipment that is not combined with multiple identical modules).

[c] Tol-hy denotes hydrogenation process from toluene (C7H8) to methyl cyclohexane (C7H14). Hydrogenation process is conducted in the reaction kettle with elevated temperature and pressure (200°C and 20 Bar). During this process hydrogen is covalently bounded to the organic carrier.

[d] De-Tol-hy refers to de-hydrogenation process from methyl cyclohexane (C7H14) to toluene (C7H8). During this process hydrogen in the loaded carrier gets separated by providing energy in the form of heat and in the presence of a catalyst. The unloaded Toluene is then stored for further cycling. Heat required for the de-hydrogenation process could be cost-competitively provided by industrial waste heat.

[e] Hydrogen obtained from the de-hydrogenation process demands for the further purification. Purification of obtained hydrogen (Pressure swing adsorption) consumes extra 1.1 kWh per kgH2.

[f] In the round-trip process (Tol-hy and De-Tol-hy), toluene will be gradually consumed owing to the volatilization and other losses during transformation. Toluene consumption for the listed liquefier (rated 4200 kt per year) is estimated to be 100 kt per year.

[g] NH3 denotes the transformation process from hydrogen to the ammonia. This process is conducted in the Haber-Bosch reactor, combined with the nitrogen separated from air and catalyst such as platinum rhodium alloy mesh.

[h] De-NH3 donates the de-hydrogenation process from the ammonia to hydrogen. Heat required in the de-hydrogenation process could also be provided with industrial waste heat. Purification of the obtained hydrogen (PSA) consumes 1.5kWh per kg H2.

[i] Notably, ammonia could be directly utilized as fuel (for ocean cargo ships) or the industrial feed stock (for fertilizer production). In these cases, the ammonia is not converted to the hydrogen. However, these cases are not incorporated in our study.

Based on the data in Table S20, total expenses in the conversion and de-conversion process for Tol (Toluene), DBT (Di-benzyl toluene), NH_3 (Ammonia) and LH_2 (liquid hydrogen) are presented in Fig S28. These expenses include both the capital expenditure and the energy expenses in the round-trip process. Capital expenditure refers to the amortized investment costs for the employed equipment (Liquefier and

Evaporator for LH_2, Reaction Kettle for Tol, DBT and NH_3). Energy expenses correspond to heat and electricity consumed during this process. The total expenses are converted to USD per kilogram hydrogen transportation, and further presented in Fig S28.

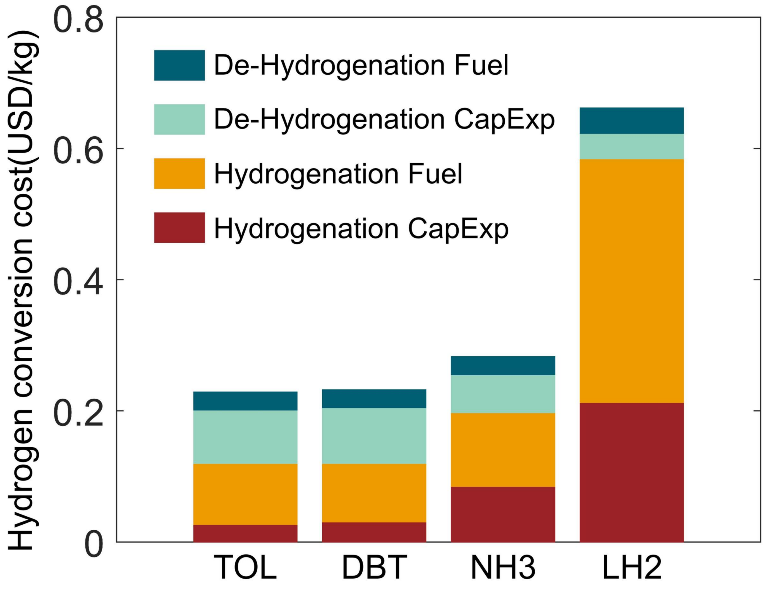

Fig S28. Hydrogenation and de-hydrogenation cost for the TOL (toluene), DBT (Di-benzyl toluene), NH3 (Ammonia) and LH2 (Liquid hydrogen). Capital expenditure denotes the amortized investment cost for the employed equipment. Fuel expenses denotes the costs for fossil-fuels and electricity consumed in related process. Cost for fossil-fuels and electricity is obtained from the estimation of IEA.

Table S21: Energy consumption in hydrogen compression.

St pressure(bar)	End pressure(bar)	Ideal power (GJ per tH2)	Practical power (GJ per tH2)
0	20	6.5	10
20	30	1.3	2
30	100	1.8	2.8
100 (PEM out)	350 (Store need)	4.8	7.4
150	700	3.6	5.5

Based on the evaluation of each hydrogen transmission technique, hydrogen delivery costs at per kilogram basis is presented for the distance from 50-3000 km in Fig S29. Pipeline is the most cost-competitive technology for inter-provincial hydrogen transmission. Transmission cost for the hydrogen pipeline is proportional to delivery distance. For organic hydrogen carrier as NH3 (Ammonia) and TOL (Toluene), the transmission cost is less sensitive to delivery distance. Organic

hydrogen carrier could be more cost-competitive for the long-distance hydrogen delivery.

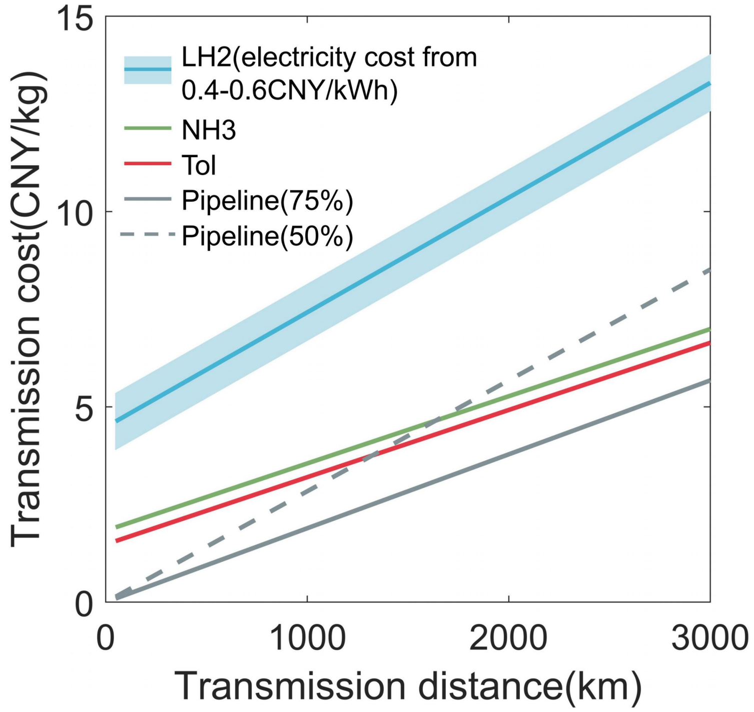

Fig S29. H2 transmission cost at different distance. Hydrogen transmission costs for pipeline (with a utilization rate of 75%), pipeline (with a utilization rate of 50%), ammonia, toluene, liquid hydrogen (with the electricity cost ranges from 0.4-0.6 CNY per kWh) are illustrated with the transmission distance from 50 to 3000km.

Hydrogen transportation cost in the power system simulation is calculated as the hydrogen transportation amount multiplies the unit transportation cost. Cost of hydrogen transmitted by pipeline between different provinces is illustrated in Fig S30.

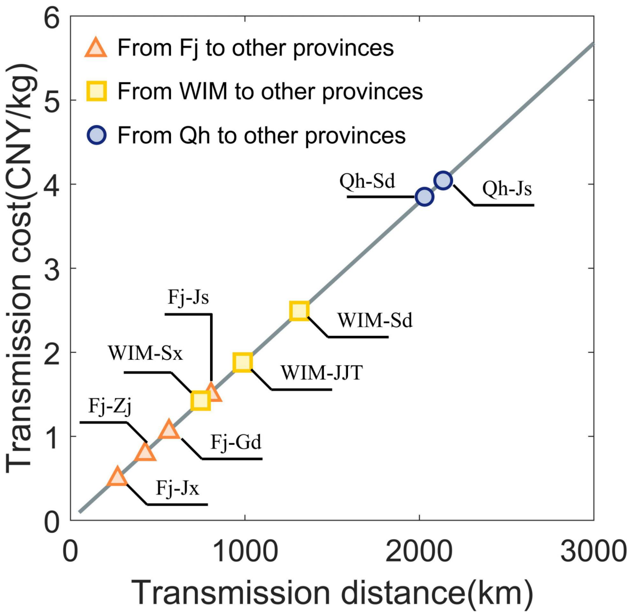

Fig S30. Cost of hydrogen transmitted by pipeline between different provinces. Fujian, West inner Mongolia and Qinghai are regarded with the best resources for offshore wind, onshore wind and solar PV. Fj denotes Fujian, Zj denotes Zhejiang, Jx denotes Jiangxi, Gd denotes Guangdong, WIM denotes the West inner Mongolia, Sx denotes Shanxi, Js denotes Jiangsu, JJT denotes Beijing-Tianjin-Hebei, Sd denotes Shandong, Qh denotes Qinghai.

4.10. Simulation settings

4.10.1. Scenario settings in 2030

Two scenarios are considered in the 2030 simulation: (1) Business as usual (BAU) fixes the capacity investment for non-hydro renewables according to government plan, while allows for freely expansion of thermal units and storage systems; (2) optimal planning strategy (Opt) optimizes the provincial investments for all the non-hydro renewables, thermal units and storage systems, to fulfill 40% renewable penetration target proposed by NEA.

Table S22. Scenario settings in 2030.

Scenario settings in 2030	BAU	Opt
Non-hydro renewables	According to government Planning by Feb 1st 2022	Freely optimized
Thermal units	Freely optimized	
Transmissions	Existing and lately planned transmissions by 2020Q4	
Storages	Freely optimized	
RPS target	None	40% penetration

At the end of 2020, Chinese president Xi JinPing announced a commitment for China to reach the 25% non-fossil energy consumption in the primary energy by 2030[59]. The National Energy Administration (NEA) also formulated a planning of 40% renewable penetration in the electricity sector (including electricity generation from hydropower station) by 2030[60,61]. Notably, above two plans are equivalent based on the real-world statistics data in the past ten years. The renewable penetration in electricity sector (RPE hereafter), and the non-fossil penetration in the primary energy consumption (RPP hereafter) from 2011 to 2019 are illustrated in Fig S31. Above two targets follow a rigorous linear relationship, with an R^2 up to 0.965. Based on the obtained fitting equation, 25% RPP corresponds to a RPE valued 38%, which is quite consistent with the 40% national target. The 40% renewable penetration rate in the electricity sector is therefore adopted in our 2030 system simulations.

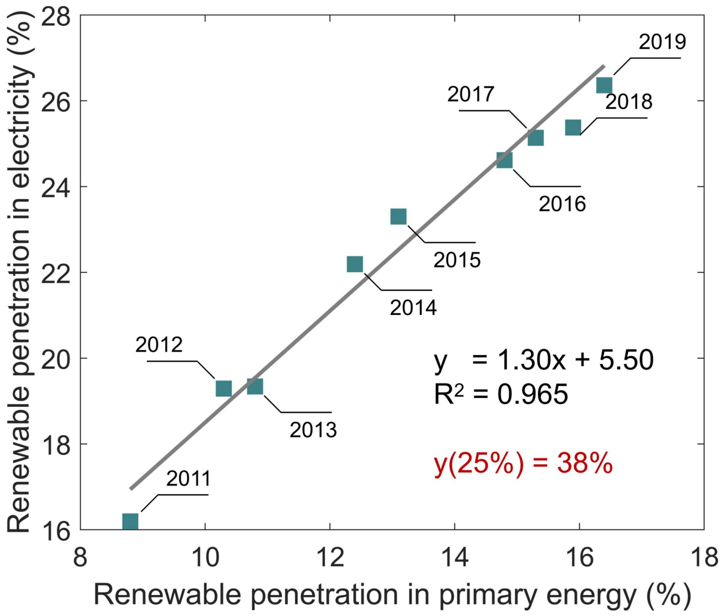

Fig S31. Relationship between the renewable penetration in the electricity sector (RPC) and the non-fossil penetration in primary energy (RPP). Obtained equation could be formulated as RPC = 1.30*RPP + 5.50, with a determination coefficient (R^2) up to 0.965. Based on the obtained fitting equation, 25% RPP corresponds to a RPE valued 38%, which is tagged on the lower right corner.

At the end of 2020, Chinese president Xi JinPing announced a mid-term target of 1200GW non-hydro renewable installations by 2030, targeted to provide 40% of the electricity demand using the contribution from renewables. In response to this national policy, provincial governments released the investment agendas for onshore, offshore wind and solar PV for 2025 and 2030. These investment targets amount to a total of 1228GW by Feb 2022, in line with the national objective. Government planning for each non-hydro renewable (onshore, offshore wind and solar PV) is illustrated in the Table S23. Most provinces definitely proposed the planned capacity for both onshore wind and solar PV in government agenda. A few provinces only released the total capacity planning for onshore wind and solar PV. This capacity is divided into separated onshore wind and solar PV installations proportional to the existing capacity by 2020Q4.

Table S23: latest planning for the non-hydro renewables (onshore, offshore wind and solar PV) illustrated by each provincial government. Total planning (including existing capacity) aggregates to 1228 GW.

Pro	Name	Reg	Updated to 2025			Updated to 2030	Ref
			Wind	Solar	Wind&Solar	Offwind	
Ln	Liaoning	NE	20000	10000	30000	1900	62, 63
Hlj	Heilongjiang	NE	16500	7400	23900	0	64
Saa	Shaanxi	NW	20000	38000	58000	0	65
Qh	Qinghai	NW	16500	42000	58500	0	66
Nx	Ningxia	NW	18500	26000	44500	0	67
Xj	Xinjiang	NW	30000	50000	80000	0	68, 69
Hb	Hebei	NC	43000	54000	97000	5600	70
Sd	Shandong	E	25000	57000	82000	12750	71
Js	Jiangsu	E	26000	26000	52000	14750	72
Zj	Zhejiang	E	6400	27500	33900	6450	73
Sc	Sichuan	C	10000	10000	20000	0	74
Hub	Hubei	C	10000	22000	32000	0	75
Hun	Hunan	C	14000	11000	25000	0	76
Jx	Jiangxi	C	7000	11000	18000	0	77
Gd	Guangdong	S	18000	28000	46000	66850	78
Gx	Guangxi	S	24400	10000	34400	0	76
Gz	Guizhou	S	12000	29000	41000	0	76, 79
Yn	Yunnan	S	20000	5900	25900	0	80
Jl	Jilin	NE	17150	11250	28400	0	81
Im	Inner Mongolia	NC	66350	22350	88700	0	82
Gs	Gansu	NW	29500	20500	50000	0	83
Sx	Shanxi	NC	40700	37200	77900	0	84
Hen	Henan	C	8250	25000	33250	0	85
An	Anhui	E	2900	2000	4900	0	86
Fj	Fujian	E	9000	1800	10800	13300	76
Sh	Shanghai	E	none	none	none	6150	-
Hn	Hainan	S	none	none	none	3950	-

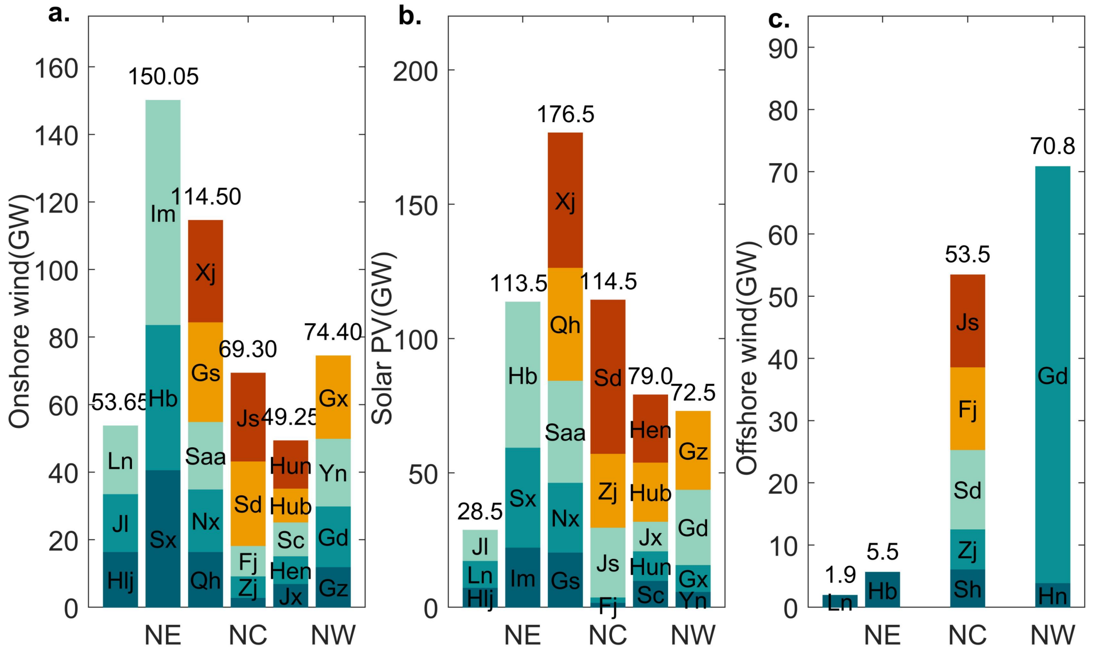

Fig S32: Plans for the onshore, offshore wind and solar PV. Planned amount of onshore, offshore wind and solar PV installations (including the existing capacity), for Northeast China (NE), North China (NC), Northwest China (NW), Eastern China (E), Central China (C) and South China (S).

4.10.2. Scenario settings in 2050

For 2050, four scenarios (S1-S4) are considered here to evaluate the critical factors influencing offshore wind deployment when reaching 80% renewable penetration by 2050: **S1** restricts the offshore wind capacity below 200GW, reflecting the results from previous pathway studies towards 2050 (refer to the Table S24 for details), and utilizing only lithium battery as the storage option; **S2-S4** freely optimize the offshore wind deployment, where **S2** considers lithium battery as the only storage alternative; **S3** allows for the deployments of both lithium batteries and long-term storages such as compressed air (CAES) and pumped hydro (PHES), **S4** further includes the expansion of P2G infrastructures to satisfy the national hydrogen demand. The optimal generation portfolios, deployment scales for different storage technologies, and system costs breakdown under different scenarios are illustrated in Fig5.

Table S24. Scenario settings in 2050

<table>
<tr><th></th><th>S1</th><th>S2</th><th>S3</th><th>S4</th></tr>
<tr><td>Offshore wind</td><td>200GW</td><td colspan="3">Freely optimized</td></tr>
<tr><td>Onshore wind</td><td colspan="4">Freely optimized</td></tr>
<tr><td>Solar PV</td><td colspan="4">Freely optimized</td></tr>
<tr><td>Thermal units</td><td colspan="4">Freely optimized</td></tr>
<tr><td>Transmissions</td><td colspan="4">Freely optimized</td></tr>
<tr><td>Storage</td><td colspan="2">Lithium battery</td><td colspan="2">Li-Battery, PHS, CAES</td></tr>
<tr><td>P2G</td><td colspan="3">0GW</td><td>Optimized</td></tr>
</table>

Restrictions of 200GW offshore wind investment in S1 is derived from previous pathway study and offshore wind development planning. Corresponding researches are presented as below:

Table S25. Offshore wind development plans in previous pathway study and offshore wind development plans. Averaged development capacity (about 200GW) is taken as the baseline scenario in S1.

Off-wind	Timepoint	Mechanism	Ref
40GW	2040	IEA	87
132GW	2050	SGCC-GEIDCO	88
200GW	2050	NDRC	89
300GW	2050	NDRC	90
380GW	2050	IRENA	91

4.10.3. Simulation results

In each simulation, all the investment variables are co-optimized without iteration. Simulation results for Jiangsu and Zhejiang are presented as Fig S33. For the clarity of drawing, 2880 hours are selected (first 240 hours of each month) from the full-year simulation results. Jiangsu has comparable solar PV and offshore wind installations. Local power generation has significant diurnal fluctuation characteristics. While the power system in Zhejiang is dominated by offshore wind power.

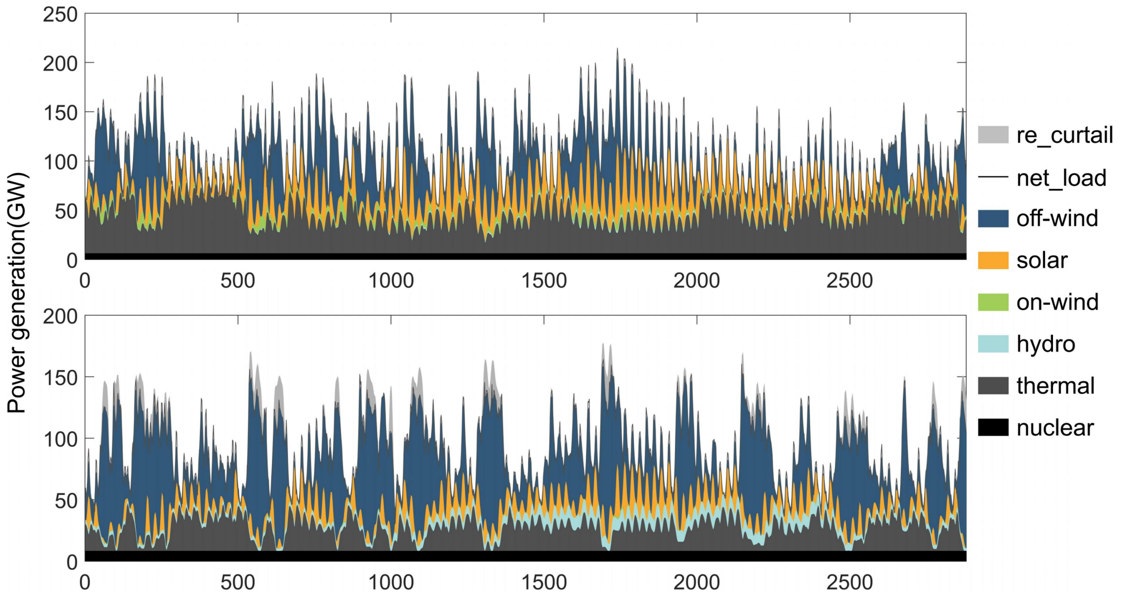

Fig S33. 2880 hours simulation results in Jiangsu (upper) and Zhejiang (bottom). "r_curtail" denotes total curtailment of non-hydro renewables (the onshore, offshore wind and solar PV). "net-load" denotes net load supplied by local generators, calculated as "load-transmission_in+transmission_out-storage_out+storage_in". Local nuclear units remain a constant power generation (90% of the nuclear installations).

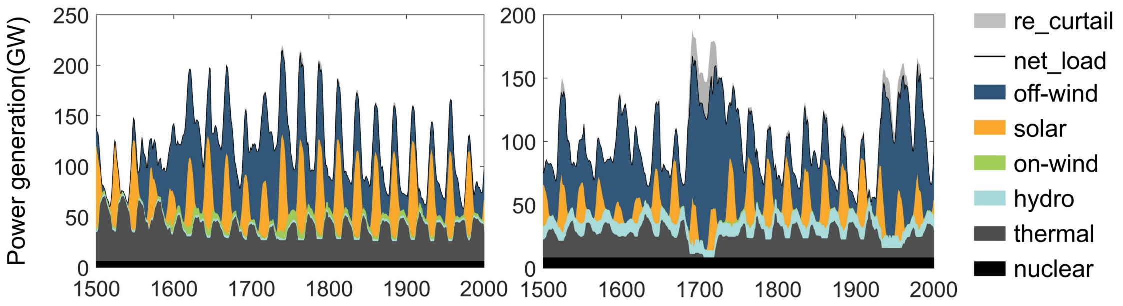

Fig S34. Enlarged figure for the "1500-2000 hour" power system generations in Fig S33, for Jiangsu (left) and Zhejiang (right)

Reference

1. Tracking of offshore wind turbine bidding in 2019. Huachuang securities. http://pdf.dfcfw.com/pdf/H3_AP201911261371159277_1.pdf

2. Michael Maness et al. NREL Offshore Balance-of-System Model. (2017)

https://www.nrel.gov/docs/fy17osti/66874.pdf

3. Philipp Beiter, et al. A Spatial-Economic Cost Reduction Pathway Analysis for U.S. Offshore Wind Energy Development from 2015–2030. (2016).

https://www.nrel.gov/docs/fy16osti/66579.pdf

4. Angel, G, Gonzalez-Rodriguez. Review of offshore wind farm cost components. *Energy for Sustainable Development.* 2017 Volume 37,10-19.

5. M. Zubiaga et al. Energy Transmission and Grid Integration of AC Offshore Wind Farms. March 21st 2012. ISBN: 978-953-51-0368-4.

6. Elliott, Douglas, et al. A Comparison of AC and HVDC Options for the Connection of Offshore Wind Generation in Great Britain. *IEEE Transactions on Power Delivery* (2015):1-1.

7. Dominguez-Garcia, Jose Luis, et al. Effect of non-standard operating frequencies on the economic cost of offshore AC networks. *Renewable Energy* 44(2012):267-280.

8. Offshore wind power series 2. Huachuang securities.

http://pdf.dfcfw.com/pdf/H3_AP201903111304589083_1.pdf

9. Edwaren Liun, et al. Stochastic Methodology to Estimate Costs of HVDC Transmission System. *Journal of Energy and Power Sources*. Vol. 2, No. 3, 2015, pp. 90-98

10. Rentschler, Manuel U. T. et al. Design optimization of dynamic inter-array cable systems for floating offshore wind turbines. *Renewable and Sustainable Energy Reviews.* 111.SEP.(2019):622-635.

11. Rentschler, Manuel U. T. et al. Parametric study of dynamic inter-array cable systems for floating offshore wind turbines. *Marine Systems & Ocean Technology* 15.1(2020):16-25.

12. Vega Luis. Ocean Energy, Introduction. Power Stations Using Locally Available Energy Sources. (2018). ISBN:978-1-4939-7509-9

13. Tasnim Ibn Faiz, and Bhaba R. Sarker. Minimization of transportation and installation time for offshore wind turbines. *International Faim Conference 2014*.

14. M.Asgarpour, R. van de Pieterman. O&M Cost Reduction of Offshore Wind Farms - A Novel Case Study. ECN-E--14-028. https://publicaties.ecn.nl/PdfFetch.aspx?nr=ECN-E--14-028

15. Shafiee Mahmood, F. Brennan, and I. A. Espinosa. A parametric whole life cost model for offshore wind farms. *International Journal of Life Cycle Assessment* 21.7 (2016):961-975.

16. Sun BoYang, Yang XiaoHua, Huang JieTing and Yang XiaoHua. China Offshore Wind Power Costs and Environmental Impact Analysis. *International Conference on Sustainable Development 2017.*

17. Review of existing cost and O&M models, and development of a highfidelity cost/revenue model for impact assessment. (2018). https://www.romeoproject.eu/wp-content/uploads/2018/12/D8.1_ROMEO_Report-reviewing-exsiting-cost-and-OM-support-models.pdf

18. 2018 Cost of Wind Energy Review. (2019). NREL. https://www.nrel.gov/docs/fy20osti/74598.pdf

19. Walter Musial, et al. Oregon Offshore Wind Site Feasibility and Cost Study. (2019). NREL.

https://www.nrel.gov/docs/fy20osti/74597.pdf

20. Special report on power equipment industry. (2019). PingAn Securities.

http://pg.jrj.com.cn/acc/Res/CN_RES/INDUS/2019/1/21/e4fe11a9-e04c-4754-b515-5c37c4fffabb.pdf

21. Gao, Xiaoxia , H. Yang , and L. Lu . "Optimization of wind turbine layout position in a wind farm using a newly-developed two-dimensional wake model." *Applied Energy* 174(2016):192-200.

22. Pérez, Beatriz, Mínguez, Roberto, and Guanche, Raúl. Offshore wind farm layout optimization using mathematical programming techniques. *Renewable Energy* 53. MAY(2013):389-399.

23. Ahsbahs, T. , Badger, M. , Volker, P. , Hansen, K. S. , & Hasager, C. B. . (2018). Applications of satellite winds for the offshore wind farm site anholt. *Wind Energ. Sci*. 3, 573–588, 2018

24. Yuan-Kang, Wu, et al. "Optimization of the wind turbine layout and transmission system planning for a large-scale offshore wind farm by AI technology."

Industry Applications Society Meeting IEEE, 2012.

25. Sherman, Peter & Chen, Xinyu & McElroy, Michael. (2020). Offshore wind: An opportunity for cost-competitive decarbonization of China's energy economy. Science Advances. 6. eaax9571. 10.1126/sciadv.aax9571.

26. W. Musial, D. Heimiller, P. Beiter, G. Scott, C. Draxl, 2016 Offshore Wind Energy Resource Assessment for the United States. Tech. Rep. (NREL/TP-5000-66599, 2016).

27. The Modern-Era Retrospective Analysis for Research and Applications, version 2.

https://disc.gsfc.nasa.gov/dataset

28. Oceans One Minute Grid. www.gebco.net

29. Xingning Han, Xinyu Chena, Michael B. McElroyb, Shiwu Liaod, Chris P. Nielsenb, Jinyu Wen.Modeling formulation and validation for accelerated simulation and flexibility assessment on large scale power systems under higher renewable penetrations. Applied Energy 237 (2019) 145–154

30. X. Chen, J. Lv, M. B. McElroy, X. Han, C. P. Nielsen and J. Wen, "Power System Capacity Expansion Under Higher Penetration of Renewables Considering Flexibility Constraints and Low Carbon Policies," in IEEE Transactions on Power Systems, vol. 33, no. 6, pp. 6240-6253, Nov. 2018, doi: 10.1109/TPWRS.2018.2827003.

31. X. Chen, M. B. McElroy and C. Kang, "Integrated Energy Systems for Higher Wind Penetration in China: Formulation, Implementation, and Impacts," in *IEEE Transactions on Power Systems*, vol. 33, no. 2, pp. 1309-1319, March 2018, doi: 10.1109/TPWRS.2017.2736943.

32. Haiwang Zhong, Qing Xia, Yuguo Chen and Chongqing Kang. Energy-Saving Generation Dispatch Toward Sustainable Electric Power Industry. *Energy Policy*, 2015, 83: 14-25

33. IMCEC. National and provincial electricity coal price index. (2018).

http://www.imcec.cn/zgdm_2018

34. National Development and Reform Commission. Price list of natural gas benchmark in all provinces. (2018).

https://www.ndrc.gov.cn/xxgk/zcfb/ghxwj/201805/t20180525_960947.html

35. Duenas, P. , Leung, T. , Gil, M. , & Reneses, J. . (2015). Gas–electricity coordination in competitive markets under renewable energy uncertainty. IEEE Transactions on Power Systems, 30(1), 123-131.

36. Zhang, N. , Lu, X. , Mcelroy, M. B. , Nielsen, C. P. , Chen, X. , & Deng, Y. , et al. (2015). Reducing curtailment of wind electricity in china by employing electric boilers for heat and pumped hydro for energy storage. Applied Energy, S0306261915013896.

37. Gang He, Anne-Perrine Avrin, James H. Nelson, Josiah Johnston, Ana Mileva, Jianwei Tian and Daniel M. Kammen. SWITCH-China: A Systems Approach to Decarbonize China's Power System. *Environmental Science & Technology* (2016)

38. X. Chen, M. B. McElroy and C. Kang, "Integrated Energy Systems for Higher Wind Penetration in China: Formulation, Implementation, and Impacts," in *IEEE Transactions on Power Systems*, vol. 33, no. 2, pp. 1309-1319, March 2018, doi: 10.1109/TPWRS.2017.2736943.

39. State Grid Corporation of China. Research on power demand forecasting. (2013)

40. State Power Regulatory Commission. Notice on cost supervision of power engineering projects put into operation during the 11th Five-Year Plan Period.

https://wenku.baidu.com/view/b8d23bb8960590c69fc37600.html#

41. Huang Canran, Zhang Shijie, Wang Bo, Xiao Yunhan. (2010). Investment cost model of gas turbine generator set. Gas turbine technology (02), 21-28

42. China Energy Statistics Yearbook. (2020).

https://data.cnki.net/trade/Yearbook/Single/N2021050066?z=Z024

43. The State Grid Corporation of China issued the action plan of "carbon peaking and carbon neutralization". State Grid Corporation of China (SGCC). 2021.

http://download.caixin.com/upload/gjdwtzh.pdf

44. Research on China's electric power development planning in the 14th five year plan. Global energy Internet Development Cooperation Organization. 2020.

https://upload.geidco.org.cn/2020/0801/1596270079592.pdf

45. Under the transformation of energy structure, UHV construction is about to accelerate. 2022. https://m.gelonghui.com/p/506325

46. New infrastructure began to work, and UHV ushered in a good opportunity for layout. 2020. http://pdf.dfcfw.com/pdf/H3_AP202004081377763195_1.pdf

47. Construction of the 9 major power transmission and transformation projects including UHV will start. 2018. http://pdf.dfcfw.com/pdf/H3_AP201809131194591426_1.pdf

48. New news from Sichuan Chongqing UHV. National energy information platform. 2021. https://baijiahao.baidu.com/s?id=1693118608820868560&wfr=spider&for=pc

49. Help Sichuan and Chongqing to develop the 21 billion Y-shaped 1000kV high voltage project, which will be put into construction soon. 2020. https://www.sohu.com/a/410482037_100072433

50. Han Xiancai et al. Summary of the development of UHV AC transmission engineering technology in China. Chinese Journal of electrical engineering. 2020. http://www.csee.org.cn/pic/u/cms/www/202103/11102000nfmr.pdf

51. Environmental impact report of Nanyang Jingmen Changsha UHV AC power transmission and transformation project. State Grid Corporation of China.

http://www.dwhbkp.com/uploads/soft/20200408/1586318949.pdf

52. Environmental impact report of changes in Zhumadian Wuhan UHV AC power transmission and transformation project. State Grid Corporation of China.

http://www.hb.sgcc.com.cn/html/main/col1000/2021-09/06/20210906143440708268981_1.html

53. White paper on China's hydrogen energy and fuel cell industry. China Hydrogen Alliance.

(2019). http://h2cn.org.cn/publicati/215.html

54. Technology Data for Renewable Fuels. Danish Energy Agency. (2021 update).

https://ens.dk/en/our-services/projections-and-models/technology-data/technology-data-renewable-fuels

55. Bart W. Tuinema et al. Modelling of Large-Size Electrolysers for Real-Time Simulation and Study of the Possibility of Frequency Support by Electrolysers. (2020).

56. J. Tijdgat. Shipping renewable hydrogen carriers - a study on the impact of shipping renewable hydrogen carriers and using those as a fuel on, the ship design, the different powertrain configurations, and the cost of transported hydrogen. Master thesis submitted to the Delft University of Technology. (2020).

57. How low can the cost of hydrogen be - storage and transportation. GZ Hang Seng. (2019).

58. The Future of Hydrogen - Report extract Data and assumptions. IEA. (2019) https://www.iea.org/reports/the-future-of-hydrogen/data-and-assumptions

59. Carbon peak action plan before 2030. State Council of China. (2021) http://www.gov.cn/zhengce/content/2021-10/26/content_5644984.htm

60. China's renewable power generation reached 2.2 trillion kWh in 2020. National Energy Administration. (2021). https://www.chinanews.com.cn/cj/2021/03-30/9443384.shtml

61. Solicit opinions on the weight of renewable energy power consumption responsibility of all provinces from 2021 to 2030. National Energy Administration. (2021). https://chuneng.bjx.com.cn/news/20210210/1135946.shtml

62. With a total investment of more than 800 billion yuan and 48 major projects, our province has accelerated the layout of clean energy to help green and low-carbon development. Liaoning Provincial People's government. 2021. http://www.ln.gov.cn/qmzx/gclsxjpzsjjhjs/gjfzxgj/202104/t20210416_4116881.html

63. The 14th five year plan for national economic and social development of Liaoning Province and the outline of long-term objectives for 2035. Liaoning Provincial People's government. https://www.ndrc.gov.cn/fggz/fzzlgh/dffzgh/202106/t20210628_1284321.html

64. The 14th five year plan for national economic and social development of Heilongjiang Province and the outline of long-term objectives for 2035. Heilongjiang Provincial People's government. https://www.ndrc.gov.cn/fggz/fzzlgh/dffzgh/202106/t20210628_1284318.html

65. The 14th five year plan for national economic and social development of Shaanxi Province and the outline of long-term objectives for 2035. Shaanxi Provincial People's government. https://www.ndrc.gov.cn/fggz/fzzlgh/dffzgh/202104/t20210427_1277523.html

66. Notice of the National Energy Administration on printing and distributing the action plan for Qinghai to build a national highland of clean energy industry (2021-2030). Qinghai Provincial

People's government.

http://fgw.qinghai.gov.cn/qhsfgwWAP/xxgk/zdgkml/fgwwj/202108/t20210812_78615.html

67. The 14th five year plan for national economic and social development of Ningxia Province and the outline of long-term objectives for 2035. Ningxia Provincial People's government.

https://www.ndrc.gov.cn/fggz/fzzlgh/dffzgh/202104/t20210428_1277932.html?code=&state=123

68. It is estimated that the installed capacity of new wind power in Xinjiang will exceed 10 million kW during the 14th Five Year Plan period.

https://news.bjx.com.cn/html/20201028/1112408.shtml

69. The installed capacity of renewable energy in the 14th five year plan is 82.4 million kW. Xinjiang Provincial People's Government.
https://guangfu.bjx.com.cn/news/20210220/1137060.shtml

70. The 14th five year plan for national economic and social development of Hebei Province and the outline of long-term objectives for 2035. Hebei Provincial People's government.

https://www.ndrc.gov.cn/fggz/fzzlgh/dffzgh/202106/t20210611_1283092.html

71. The 14th five year plan for energy development in Shandong Province.

https://www.ndrc.gov.cn/fggz/fzzlgh/dffzgh/202105/t20210513_1279758.html?code=&state=123

72. The 14th five year plan for energy development in Jiangsu Province.

https://guangfu.bjx.com.cn/news/20210108/1128008.shtml

73. The 14th five year plan for renewable energy development in Jiangsu Province

https://news.bjx.com.cn/html/20210624/1160169.shtml

74. Notice of Sichuan Provincial Energy Bureau on printing and distributing several guiding opinions on the development of solar PV and wind power resources in Sichuan Province during the 14th five year plan ([2021] No. 181). Sichuan Provincial Development and Reform Commission.
http://fgw.sc.gov.cn/sfgw/c106099/2021/6/16/7aa6091d6c20491aabaa87345e548d2d.shtml

75. Hubei has issued the "14th five year plan" for green development of the Yangtze River economic belt. It plans to basically build a green economic system by 2025.

https://cjlt.ntu.edu.cn/2021/1215/c5384a183397/page.htm

76. 24.43 million kW: during the 14th Five Year Plan period, Guangxi will be able to ride the wind power in the southern provinces. China Energy Network.

https://www.china5e.com/news/news-1117923-1.html

77. The 14th five year plan for national economic and social development of Jiangxi Province and the outline of long-term objectives for 2035. Jiangxi Provincial People's government.

https://www.ndrc.gov.cn/fggz/fzzlgh/dffzgh/202105/t20210508_1279408.html

78. Guangdong ecological civilization 14th five year plan. Guangdong People's Government

http://www.gd.gov.cn/zwgk/wjk/qbwj/yf/content/post_3595207.html

79. The construction of new power system in Guizhou is in full swing. Guizhou Daily.

http://gz.news.cn/2021-12/13/c_1128157018.htm

80. The 14th five year plan for national economic and social development of Yunnan Province and the outline of long-term objectives for 2035. Yunnan Provincial People's government.

https://www.ndrc.gov.cn/fggz/fzzlgh/dffzgh/202105/t20210508_1279410.html

81. Promote the "Three Gorges on land" project! Jilin plans to have 30 million kilowatts of new energy installed by 2025.

http://www.cspplaza.com/article-20919-1.html

82. By 2025, the installed capacity of new energy power generation in Inner Mongolia Autonomous Region will account for more than 50% of the total installed capacity. State Grid news. https://wind.in-en.com/html/wind-2401644.shtml

83. The 14th five year plan for national economic and social development of Gansu Province and the outline of long-term objectives for 2035. Gansu Provincial People's government.

https://www.ndrc.gov.cn/fggz/fzzlgh/dffzgh/202105/t20210519_1280212.html?code=&state=123

84. Shanxi: it is expected that the installed capacity of new energy will double by 2025, accounting for more than 40%. https://guangfu.bjx.com.cn/news/20210909/1175947.shtml

85. Henan: in 2025, the installed capacity of renewable energy will reach more than 50gw, and strive to add 20GW of wind and light. https://guangfu.bjx.com.cn/news/20210412/1146625.shtml

86. www.djyanbao.com/preview/268520

87. World energy outlook, China Special Report. International Energy Agency (IEA). 2017.

https://iea.blob.core.windows.net/assets/bfe62afd-3fa0-4d23-ba56-caae787c0a1c/WEO_2017_Special_Report_China.pdf

88. Research on China's electric power development planning in the 14th five year plan.

State Grid Corporation of China (SGCC) - Global Energy Interconnection Development and Cooperation Organization. 2020.

https://upload.geidco.org.cn/2020/0801/1596270079592.pdf

89. China's wind power development roadmap 2050.

Energy Research Institute. National Development and Reform Commission (NDRC).

http://wbmngo.oss-cn-beijing.aliyuncs.com/images/companyNewsImages/1578986179261.pdf

90. Research on the development scenario and path of China's 2050 high proportion renewable energy. Energy Research Institute. National Development and Reform Commission (NDRC). 2015.

https://www.efchina.org/Attachments/Report/report-20150420/%E4%B8%AD%E5%9B%BD2050%E9%AB%98%E6%AF%94%E4%BE%8B%E5%8F%AF%E5%86%8D%E7%94%9F%E8%83%BD%E6%BA%90%E5%8F%91%E5%B1%95%E6%83%85%E6%99%AF%E6%9A%A8%E9%80%94%E5%BE%84%E7%A0%94%E7%A9%B6-%E5%AE%A3%E4%BC%A0%E5%86%8C.pdf

91. FUTURE OF WIND. The International Renewable Energy Agency (IRENA). 2019.

https://www.irena.org/-/media/files/irena/agency/publication/2019/oct/irena_future_of_wind_2019.pdf
