# Supplementary material for: Grid integration feasibility and investment planning of offshore wind power under carbon-neutral transition in China
Source: Nat Commun. 2023 Apr 28;14:2447. doi: 10.1038/s41467-023-37536-3 (PMC10141809; doi:10.1038/s41467-023-37536-3)
Supplement: Supplementary file 2 — Reporting Summary [file 41467_2023_37536_MOESM2_ESM.pdf]

nature portfolio

Corresponding author(s): Xinyu Chen
Jinyu Wen
Michael McElroy

Last updated by author(s): Oct 8, 2022

Reporting Summary

Nature Portfolio wishes to improve the reproducibility of the work that we publish. This form provides structure for consistency and transparency in reporting. For further information on Nature Portfolio policies, see our Editorial Policies and the Editorial Policy Checklist.

Statistics

For all statistical analyses, confirm that the following items are present in the figure legend, table legend, main text, or Methods section.

n/a	Confirmed	
☐	☒	The exact sample size (*n*) for each experimental group/condition, given as a discrete number and unit of measurement
☒	☐	A statement on whether measurements were taken from distinct samples or whether the same sample was measured repeatedly
☒	☐	The statistical test(s) used AND whether they are one- or two-sided<br>*Only common tests should be described solely by name; describe more complex techniques in the Methods section.*
☒	☐	A description of all covariates tested
☐	☒	A description of any assumptions or corrections, such as tests of normality and adjustment for multiple comparisons
☒	☐	A full description of the statistical parameters including central tendency (e.g. means) or other basic estimates (e.g. regression coefficient) AND variation (e.g. standard deviation) or associated estimates of uncertainty (e.g. confidence intervals)
☒	☐	For null hypothesis testing, the test statistic (e.g. *F*, *t*, *r*) with confidence intervals, effect sizes, degrees of freedom and *P* value noted<br>*Give P values as exact values whenever suitable.*
☒	☐	For Bayesian analysis, information on the choice of priors and Markov chain Monte Carlo settings
☒	☐	For hierarchical and complex designs, identification of the appropriate level for tests and full reporting of outcomes
☒	☐	Estimates of effect sizes (e.g. Cohen's *d*, Pearson's *r*), indicating how they were calculated

Our web collection on statistics for biologists contains articles on many of the points above.

Software and code

Policy information about availability of computer code

Data collection	no software was used
Data analysis	(a) MATLAB R2022a: power system model construction, result processing and figure drawing.<br>(b) Yalmip R20210331: model conversion. (c) Gurobi R9.5.1: power system model solving.

For manuscripts utilizing custom algorithms or software that are central to the research but not yet described in published literature, software must be made available to editors and reviewers. We strongly encourage code deposition in a community repository (e.g. GitHub). See the Nature Portfolio guidelines for submitting code & software for further information.

Data

Policy information about availability of data

All manuscripts must include a data availability statement. This statement should provide the following information, where applicable:

- Accession codes, unique identifiers, or web links for publicly available datasets
- A description of any restrictions on data availability
- For clinical datasets or third party data, please ensure that the statement adheres to our policy

The data employed in this study is available from the lead contact on reasonable request.

Human research participants

Policy information about studies involving human research participants and Sex and Gender in Research.

Reporting on sex and gender	This research is not related to sex-and gender-. The information about sex- and gender- has not been collected in this study.
Population characteristics	This study does not involve population participation.
Recruitment	This study does not involve population participation.
Ethics oversight	This study does not involve human research, and there is no human research related organization involved.

Note that full information on the approval of the study protocol must also be provided in the manuscript.

Field-specific reporting

Please select the one below that is the best fit for your research. If you are not sure, read the appropriate sections before making your selection.

☐ Life sciences ☐ Behavioural & social sciences ☒ Ecological, evolutionary & environmental sciences

For a reference copy of the document with all sections, see nature.com/documents/nr-reporting-summary-flat.pdf

Ecological, evolutionary & environmental sciences study design

All studies must disclose on these points even when the disclosure is negative.

Study description	We present a high-resolution assessment model to conduct a system integration analysis designed to develop an optimal deployment plan for offshore wind power in China. First, the physical characteristics and economics of offshore wind projects are analyzed for all possible locations off the coast of mainland China. Second, hourly grid integration simulations are conducted for a full year at all possible onshore and offshore wind investment levels for all of the provinces in the coastal area and in the wind-rich "Three North" region. Third, the analysis further includes an optimal investment model for thermal units, non-hydro renewables, transmissions, storage systems and P2G facilities, intended to complement system investment planning for both 2030 and 2050.
Research sample	Our research does not involve samples, and the data sources we use are shown in "Data collection" section below.
Sampling strategy	We did not employ the sampling method because all the data were included in the power system simulation.
Data collection	(1) For offshore wind economics analysis: the contract prices for offshore turbines and overall projects were collected from public data sources, which have been indicated in method and SI section.<br>(2) For offshore wind resources analysis: offshore wind data is derived from NASA's MERRA2 dataset; turbine power output is calculated based on the typical power curve of the MHI-Vestas V164-8MW offshore turbine model; ocean depth data is taken from GEBCO One Minute Grid; China's EEZ data is derived from Marine Regions Dataset; Special Maritime Reserves are calibrated by the National Maritime Dataset; shipping lanes are estimated using SO2 emission data derived from the MERRA-2 dataset as a surrogate identification.<br>(3) For the power system model: detailed operational parameters for over 3000 thermal units are obtained from internal data; natural inflow for all major hydro power stations is determined based on the CFSR Dataset; real-world provincial load demand is provided by State Grid Corporation of China; existing inter-provincial transmissions are collected from both public sources and internal Dataset, with the public data sources indicated in the SI section; planned inter-provincial transmissions are collected from public sources, which is indicated in the SI section; government planning of all the provinces for each non-hydro renewable is collected from public sources, which is listed province-by-province in the SI section. Information for each hydropower station is collected from Liu, Hailiang, et al, "A validated high-resolution hydro power time-series model for energy systems analysis. (2019)". The fuel consumption relationship of thermal units refers to the researches proposed by Xinyu Chen et al, "Integrated Energy Systems for Higher Wind Penetration in China: Formulation, Implementation, and Impacts".<br>(4) For the cost data adopted, offshore wind capital expenditure is modeled as noted in the "Numerical Simulation and Cost Estimation for Offshore Wind Farms" subsection in method and SI. The capital expenditure for onshore wind and solar PV is evaluated on a provincial basis according to the Manual of Renewable Energy Data 2015 released by the National Energy Administration. The cost for solar PV is derived from the Annual Technology Baseline available from NREL. The cost for each storage option in both 2030 and 2050 is obtained using the estimates for energy storage Technology Data published by the Danish Energy Agency.
Timing and spatial scale	We present a high-resolution assessment model to conduct a system integration analysis designed to develop an optimal deployment plan for offshore wind power in China.<br>(1) The timing scale of the code includes three milestone years (2020, 2030 and 2050). For each milestone year, the simulation is conducted at the hourly basis throughout an entire year. For the system integration study in 2020, power system simulation is conducted on an hourly basis throughout an entire year, simulating and optimizing the system operations while estimating the potential curtailment rates. For the optimal investment analyses in 2030 and 2050, this investment model optimizes the allocation of all the non-hydro renewables (onshore, offshore wind and solar PV), thermal units, transmissions, storages and P2G facilities at

	hourly basis in a year.<br>(2) The spatial scale of this study incorporates provincial-level operations for the entire China.
Data exclusions	No data is excluded.
Reproducibility	The code employed in this study is available from the lead contact on reasonable request. The results are repeatable adopting related code.
Randomization	The randomness is not relevant to our study. Our research simulates and optimizes the operations of entire power system. We do not use partial sample data to run the simulation.
Blinding	The blinding is not relevant to our study. Our study utilizes the real-world power system operation data, simulates and optimizes the operation of the entire power system.

Did the study involve field work? ☐ Yes ☒ No

Reporting for specific materials, systems and methods

We require information from authors about some types of materials, experimental systems and methods used in many studies. Here, indicate whether each material, system or method listed is relevant to your study. If you are not sure if a list item applies to your research, read the appropriate section before selecting a response.

Materials & experimental systems

n/a	Involved in the study
☒	☐ Antibodies
☒	☐ Eukaryotic cell lines
☒	☐ Palaeontology and archaeology
☒	☐ Animals and other organisms
☒	☐ Clinical data
☒	☐ Dual use research of concern

Methods

n/a	Involved in the study
☒	☐ ChIP-seq
☒	☐ Flow cytometry
☒	☐ MRI-based neuroimaging
